# Supplementary material for: Factors influencing implementation and sustainability of interventions to improve oral health and related health behaviours in adults experiencing severe and multiple disadvantage: a mixed-methods systematic review
Source: BMJ Open. 2024 Jan 12;14(1):e080160. doi: 10.1136/bmjopen-2023-080160 (PMC10806606; doi:10.1136/bmjopen-2023-080160)
Supplement: Supplementary data [file bmjopen-2023-080160supp001.pdf]

SMD Project – Search updates February 2023

Database(s): **Ovid MEDLINE(R) and Epub Ahead of Print, In-Process, In-Data-Review & Other Non-Indexed Citations, Daily and Versions** 1946 to January 30th 2023 **(Search run 3rd February 2023)**

Search Strategy:

#	Searches	Results
1	Oral Hygiene/	13719
2	Mouth Rehabilitation/	1470
3	Oral Health/	19795
4	exp Dental Health Services/	39658
5	((dental or oral or tooth or teeth or mouth) adj3 (health or care or hygiene or rehabilitation)).ti,ab,kw.	71845
6	or/1-5	105093
7	Smoking/	147692
8	(smoking or cigarette* or tobacco).ti,ab,kw.	346981
9	exp "Tobacco Use"/	9080
10	Alcohol Drinking/	74302
11	Alcoholism/	79526
12	Alcoholics/	871
13	(alcoholic* adj3 (person or people or adult or parent* or family)).ti,ab,kw.	947
14	"street drink*".ti,ab,kw.	8
15	exp Substance-Related Disorders/	306459
16	exp Drug users/	3922
17	Behavior, Addictive/	12185
18	((alcohol or drug* or substance*) adj2 (misuse* or abuse* or use* or addict* or dependenc* or issue* or problem*)).ti,ab,kw.	314183
19	(drug adj1 (habit or tak* or hard or illicit or inject*)).ti,ab,kw.	25327
20	or/7-19	873304
21	((sugar or sucrose or fructose or glucose) adj2 (intake or consum*)).ti,ab,kw.	16517

22	diet*.ti,ab,kw.	665093
23	"sugary food*".ti,ab,kw.	328
24	exp Dietary Sugars/ or Diet/	186792
25	((processed or acidic) adj1 food*).ti,ab,kw.	5331
26	((sugary or fizzy or carbonated or soft) adj1 drink*).ti,ab,kw.	5449
27	carbonated beverages/ or sugar-sweetened beverages/	3952
28	soda.ti,ab,kw.	4878
29	or/21-28	738771
30	(severe and multiple disadvantage*).ti,ab,kw.	11
31	Homeless Persons/	9442
32	homeless*.ti,ab,kw.	13348
33	((hous* or home* or accommodat* or shelter) adj3 (insecur* or instability or unstable or stability)).ti,ab,kw.	4473
34	or/30-33	18951
35	(probationer* or parolee* or ((repeat* or ex or re or revolving door or habitual or multiple or former* or previously*) adj1 (offen* or convict* or prisoner* or imprison* or incarcerat* or criminal*)) or (former adj3 inmate*) or ((community or probation* or parole* or reintegrat*) adj4 (prison* or offender* or criminal* or convict* or inmate*)) or ((individuals or men or women) adj2 (probation or parole)) or ((reintegrate* or reent* or return*) adj3 community)).ti,ab,kw.	4639
36	"criminal justice".ti,ab,kw.	5400
37	or/35-36	9464
38	((program* or policy or policies or strateg* or scheme* or project* or initiative* or "care package" or training or educat* or pilot or guidance or guideline* or study or pathway or treatment* or promot* or management or "support group" or process* or trial* or intervention*) adj5 (evaluat* or effect* or measur* or assess* or experiment* or impact* or feasab* or acceptab* or efficacy or perception* or belief* or uptake or consequence* or attitud* or barrier* or facilit* or motivat* or experience* or implement* or adher* or retention or retain* or reduc* or increas* or improv* or outcome* or cost* or benefit* or interview* or qualitative or ethnograph* or "focus group*")).ti,ab,kw.	5409160
39	"housing first".ti,ab,kw.	388
40	(outcome* adj5 evaluat*).ti,ab,kw.	106574

41	or/38-40	5450432
42	(34 or 37) and (6 or 20 or 29)	10263
43	41 and 42	5069

Database(s): **Embase** 1974 to 2023 January 30 **(Search run 3rd January 2023)**

Search Strategy:

#	Searches	Results
1	mouth hygiene/	30848
2	full mouth rehabilitation/	367
3	dental health/	4854
4	dental procedure/	30979
5	dental practice/ or dental prevention/ or exp dental restoration/	69380
6	((dental or oral or tooth or teeth or mouth) adj3 (health or care or hygiene or rehabilitation)).ti,ab,kw.	74077
7	or/1-6	169561
8	smoking/	360231
9	(smoking or cigarette* or tobacco).ti,ab,kw.	482602
10	"tobacco use"/	16485
11	drinking behavior/	54555
12	alcoholism/	128949
13	(alcoholic* adj3 (person or people or adult or parent* or family)).ti,ab,kw.	1320
14	"street drink*".ti,ab,kw.	15
15	exp drug dependence/	266514
16	addiction/	50440
17	((alcohol or drug* or substance*) adj2 (misuse* or abuse* or use* or addict* or dependenc* or issue* or problem*)).ti,ab,kw.	438421
18	(drug adj1 (habit or tak* or hard or illicit or inject*)).ti,ab,kw.	33544
19	or/8-18	1177022

20	((sugar or sucrose or fructose or glucose) adj2 (intake or consum*)).ti,ab,kw.	21137
21	diet*.ti,ab,kw.	830039
22	"sugary food*".ti,ab,kw.	432
23	sugar intake/	9669
24	diet/	244115
25	((processed or acidic) adj1 food*).ti,ab,kw.	6402
26	((sugary or fizzy or carbonated or soft) adj1 drink*).ti,ab,kw.	7130
27	carbonated beverage/	3672
28	sugar-sweetened beverage/	2782
29	or/20-28	902120
30	(severe and multiple disadvantage*).ti,ab,kw.	14
31	exp homeless person/	3638
32	homeless*.ti,ab,kw.	16533
33	((hous* or home* or accommodat* or shelter) adj3 (insecur* or instability or unstable or stability)).ti,ab,kw.	5665
34	or/30-33	21932
35	(probationer* or parolee* or ((repeat* or ex or re or revolving door or habitual or multiple or former* or previously*) adj1 (offen* or convict* or prisoner* or imprison* or incarcerat* or criminal*)) or (former adj3 inmate*) or ((community or probation* or parole* or reintegrat*) adj4 (prison* or offender* or criminal* or convict* or inmate*)) or ((individuals or men or women) adj2 (probation or parole)) or ((reintegrate* or reent* or return*) adj3 community)).ti,ab,kw.	5563
36	"criminal justice".ti,ab,kw.	6462
37	or/35-36	11379
38	((program* or policy or policies or strateg* or scheme* or project* or initiative* or "care package" or training or educat* or pilot or guidance or guideline* or study or pathway or treatment* or promot* or management or "support group" or process* or trial* or intervention*) adj5 (evaluat* or effect* or measur* or assess* or experiment* or impact* or feasab* or acceptab* or efficacy or perception* or belief* or uptake or consequence* or attitud* or barrier* or facilit* or motivat* or experience* or implement* or adher* or retention or retain* or reduc* or increas* or improv* or outcome* or cost* or benefit* or interview* or qualitative or ethnograph* or "focus group*")).ti,ab,kw.	7390414

39	"housing first".ti,ab,kw.	444
40	(outcome* adj5 evaluat*).ti,ab,kw.	168129
41	or/38-40	7453916
42	(34 or 37) and (7 or 19 or 29)	12372
43	41 and 42	6384

Database(s): **APA PsycInfo** 1806 to January Week 4 2023 **(Search run 3rd February 2023)**

Search Strategy:

#	Searches	Results
1	exp oral health/	1890
2	exp dental health/	594
3	((dental or oral or tooth or teeth or mouth) adj3 (health or care or hygiene or rehabilitation)).ti,ab.	3199
4	or/1-3	3613
5	tobacco smoking/	35146
6	(smoking or cigarette* or tobacco).ti,ab.	69114
7	alcohol drinking patterns/	26122
8	Alcoholism/	30862
9	alcohol abuse/	19136
10	(alcoholic* adj3 (person or people or adult or parent* or family)).ti,ab.	1753
11	"street drink*".ti,ab.	19
12	"substance use disorder"/	10378
13	drug abuse/	49214
14	drug addiction/	11973
15	addiction/	12827
16	((alcohol or drug* or substance*) adj2 (misuse* or abuse* or use* or addict* or dependenc* or issue* or problem*)).ti,ab.	183254
17	(drug adj1 (habit or tak* or hard or illicit or inject*)).ti,ab.	12930

18	or/5-17	279147
19	((sugar or sucrose or fructose or glucose) adj2 (intake or consum*)).ti,ab.	2160
20	diet*.ti,ab.	47688
21	"sugary food*".ti,ab.	62
22	Sugars/	2537
23	diets/	14955
24	((processed or acidic) adj1 food*).ti,ab.	396
25	((sugary or fizzy or carbonated or soft) adj1 drink*).ti,ab.	961
26	soda.ti,ab.	513
27	or/19-26	53562
28	(severe and multiple disadvantage*).ti,ab.	10
29	Homeless/	8126
30	homeless*.ti,ab.	12396
31	((hous* or home* or accommodat* or shelter) adj3 (insecur* or instability or unstable or stability)).ti,ab.	2080
32	or/28-31	14528
33	(probationer* or parolee* or ((repeat* or ex or re or revolving door or habitual or multiple or former* or previously*) adj1 (offen* or convict* or prisoner* or imprison* or incarcerat* or criminal*)) or (former adj3 inmate*) or ((community or probation* or parole* or reintegrat*) adj4 (prison* or offender* or criminal* or convict* or inmate*)) or ((individuals or men or women) adj2 (probation or parole)) or ((reintegrate* or reent* or return*) adj3 community)).ti,ab.	9014
34	"criminal justice".ti,ab.	13327
35	or/33-34	21049
36	((program* or policy or policies or strateg* or scheme* or project* or initiative* or "care package" or training or educat* or pilot or guidance or guideline* or study or pathway or treatment* or promot* or management or "support group" or process* or trial* or intervention*) adj5 (evaluat* or effect* or measur* or assess* or experiment* or impact* or feasab* or acceptab* or efficacy or perception* or belief* or uptake or consequence* or attitud* or barrier* or facilit* or motivat* or experience* or implement* or adher* or retention or retain* or reduc* or increas* or improv* or outcome* or cost* or benefit* or interview* or qualitative or ethnograph* or "focus group*")).ti,ab.	1427116

37	"housing first".ti,ab.	354
38	(outcome* adj5 evaluat*).ti,ab.	15300
39	or/36-38	1431044
40	(32 or 35) and (4 or 18 or 27)	8717
41	39 and 40	4427

CINAHL (via Ebsco) **Friday, February 3, 2023 12:16:53 PM**

**#**	**Query**	**Results**
S44	S41 AND S42 AND S43	3,512
S43	S5 or S18 or S29	551,298
S42	S34 or S37	20,783
S41	S38 OR S39 OR S40	1,823,817
S40	TI (outcome* N5 evaluat*) OR AB (outcome* N5 evaluat*)	43,464
S39	TI "housing first" OR AB "housing first"	327
S38	TI (((program* or policy or policies or strateg* or scheme* or project* or initiative* or "care package" or training or educat* or pilot or guidance or guideline* or study or pathway or treatment* or promot* or management or "support group" or process* or trial* or intervention*) N5 (evaluat* or effect* or measur* or assess* or experiment* or impact* or feasab* or acceptab* or efficacy or perception* or belief* or uptake or consequence* or attitud* or barrier* or facilit* or motivat* or experience* or implement* or adher* or retention or retain* or reduc* or increas* or improv* or outcome* or cost* or benefit* or interview* or qualitative or ethnograph* or "focus group*"))) OR AB (((program* or policy	1,812,106

	or policies or strateg* or scheme* or project* or initiative* or "care package" or training or educat* or pilot or guidance or guideline* or study or pathway or treatment* or promot* or management or "support group" or process* or trial* or intervention*) N5 (evaluat* or effect* or measur* or assess* or experiment* or impact* or feasab* or acceptab* or efficacy or perception* or belief* or uptake or consequence* or attitud* or barrier* or facilit* or motivat* or experience* or implement* or adher* or retention or retain* or reduc* or increas* or improv* or outcome* or cost* or benefit* or interview* or qualitative or ethnograph* or "focus group*")))	
S37	S35 OR S36	6,735
S36	TI "criminal justice" OR AB "criminal justice"	3,845
S35	TI ((probationer* or parolee* or ((repeat* or ex or re or revolving door or habitual or multiple or former* or previously*) N1 (offen* or convict* or prisoner* or imprison* or incarcerat* or criminal*)) or (former N3 inmate*) or ((community or probation* or parole* or reintegrat*) N4 (prison* or offender* or criminal* or convict* or inmate*)) or ((individuals or men or women) N2 (probation or parole)) or ((reintegrate* or reent* or return*) N3 community))) OR AB ((probationer* or parolee* or ((repeat* or ex or re or revolving door or habitual or multiple or former* or previously*) N1 (offen* or convict* or prisoner* or imprison* or incarcerat* or criminal*)) or (former N3 inmate*) or ((community or probation* or parole* or reintegrat*) N4 (prison* or offender* or criminal* or convict* or inmate*)) or ((individuals or men or women) N2	3,325

	(probation or parole)) or ((reintegrate* or reent* or return*) N3 community)))	
S34	S30 OR S31 OR S32 OR S33	14,314
S33	TI (((hous* or home* or accommodat* or shelter) N3 (insecur* or instability or unstable or stability))) OR AB (((hous* or home* or accommodat* or shelter) N3 (insecur* or instability or unstable or stability)))	2,684
S32	TI homeless* OR AB homeless*	10,454
S31	(MH "Homeless Persons")	6,580
S30	TI ((severe and multiple disadvantage*)) OR AB ((severe and multiple disadvantage*))	22
S29	S19 OR S20 OR S21 OR S22 OR S23 OR S24 OR S25 OR S26 OR S27 OR S28	191,033
S28	TI soda OR AB soda	1,512
S27	(MH "Sweetened Beverages")	933
S26	(MH "Carbonated Beverages")	2,794
S25	TI (((sugary or fizzy or carbonated or soft) N1 drink*)) OR AB (((sugary or fizzy or carbonated or soft) N1 drink*))	2,558
S24	TI (((processed or acidic) N1 food*)) OR AB (((processed or acidic) N1 food*))	2,072
S23	(MH "Diet")	63,635
S22	(MH "Dietary Sucrose")	5,119
S21	TI "sugary food*" OR AB "sugary food*"	193
S20	TI diet* OR AB diet*	155,131

S19	TI (((sugar or sucrose or fructose or glucose) N2 (intake or consum*))) OR AB (((sugar or sucrose or fructose or glucose) N2 (intake or consum*)))	5,433
S18	S6 OR S7 OR S8 OR S9 OR S10 OR S11 OR S12 OR S13 OR S14 OR S15 OR S16 OR S17	326,783
S17	TI ((drug N1 (habit or tak* or hard or illicit or inject*))) OR AB ((drug N1 (habit or tak* or hard or illicit or inject*)))	16,744
S16	TI (((alcohol or drug* or substance*) N2 (misuse* or abuse* or use* or addict* or dependenc* or issue* or problem*))) OR AB (((alcohol or drug* or substance*) N2 (misuse* or abuse* or use* or addict* or dependenc* or issue* or problem*)))	132,015
S15	(MH "Behavior, Addictive")	7,159
S14	(MH "Substance Abusers+")	9,952
S13	(MH "Substance Use Disorders+")	182,899
S12	TI "street drink*" OR AB "street drink*"	6
S11	TI ((alcoholic* N3 (person or people or adult or parent* or family))) OR AB ((alcoholic* N3 (person or people or adult or parent* or family)))	429
S10	(MH "Alcoholics")	730
S9	(MH "Alcoholism")	17,654
S8	(MH "Alcohol Drinking")	33,696
S7	TI ((smoking or cigarette* or tobacco)) OR AB ((smoking or cigarette* or tobacco))	114,101

S6	(MH "Smoking")	65,404
S5	S1 OR S2 OR S3 OR S4	51,836
S4	TI (((dental or oral or tooth or teeth or mouth) N3 (health or care or hygiene or rehabilitation))) OR AB (((dental or oral or tooth or teeth or mouth) N3 (health or care or hygiene or rehabilitation)))	33,755
S3	(MH "Dental Health Services+")	20,382
S2	(MH "Oral Health")	14,782
S1	(MH "Oral Hygiene")	6,534

Scopus – search run on 6th February 2023

2846 results

(TITLE-ABS-KEY ((program* OR policy OR policies OR strateg* OR scheme* OR project* OR initiative* OR "care package" OR training OR educat* OR pilot OR guidance OR guideline* OR study OR pathway OR treatment* OR promot* OR management OR "support group" OR process* OR trial* OR intervention*) W/5 (evaluat* OR effect* OR measur* OR assess* OR experiment* OR impact* OR feasab* OR acceptab* OR efficacy OR perception* OR belief* OR uptake OR consequence* OR attitud* OR barrier* OR facilit* OR motivat* OR experience* OR implement* OR adher* OR retention OR retain* OR reduc* OR increas* OR improv* OR outcome* OR cost* OR benefit* OR interview* OR qualitative OR ethnograph* OR "focus group*") OR "housing first" OR (outcome* W/5 evaluat*))) AND ((TITLE-ABS-KEY ((severe AND multiple AND disadvantage*) OR homeless* OR ((hous* OR home* OR accommodat* OR shelter) W/3 (insecur* OR instability OR unstable OR stability)))) OR ((TITLE-ABS-KEY (probationer* OR parolee* OR "criminal justice")) OR (TITLE-ABS-KEY (former W/3 inmate*)) OR (TITLE-ABS-KEY ((community OR probation* OR parole* OR reintegrat*) W/4 (prison* OR offender* OR criminal* OR convict* OR inmate*))) OR (TITLE-ABS-KEY ((individuals OR men OR women) W/2 (probation OR parole))) OR (TITLE-ABS-KEY ((reintegrate* OR reent* OR return*) W/3 community)) OR (TITLE-ABS-KEY ((repeat* OR ex OR re OR "revolving door" OR habitual OR multiple OR former* OR previously*) W/1 (offen* OR convict* OR prisoner* OR imprison* OR incarcerat* OR criminal*)))))) AND ((TITLE-ABS-KEY ((dental OR oral OR tooth OR teeth OR mouth) W/3 (health OR care OR hygiene OR rehabilitation))) OR (TITLE-ABS-KEY (smoking OR cigarette* OR tobacco)) OR (TITLE-ABS-KEY ("alcohol drinking" OR alcoholism OR (alcoholic* W/3 (person* OR people OR adult OR parent* OR family)) OR "street drink")) OR (TITLE-ABS-KEY ((alcohol OR drug* OR substance*) W/2 (misuse* OR abuse* OR use* OR addict* OR depende

nc* OR issue* OR problem OR disorder*) OR (drug W/1 (habit OR tak* OR hard OR illicit OR inject*)))) OR (TITLE-ABS-KEY ((sugar OR sucrose OR fructose OR glucose) W/2 (intake OR consum*) OR diet* OR "sugary food*" OR ((processed OR acidic) W/1 food*) OR ((sugary OR fizzy OR carbonated OR soft OR sweetened) W/1 (drink* OR beverage*)) OR soda)) AND NOT INDEX (medline))
