# Supplementary material for: Factors influencing implementation and sustainability of interventions to improve oral health and related health behaviours in adults experiencing severe and multiple disadvantage: a mixed-methods systematic review
Source: BMJ Open. 2024 Jan 12;14(1):e080160. doi: 10.1136/bmjopen-2023-080160 (PMC10806606; doi:10.1136/bmjopen-2023-080160)
Supplement: Author's manuscript [file bmjopen-2023-080160.draft_revisions.pdf]

BMJ Open is committed to open peer review. As part of this commitment we make the peer review history of every article we publish publicly available.

When an article is published we post the peer reviewers' comments and the authors' responses online. We also post the versions of the paper that were used during peer review. These are the versions that the peer review comments apply to.

The versions of the paper that follow are the versions that were submitted during the peer review process. They are not the versions of record or the final published versions. They should not be cited or distributed as the published version of this manuscript.

BMJ Open is an open access journal and the full, final, typeset and author-corrected version of record of the manuscript is available on our site with no access controls, subscription charges or pay-per-view fees (http://bmjopen.bmj.com).

If you have any questions on BMJ Open's open peer review process please email info.bmjopen@bmj.com

BMJ Open

Factors influencing implementation and sustainability of interventions to improve oral health and related health behaviours in adults experiencing severe and multiple disadvantage: A mixed-methods systematic review

Journal:	*BMJ Open*
Manuscript ID	bmjopen-2023-080160
Article Type:	Original research
Date Submitted by the Author:	22-Sep-2023

Complete List of Authors:	John , Deepti; Newcastle University Population Health Sciences Institute<br>Adams, Emma; Newcastle University Population Health Sciences Institute<br>McGowan, Laura; Newcastle University Population Health Sciences Institute<br>Joyes, Emma; Newcastle University Population Health Sciences Institute<br>Richmond, Catherine; Newcastle University Population Health Sciences Institute<br>Beyer, Fiona ; Newcastle University Population Health Sciences Institute, Evidence Synthesis Group and Innovation Observatory<br>Landes, David; NHS England and NHS Improvement North East and Yorkshire<br>Watt, Richard; University College London, Epidemiology and Public Health; King's College London, King's College Dental Institute<br>Sniehotta, Falko; Newcastle University Population Health Sciences Institute, NIHR Policy Research Unit Behavioural Science; Heidelberg University Medical Faculty Mannheim, Department of Public Health, Social and Preventive Medicine, Centre for Preventive Medicine and Digital Health (CPD)<br>Paisi, Martha; Plymouth University Peninsula Dental School<br>Bambra, Clare; Newcastle University Population Health Sciences Institute<br>Craig, Dawn; Newcastle University Population Health Sciences Institute<br>Kaner, Eileen; Newcastle University Population Health Sciences Institute<br>Ramsay, Sheena; Newcastle University Population Health Sciences Institute

Keywords:	Health Equity, PUBLIC HEALTH, Behavior

SCHOLARONE™
Manuscripts

I, the Submitting Author has the right to grant and does grant on behalf of all authors of the Work (as defined in the below author licence), an exclusive licence and/or a non-exclusive licence for contributions from authors who are: i) UK Crown employees; ii) where BMJ has agreed a CC-BY licence shall apply, and/or iii) in accordance with the terms applicable for US Federal Government officers or employees acting as part of their official duties; on a worldwide, perpetual, irrevocable, royalty-free basis to BMJ Publishing Group Ltd ("BMJ") its licensees and where the relevant Journal is co-owned by BMJ to the co-owners of the Journal, to publish the Work in this journal and any other BMJ products and to exploit all rights, as set out in our licence.

The Submitting Author accepts and understands that any supply made under these terms is made by BMJ to the Submitting Author unless you are acting as an employee on behalf of your employer or a postgraduate student of an affiliated institution which is paying any applicable article publishing charge ("APC") for Open Access articles. Where the Submitting Author wishes to make the Work available on an Open Access basis (and intends to pay the relevant APC), the terms of reuse of such Open Access shall be governed by a Creative Commons licence – details of these licences and which Creative Commons licence will apply to this Work are set out in our licence referred to above.

Other than as permitted in any relevant BMJ Author's Self Archiving Policies, I confirm this Work has not been accepted for publication elsewhere, is not being considered for publication elsewhere and does not duplicate material already published. I confirm all authors consent to publication of this Work and authorise the granting of this licence.

For peer review only

Factors influencing implementation and sustainability of interventions to improve oral health and related health behaviours in adults experiencing severe and multiple disadvantage: A mixed-methods systematic review

Authors
Mrs. Deepti A. John, MDPH - Population Health Sciences Institute, Newcastle University, Newcastle Upon Tyne NE2 4AX, UK
Ms. Emma A. Adams, MSc. - Population Health Sciences Institute, Newcastle University, Newcastle Upon Tyne NE2 4AX, UK
Dr. Laura J. McGowan, PhD - Population Health Sciences Institute, Newcastle University, Newcastle Upon Tyne NE2 4AX, UK
Dr. Emma C. Joyes, PhD - Population Health Sciences Institute, Newcastle University, Newcastle Upon Tyne NE2 4AX, UK
Ms. Catherine Richmond, MA - Population Health Sciences Institute, Newcastle University, Newcastle Upon Tyne NE2 4AX, UK
Dr. Fiona R. Beyer, PhD - Evidence Synthesis Group and Innovation Observatory, Population Health Sciences Institute, Newcastle University, Newcastle Upon Tyne NE2 4AX, UK
Mr. David Landes, BDS - NHS England & Improvement, Newcastle Upon Tyne NE15 8NY, UK
Prof. Richard Watt, PhD - Department of Epidemiology and Public Health, University College London, London WC1E 7HB, UK
Prof. Falko F. Sniehotta, PhD - NIHR Policy Research Unit Behavioural Science, Newcastle University, NE2 4AX And Department of Public Health, Social and Preventive Medicine, Centre for Preventive Medicine and Digital Health (CPD), Medical Faculty Mannheim, Heidelberg University, 68167 Mannheim
Dr. Martha Paisi, PhD - Faculty of Medicine and Dentistry, Peninsula Dental School, University of Plymouth, Plymouth PL4 8AA, UK
Prof. Clare Bambra, PhD - Population Health Sciences Institute, Newcastle University, Newcastle Upon Tyne NE2 4AX, UK
Prof. Dawn Craig, MSc. - Population Health Sciences Institute, Newcastle University, Newcastle Upon Tyne NE2 4AX, UK
Prof. Eileen Kaner, PhD - Population Health Sciences Institute, Newcastle University, Newcastle Upon Tyne NE2 4AX, UK
Prof. Sheena E. Ramsay, PhD - Population Health Sciences Institute, Newcastle University, Newcastle Upon Tyne NE2 4AX, UK

Corresponding Author:
Deepti John
Deepti.john@newcastle.ac.uk
Phone no: +44 7469646623
Population Health Sciences Institute, Newcastle University
The Baddiley-Clark Building, Richardson Road
Newcastle upon Tyne, NE2 4AX
United Kingdom

For peer review only

Abstract

Objectives

Among people experiencing severe and multiple disadvantage (SMD) poor oral health is common and linked to smoking, substance use and high sugar intake. Studies have explored interventions addressing oral health and related behaviours; however, factors related to the implementation of these interventions remains unclear. This mixed-methods systematic review aimed to synthesize evidence on the implementation and sustainability of interventions to improve oral health and related health behaviours among adults experiencing SMD.

Methods

Bibliographic databases (MEDLINE, EMBASE, PsycINFO, CINAHL, EBSCO, Scopus) and grey literature were searched from inception to February 2023. Studies meeting the inclusion criteria were screened and extracted independently by two researchers. Quality appraisal was undertaken, and results were synthesised using narrative and thematic analyses.

Results

Seventeen papers were included (published between 1995-2022). Studies were mostly of moderate quality and included views from SMD groups and service providers. From the qualitative synthesis, most findings were related to aspects such as trust, resources, and motivation levels of SMD groups and service providers. None of the studies reported on diet and none included repeated offending (one of the aspects of SMD). From the quantitative synthesis, no difference was observed in program attendance between the interventions and usual care, although there was some indication of sustained improvements in participation in the intervention group.

Conclusion

This review provides some evidence that trust, adequate resources, and motivation levels are potentially important in implementing interventions to improve oral health and substance use among SMD groups. Further research is needed from high quality studies and focusing on diet in this population.

Strengths and limitations of this study

- Comprehensive search strategy was used to gather evidence in this mixed methods systematic review.

For peer review only

- Consolidated Framework for Implementation Research (CFIR) was used for the data extraction.
- Confidence in the papers were limited due to moderate quality of the papers.
- The included studies were not excluded based on their quality, as they contributed relevant information for this systematic review.

Keywords: multiple disadvantage, homeless, oral health, implementation

INTRODUCTION

Severe and multiple disadvantaged (SMD) populations are individuals who have experienced homelessness, substance use, offending or a combination of all three.[1] They experience disproportionately high levels of poor physical and mental health along with high levels of occupational deprivation,[1] which results in isolation and difficulty in accessing healthcare services.[2] There is also an added burden of stigma that affects their access and engagement.[3]

Among people experiencing SMD, oral health problems have been highlighted as one of the major unmet needs.[4] Aggravated by high levels of smoking, substance and alcohol use and poor diet (high intake of sugar).[4, 5] Elevated tobacco use make them more susceptible to periodontal disease, tooth loss, oral lesions and oral cancer.[5, 6] Research also shows that they do not meet the daily nutritional requirements and have high levels of sugar consumption.[6, 7] Oral health has an overall impact on physical and mental wellbeing.[8] It is, therefore, important to not only address oral health concerns in people experiencing SMD, but also related health behaviours such as smoking, alcohol and substance use, and poor nutrition.[1, 9]

Previous papers focus on intervention design and outcomes, none focus on the implementation approach of these intervention especially in people experiencing SMD.[10-12] Hence, there is a need for evidence on interventions addressing these health challenges, with a specific focus on ways to improve implementation and long-term sustainability of interventions. Frameworks are used to apply a theoretical underpinning to our understanding of why implementation of interventions succeed or fail. The Consolidated Framework for implementation Research (CFIR) composed of five domains was utilised as a theoretical framework to identify the facilitators and barriers that influence implementation. [13, 14] This framework, therefore, assists with bridging the gap between research and practice as well as reducing the challenges of implementing these interventions.[15]

To investigate how we can improve implementation and sustainability, we conducted this systematic review to synthesize various factors such as acceptability, settings, and potential adverse effects of interventions that improve oral health and related health behaviours of adults with SMD.

For peer review only

METHODS

The research protocol was pre-registered and published registered with the Prospective Register of Systematic Reviews (PROSPERO) (reg. no: CRD42020202416).[16, 17] The review was reported according to Preferred Reporting Items for Systematic Reviews and Meta-Analyses (PRISMA) guidelines.[18]

Search strategy

The search strategy (see Appendix A.) was formulated and conducted with an information specialist within the research team. The following electronic databases: MEDLINE (Ovid), EMBASE (Ovid), CINAHL (Ebsco), APA PsycINFO (Ovid) and Scopus were searched for relevant qualitative, quantitative, and mixed method studies from inception to February 2023. Grey literature searches were conducted using Google Incognito and selected charity organization websites such as Fulfilling Lives, Crisis, Groundswell, which were informed by the expertise of the research team. Forward and backward citation search of the included studies were also conducted.

Study selection

The search results were downloaded and deduplicated using EndNote 20.4.1 and the uploaded into Covidence, an online tool for managing the whole systematic review process.[19] Title, abstracts and full texts were independently screened by two reviewers. In the case of a discrepancy, consensus was reached after consultation with a third reviewer. Table 1 presents the inclusion criteria used during screening.

Table 1: *Eligibility Criteria used to select the studies*

	Eligibility Criteria
Population	Adults aged 18 or above, who experience SMD comprising of either homelessness (rough sleeping or other types of insecure accommodation), repeated offending or frequent substance use that co-occurs with homelessness or repeated offending.[17] Perspectives of staff who work with SMD groups and stakeholders such as policy makers and commissioners.
Intervention	Structural, community and individual level interventions.[17]
Outcomes	Views from SMD groups and other stakeholders (policy makers, service providers, voluntary sector etc) about implementation and sustainability of interventions which include acceptability, content, settings, potential harms, uptake, and retention.[17]

For peer review only

Study Design	Qualitative, quantitative, and mixed method studies

Data extraction and quality appraisal

The data extraction and quality assessment for all the included studies were conducted by one reviewer and cross-checked by a second reviewer. Included studies were critically appraised to guide how much confidences could be placed on the findings. Qualitative studies were appraised using the Critical Appraisal Skills Programme (CASP) qualitative checklist.[20] Quantitative studies were appraised using Cochrane's Risk of bias for randomized control trials (RCTs).[21] For cross-sectional studies the National Institutes of Health (NIH) Study Quality Appraisal Tool was used.[22] Qualitative studies were rated as good, moderate or low quality, which was informed by a scoring system: scores 9–10 was high quality, 7.5–9 was moderate quality and <7.5 was low quality.[23] The scoring were informed by the quality checklists. The studies were not excluded based on their quality, as they all contributed data relevant to this review.[24]

Data synthesis

Abstracts and data from the results of included studies were uploaded on to NVivo software (QSR International Pty Ltd., Melbourne, Australia Version 12, Release 1.6.1). Narrative synthesis was undertaken. Deductive codes based on the CFIR framework were used to initially code the findings followed by a three-step inductive synthesis process which involved coding the text, identifying the themes, and creating the subthemes. To maximise thematic yield, data reported in different papers but from the same study were individually coded. The developing themes and subthemes were discussed with the other reviewers and consensus was reached regarding these.

RESULTS

Seventeen articles (twelve individual studies) met the inclusion criteria and were included in this systematic review. Figure 1 presents the PRISMA flowchart for included studies. Table 2 presents the descriptive summaries of the included studies. The papers were published between 1995 and 2022, and were related to interventions targeting oral health,[25-32] substance use,[33-39] smoking and none on diet.

For peer review only

Table 2: *Descriptive summaries of the seventeen included studies, including quality appraisal (High quality/moderate quality/low quality)*

No.	*First Author (Year) and Country*	*Sample Size and Age of the participants*	*Participant Group*	*Intervention Description*	*Type of Research, Data Collection and Analysis*	*Findings of the paper relevant to the review*	*Quality Appraisal/ Risk of Bias*
1.	Beaton et al (2016) *United Kingdom*[25]	N=20, age not mentioned	Health and social care workers	Motivational interviewing to promote oral health among homeless populations ("*Smile4life programme*")	Qualitative - Telephone interviews, framework approach	Familiarity and good relationships between service providers and third sector organizations facilitated implementation whereas lack of resources and interest hindered it	High quality
2.	Beaton et al (2018) *United Kingdom* [26]	N= 9 observation sessions, age not mentioned	Oral healthcare workers such as oral health educators and dental support workers	Motivational interviewing and tailored advice to promote oral health among the homeless population at different settings such as mobile dental units and homeless shelters ("*Smile4Life programme*")	Qualitative- Participant observation, content analysis	Good working relationships between healthcare providers, patients and third sector organizations are important	Moderate quality

For peer review only

No.	*First Author (Year) and Country*	*Sample Size and Age of the participants*	*Participant Group*	*Intervention Description*	*Type of Research, Data Collection and Analysis*	*Findings of the paper relevant to the review*	*Quality Appraisal/ Risk of Bias*
3.	Beaton et al (2021) *United Kingdom* [27]	N= 100, 16- 85 years	Oral health practitioners, third sector organization staff and local authority staff	Motivational interviewing and behavioural change techniques to promote oral health among the homeless *("Smile4Life programme")*	Quantitative- Questionnaire, K-R20, Exploratory factor analysis, multivariate path analysis.	Work practices such as positive attitudes and beliefs of the oral healthcare workers influence implementation	Moderate/ Fair quality
4.	Burnam et al (1995) *United States of America*[34]	N= 276, mean age = 37 years	Homeless individuals with co-occurring substance and mental health issues	Social model of residential and non-residential programs providing integrated substance use and mental health services	Quantitative- Structured interviews, regression analyses	Retention levels were higher in the residential program compared to the non-residential one	Low quality (high Risk of Bias)
5.	Coles et al (2013) *United Kingdom*[29]	N= 14, age not mentioned	Healthcare workers from statutory and non-statutory organizations	A framework that offers tailored oral health advice and signposts to relevant dental services. (*"Something To Smile About"*)	Qualitative- Focus groups, content analysis	Oral health knowledge among the healthcare workers improved but complex needs such as housing, employment etc must be addressed prior to oral health for successful implementation	Moderate quality

For peer review only

No.	*First Author (Year) and Country*	*Sample Size and Age of the participants*	*Participant Group*	*Intervention Description*	*Type of Research, Data Collection and Analysis*	*Findings of the paper relevant to the review*	*Quality Appraisal/ Risk of Bias*
6.	Collins et al (2019) *United States of America*[35]	N= 168, mean age= 47 years	Homeless individuals with alcohol use disorder	Non-abstinence treatment program that involves tracking of alcohol use, discussion of safe drinking practices and goal-oriented tasks. *("Harm Reduction Treatment for Alcohol HaRT-A")*	Quantitative- Questionnaires, content analysis	It was positively viewed by the participants with high levels of retention and satisfaction	Good quality (low ROB)
7.	Doughty et al (2020) *United Kingdom*[31]	Service users – N= 353, age not mentioned Service providers – not stated	Homeless individuals and oral healthcare workers such as dentists, dental nurses, dental technicians etc	Denture service provided by Crisis at Christmas Dental Service and Den-tech to the homeless and vulnerably housed	Qualitative	Communication, timing, resources, and training were considered as areas that needed to be improved	Low quality
8.	Forchuk et al (2022) *Canada*[36]	Service users – N= 58, mean age = 52.5 years Service providers – not stated	Homeless veterans with substance use problems and staff from housing services	Housing provided along with the peer support and harm reduction services to homeless veterans ("*Housing First*")	Qualitative - Interviews and focus groups, Thematic analysis	Stable housing with harm reduction services was well received. Collaboration between mental health and addiction services should	Low quality

For peer review only

No.	*First Author (Year) and Country*	*Sample Size and Age of the participants*	*Participant Group*	*Intervention Description*	*Type of Research, Data Collection and Analysis*	*Findings of the paper relevant to the review*	*Quality Appraisal/ Risk of Bias*
						be considered for future services	
9.	Henderson et al (2004) *United States of America*[37]	Service users – N = 15 Service providers – not mentioned	Homeless veterans with substance/alcohol use and program staff such as healthcare workers and administrative staff	Residential substance use treatment program that focuses on relapse prevention along with education and housing stability for homeless men	Qualitative - Surveys, direct observation and Interviews, not stated	Majority of the participants provided positive feedback. Staffing issues such as training and competing workload were noted as drawbacks to the program	Moderate quality
10.	Neale et al (2014) *United Kingdom*[33]	Service users - N= 30, 23- 62 years Service providers – N= 15, age not mentioned	Homeless individuals with substance use and mentors such as substance use workers, substance use managers and hostel staff	Computer assisted therapies using 20 different psychosocial intervention strategies to identify and reduce substance use based in hostels and homeless shelters *("Breaking Free Online")*	Qualitative- Interviews, Inductive coding and Framework approach	'Program features', 'mentor support', 'participant characteristics' and 'delivery context' were noted as factors that lead to successful delivery.	Moderate quality

For peer review only - http://bmjopen.bmj.com/site/about/guidelines.xhtml

No.	*First Author (Year) and Country*	*Sample Size and Age of the participants*	*Participant Group*	*Intervention Description*	*Type of Research, Data Collection and Analysis*	*Findings of the paper relevant to the review*	*Quality Appraisal/ Risk of Bias*
11.	Paisi et al (2020) *United Kingdom* [32]	Service users – N= 11, 20 -65 years Service providers – N= 11, age not mentioned	Homeless individuals and the dental clinic staff members, support workers and volunteers.	Community dental clinic that provides both regular and emergency treatments.	Qualitative – semi structured interviews, reflective thematic analysis	Flexibility and the relationship between the patient and dental provider were highlighted as important features.	High quality
12.	Pauly et al (2020) *Canada*[38]	N= 14, 29-61 years	Homeless with illicit alcohol use	Non-residential community managed alcohol program which provides harm reduction strategies and peer support *("Canadian Managed Alcohol Program Study")*	Qualitative- Semi-structured interviews, inductive coding and constant comparative analysis	Peer led program was successful as it facilitates capacity building, engagement, and empowerment	Moderate quality
13.	Pratt et al (2019) *United States of America*[40]	N= 40, 29-69 years	Homeless with smoking and alcohol use	Nicotine replacement therapy and motivational interviewing/cognitive behavioural therapy to reduce smoking and	Qualitative- Interviews, social constructivist approach to grounded theory	Social (peer groups) and environmental (housing etc) factors impact cessation in homeless smokers	High quality

No.	*First Author (Year) and Country*	*Sample Size and Age of the participants*	*Participant Group*	*Intervention Description*	*Type of Research, Data Collection and Analysis*	*Findings of the paper relevant to the review*	*Quality Appraisal/ Risk of Bias*
				alcohol use among the homeless *("Power To Quit 2")*			
14.	Pratt et al (2022) *United States of America* [41]	N= 40, 29-70 years	Homeless with smoking and alcohol use	Nicotine replacement therapy and motivational interviewing/cognitive behavioural therapy to reduce smoking and alcohol use among the homeless *("Power To Quit 2")*	Qualitative – Semi structured interviews, social constructivist approach to grounded theory	Social pressure and shelter environment impact the intervention but the integrated treatment along with emotional support from the staff make it beneficial.	High quality
15.	Rash et al (2017) *United States of America*[39]	N= 355, mean age = 37 years	Homeless with substance use	Behavioural intervention contingency management with the use of incentives such as vouchers and prizes delivered at local community clinics	Quantitative- Adaptation of the Service Utilization Form, Multivariate analysis of variance.	Retention was higher in groups that accessed the intervention compared to the standard arm of care	Moderate/fair quality
16.	Rodriguez et al (2019) *United Kingdom*[28]	Service users - N= 13, 18- 22 years	Young homeless people and NGO practitioners	Pedagogical workshops about oral health, mental health, substance misuse, diet etc to increase	Qualitative- Unstructured interviews and	Involvement of young people in co-designing an intervention facilitates engagement,	High quality

For peer review only

No.	*First Author (Year) and Country*	*Sample Size and Age of the participants*	*Participant Group*	*Intervention Description*	*Type of Research, Data Collection and Analysis*	*Findings of the paper relevant to the review*	*Quality Appraisal/ Risk of Bias*
		Service providers – N= 5, age not mentioned		engagement and awareness	workshops, content analysis	trust building and increases health literacy	
17.	Stormon et al (2018) *Australia*[30]	N= 76, 41-60 years Feedback – N=24	Disadvantaged adults (clients of community organizations that utilize housing, employment and food services)	Facilitated access pathway between homeless organizations and public dental services. Improving oral health by assessing dental needs, offering dental advice and dental appointments	Quantitative- Questionnaire, Descriptive analysis, and Framework approach	Positive feedback by participants facilitated by the environment, clinical staff and flexibility. Attendance rates varied across the site but was generally high.	Fair/ moderate quality

For peer review only

SMD groups in the studies found in this review included young adults, single mothers, veterans, and adults with co-occurring conditions of severe mental illness. Based on the information reported in the studies, most of the interventions were focused on adults who were experiencing homelessness and substance use issues,[33-40] but did not explicitly report on whether they included those who had repeated involvement with the criminal justice system.

Quality appraisal

Of the twelve articles reporting qualitative findings, two were low quality due to lack of detailed findings and methodology not being reported adequately,[31, 36] five moderate quality,[26, 29, 33, 37, 38] due to reporting bias and five high quality.[25, 28, 32, 40, 41] The risk of bias was assessed for the five articles reporting quantitative findings; among the two RCTs: one had a high risk of bias because of attrition and reporting bias,[34] and the other article had a low risk of bias,[35] the remaining three cross-sectional studies were of moderate quality.[27, 30, 39]

Synthesis of qualitative findings

Table 3 presents the themes, subthemes, codes, and quotes from individuals experiencing SMD and frontline staff and stakeholders.

For peer review only

Table 3: *Themes and subthemes from qualitative synthesis of findings along with the relevant codes and quotes*

Themes	Subthemes	Codes	Quote(s) – people with SMD; or frontline staff
Intervention settings	Physical settings	Housing stability, privacy, confidentiality	*"This is kind of a stressful situation. People are homeless, being at the bottom of their luck, and—boom—and everything. So this is stress. What do you do? You drink, and you smoke, and that's all that you can do, walking around here all day. Do you understand?"(person with SMD)* [40]<br><br>*"But at the same time the addictions piece, especially in terms of stability, I've noticed a lot of the guys that because they are stable in our home, they may make the choice more often to say 'I don't feel like drinking tonight,' so they don't. They don't have to get intoxicated to go to sleep in a shelter on a mat, they can choose not to drink and sometimes they do make that choice not to drink and just watch TV for the evening."(frontline staff)* [36]<br><br>*"If you went in and tried to do anything, people were behind you, over your shoulder, 'what are you doing there' And, you know, I didn't what to discuss with people what I was doing, because they'd take the mick"(person with SMD)* [33]
	Psychological aspects of settings	Communication, trust building, familiarity, mentorship, community, peer pressure, guidance, support, safe space	*"It's not just about dental treatment, I think for a lot of people there is the fear of the dentist because when they do go, it's because they need work done and they're in pain, therefore they associate pain with the dentist."(frontline staff)* [29]

For peer review only

Themes	Subthemes	Codes	Quote(s) – people with SMD; or frontline staff
			"She's not somebody that normally expresses much in a group, she's quite a private person, so I thought it took quite a lot for her to open up, to trust, but I also appreciate the fact that she felt she was in a really good space that she could share that experience with the others and I felt that was really valuable for the rest of the group to hear that. I think this activity [the workshops] encourages people to talk about their own experiences"(frontline staff)[28]<br><br>*"Yes. A lot better off because… I'm not like, like when I'm here and I'm here with people that are drinking on programs like this and stuff like that I've noticed we're all on the same level. We don't care about the issues or problems, we just, you know, pitch together and do what we gotta do to get ourselves fixed and then from there if we can help other people, and people help other people…"(person with SMD)* [38]<br><br>*"If there is nobody there and you're just left to get on with it, it's quite easy to skip things…I will just put that answer down, you know. But then when you know somebody is there and they are there for that specific reason, then it's a lot easier to go through with things."(person with SMD)* [33]
	Accessibility	Point of contact, space, geography	*"We were put in the medical room along the corridor from the office, but there was no opportunity for practitioner 4 to approach any of the*

For peer review only

Themes	Subthemes	Codes	Quote(s) – people with SMD; or frontline staff
			residents. We only saw service users if they specifically wanted to talk about their oral health or if they had walker past the room and wanted to see who we were." (frontline staff) [26]<br><br>*"I'm from a very rural area, and we don't really have any homelessness centres." (person with SMD)* [25]
Intervention delivery	Improved Awareness	Understandable, ideas, learning from one and another	*"Take things that people say and take it on board, and everything's a learning curve, you learn things all the time... And I'd recommend that to anybody else who is homeless, just listen to other people, take on board what they've got to say, and accept the help that's around you like the group activity [the workshops]".(person with SMD)* [28]<br><br>*"Especially when it was to do with what alcohol can do and what substances can do, I don't think they realized how that affects their oral health, their ears pricked up when you said that" (frontline staff)* [29]
	Resources	Workloads, stress, competing needs, volunteers, equipment, funds	*"I think he [client] felt that maybe I would have to sit with him again and, I don't know, maybe I should have sat him down and had a talk with him and I just haven't been able to"(frontline staff)* [33]<br><br>*"You feel like you're spinning so many plates, that you just can't possibly keep them all up in the air" (frontline staff)* [25]

For peer review only

Themes	Subthemes	Codes	Quote(s) – people with SMD; or frontline staff
			"we need to attract funding … it's very difficult to encourage NHS England to commission outside of their routine, the existing contract doesn't favour patients with high treatment needs so we would need them to step outside of their comfort zone and commission something slightly different to what they're used to"(frontline staff) [32]
	Perceived risks while working with a vulnerable population	Safety, unpredictable, inappropriate behavior, challenges, relevant experience, confident, challenging behaviors	*"Practitioner 1 is confident and appears quite fearless, putting up with language/behaviour that would not be tolerated in a normal clinic." (researcher observation notes of frontline staff)* [26]<br><br>*"Initially we were thinking 'oh we need to make sure that we're not alone in the surgery at any point', and we had a panic alarm and things, we still have all that in place, but it's actually been fine."(frontline staff)* [32]
Ways to enhance engagement and participation	Interest and motivation	Complexity, fears, initiative, specific and complex needs, mixed opinions	*"[Mentor] came in and said 'I'm going home, have you done much?' And I said, 'I couldn't get back on, you know'. And she just took it [the laptop]. I don't know if she was fed up with me or whatever, but she never spoke about it again and I never mentioned it again."(person with SMD)* [33]<br><br>*"The oral health team do not seem bothered to recruit any patients, even if that means sitting waiting with nothing to do—the feeling seems to be*

For peer review only

Themes	Subthemes	Codes	Quote(s) – people with SMD; or frontline staff
			that if a patient wants to be seen then they will come to the MDU."(researcher observation notes of frontline staff) [26]<br><br>*"My goal is to quit within a month or two months. I talked to a couple of people. 'It ain't going to happen.' I said, 'well if you set your mind to certain things, you can do this."(person with SMD)* [40]<br><br>*"I think it's good. It made me feel like I had something to do or like I had a purpose. You know what I mean, not a purpose but it wasn't like the homeless"*[41]
	Adapt to specific circumstances	Context, tailored to the needs of the individual, personalized care	*"People getting through the door, they might not have a roof, might not have any money, might have major drug and alcohol issues, might be threatened with violence, the last thing they want to talk about is their teeth."(frontline staff)* [29]<br><br>*"'we call these people chaotic and that's a bit judgmental, they are actually setting priorities, they've got so much going on in their lives that it [oral health] just falls of their list of priorities, they're saying 'it's my priority to find somewhere to sleep tonight' … The time that you catch people' was therefore identified as 'really important'."(frontline staff)* [32]

For peer review only

Themes	Subthemes	Codes	Quote(s) – people with SMD; or frontline staff
	Constant support	Long term care, advice, support	*"About three or four in the morning and I feel like upset then…I can come down and use the program, which is quite good because that way I can put stuff that is all jumbled up in my head down in a way that makes sense and it kind of makes you see that things aren't quite so bad as they seem."(person with SMD)* [33]<br><br>*"At the stage of having goals, an action plan and were working through that . . . but for some homeless people who are nowhere ready, you can make an average of seven appointments before they will turn up once, it's just where your client is at."(frontline staff)* [29]

For peer review only

Synthesis of twelve papers with qualitative findings,[25, 26, 28, 29, 31-33, 36-38, 40, 41] identified three overarching themes in relation to the aims of this review. The three themes are: 1) Intervention settings, 2) Intervention delivery and 3) ways to enhance engagement and participation.

Theme 1: Intervention settings

Eleven papers identified issues related to the settings of interventions which can play a role in the delivery of interventions targeting oral health, substance use and smoking.[25-28, 32, 33, 36-38, 40, 41]

Physical settings

Physical settings involved the environment in which the intervention took place. The wider physical environment has been found to have an impact on the intervention experience,[41] with privacy being the key factor for improving physical settings.[32, 33, 41] Communal homeless shelters and busy teaching hospitals lack the space and privacy to deliver interventions involving discussions about difficult and sensitive topics.[32, 33, 41] Contrastingly, stable housing with the necessary privacy allowed people experiencing SMD to focus on their recovery journey, whilst also creating a space in which residents could spend time away from peers who were sometimes perceived as having a negative peer group influence.[36, 40]

Psychological aspects of settings

Psychological aspects related to the less visible parts of the interventions were identified across ten papers.[25, 26, 28, 29, 32, 33, 37, 38, 40, 41] Firstly, it was reported that relationships between people experiencing SMD and service providers played a vital part in the delivery of interventions. Through good communication,[26, 28, 32, 41] trust building,[28, 29, 32, 41] familiarity of working with a vulnerable population,[25, 32] and mentorship,[33, 37] interventions were able to form a 'safe and respectable environment'.[28, 32, 37, 41] Secondly, papers discussed the importance of peer support as a way of increasing the effectiveness of interventions.[32, 37, 38]There were also reports of the impact negative peer influence could have on the recovery process. For example, smoking and drinking were linked to socializing with others, which could increase the urge to smoke or drink.[40, 41]

Accessibility

Accessibility of interventions was one of the factors found to be important related to implementation of interventions among people experiencing SMD.[25, 26, 32, 41] Firstly, accessible and spacious meeting points within the services were reported to help with their participation in the intervention, especially in the case of oral health interventions that were delivered either in a community setup (open space) or a mobile dental van .[26]Secondly, geographical proximity could act

as a barrier as rural and remote areas lack the facilities and resources, which could influence the access of people experiencing SMD.[25, 32] Lastly, it was reported that access could become an issue when service users move to more stable housing as weather conditions, distance, work and other appointments tend to make it challenging to attend the intervention sessions.[41]

Theme 2: Intervention delivery

Nine papers discussed aspects such as information availability, resources and perceived risks of working with a vulnerable population that could be important for roll out and delivery of interventions addressing oral health, smoking and substance use.[25, 26, 28, 29, 31-33, 37, 41]

Improved Awareness

Awareness and information availability were discussed in papers focusing on improving oral health, smoking and alcohol use.[28, 29, 32, 41] Sharing information between service providers and SMD groups was identified as an important issue across the papers as it created opportunities to promote involvement and behavior change.[28, 29, 41] It was reported that easily understandable information encouraged people experiencing SMD to view healthier behaviors as important (e.g. tooth brushing) and helped to signpost them to necessary services.[28, 41] Clear and simple explanations of treatment options available was seen to help them in decision-making.[32] Service providers also felt that they learned more about healthy behaviors and were able to pass their newly gained knowledge to their clients.[29]

Resources

Five papers discussed the importance of having necessary resources to enable interventions to run efficiently and effectively.[25, 31-33, 37] The majority of these highlighted the importance of distribution of workloads among staff because of difficulties in implementing interventions with competing duties and work within the organizations.[25, 33, 37] Funding and resources such as volunteers and materials were identified in oral health interventions as an important issue that impacts implementation and long-term sustainability.[31, 32]

Perceived risks working with a vulnerable population

Papers reported on the perceived risks of delivering interventions to vulnerable populations as challenging at times by service providers.[25, 26, 32] There were concerns about safety of service providers while interacting with clients who were seen to be “unpredictable”. The need for training and being better equipped to work in this environment and setting boundaries between service providers and clients was repeatedly mentioned by service providers.[25, 26, 32, 37] The papers also

For peer review only

highlighted the importance of training opportunities that provide service providers with the necessary skills to handle volatile and difficult situations.[25, 37]

Theme 3: Ways to enhance participation and engagement

Ten papers identified factors such as interest and motivation levels, adaptability, and long-term support that could help to improve outcomes and create sustainable interventions by enhancing engagement and participation.[25, 26, 28, 29, 31-33, 37, 40, 41]

Interest and motivation

Nine papers highlighted that the interest and motivation levels of both staff supporting SMD groups and people experiencing SMD play an important role in the implementation of interventions. Disinterest was sometimes observed amongst service providers, due to concerns about the complexity of delivering the intervention,[25, 29, 31] lack of engagement with third sector organizations,[26] poor uptake of the intervention by the target populations,[25, 29, 31] and preconceived notions of improper behaviour by SMD groups.[41] Interestingly, interventions were met with similar feelings of indifference by people experiencing SMD if the intervention did not address their specific and complex needs such as housing and financial problems.[25, 26, 32] Two papers on oral health interventions found that younger adults and families with children were more eager to engage compared to single men.[28, 29] Papers discussing the same smoking intervention illustrated that an awareness of health benefits and risks played a part in motivating people in engaging with the intervention.[40, 41]

Adapting to specific circumstances

Adaptability of interventions was noted as an essential feature among four papers.[29, 32, 33, 40] Tailoring the interventions to address their specific needs at the time such as housing and employment was noted to increase participation and better outcomes.[29, 40] Service users of a community dental service also suggested flexible and longer dental appointments would be helpful and in the long-term these adaptions would help reduce missed appointments.[32] Another paper reported that people experiencing SMD were keen to have more face to face interactions rather than digital, which highlights the drive to more personalized care.[33]

Long term support

Four papers identified sustained and long-term support as a factor that could contribute towards better intervention outcomes.[29, 32, 33, 37] Service providers expressed a need for interventions which allowed people experiencing SMD to continue with services/programmes despite missing

For peer review only

appointments or not completing treatment within the required delivery timeframe especially because of the transitionary nature of SMD groups.[32, 33] Similarly, for a substance reduction intervention, a preference for a long-term intervention, that allowed and supported them to gradually integrate into their new stage of their lives.[33, 37] Two papers on oral health interventions suggested that drop-in services offered flexibility in seeking advice or seeing a practitioner and helped to reduce anxiety surrounding accessing treatment for dental health.[29, 32]

Synthesis of quantitative findings related to retention and implementation

Four papers reported quantitative findings on retention and program attendance,[30, 34, 35, 39] as indicators of uptake and sustained implementation of interventions.

Three papers on substance use interventions reported high levels of retention in their intervention groups.[34, 35, 39] One paper found that retention was not significantly associated to housing but to the type of treatment received at the intervention (e.g. contingency management vs. standard care).[39] Contrastingly, another intervention delivered in both residential and non-residential settings reported that retention was greater in the residential program (24/7 program) compared to the non-residential program (5 days/ week from 1 to 9pm).[34]

There was no difference in the attendance levels in the studies related to substance use interventions.[34, 35] The attendance level for an oral health promotion intervention delivered in community settings was high (85%), however it varied across community centers and was dependent on timing of appointment and dental treatments offered. More non-attendance seen for afternoon appointments and complex dental treatments (e.g. surgical and prosthodontic treatments).[30]

Additionally, workplace beliefs and practices amongst service providers such as knowledge, intention and goals, were reported to influence implementation behaviors.[27]

DISCUSSION

This review synthesized different factors that could influence the implementation and sustainability of interventions related to improving oral health and related health behaviors of people experiencing SMD. Evidence suggested that psychological aspects of intervention settings such as building trust and communication form an integral part in the creating a safe environment and that these are just as essential as the structural components of settings such as physical environment. Review findings further suggest that adequate staff capacity, funding and equipment would ease the delivery of interventions by reducing the immense pressure faced by service providers supporting the interventions. It was also suggested that implementation is dependent on the interest and

For peer review only

motivation of not only people experiencing SMD but also on that of service providers in delivering difficult and complex interventions.

Most of the included studies were related to oral health and substance use (drug and alcohol). There was a lack of evidence on diet and smoking interventions among this population. Previous evidence has shown that tobacco use and poor diet, often due to limited choice available while experiencing homelessness and related disadvantages, result in a range of adverse short term (nutritional deficiencies) and long term health outcomes (cancer, diabetes, heart disease).[42-44] Food insecurity is often linked to elevated tobacco use, mental health issues and an increased risk of substance misuse.[45-47]

While most of the papers mainly focused on the perspectives of people experiencing SMD, the limited data from service providers brought to light some of the challenges faced during implementation. This supports the notion that intervention implementation needs the co-ordination and collective effort of everyone involved. All the interventions included were designed focussing on service provision,[25-28, 30-35, 37-41, 48] except for one study which focussed on an training intervention for service providers [29].Limited evidence was available on the long-term sustainability of interventions, which highlights another evidence gap that needs to be addressed.

Our review findings suggest that the retention in interventions may depend on the type of treatment offered, which at times can be influenced by the availability of housing provision. Timing and type of treatment may also influence attendance rates; for instance, morning appointments might be more beneficial, especially for individuals struggling with alcohol addiction, as they may be less intoxicated compared to later in the day. The findings we have are very limited regarding retention and attendance, more effort needs to be taken to understand how to improve reach and retention among SMD groups so that they can access and use the interventions efficiently.

A systematic review on access to dental care among individual experiencing homelessness in the UK identified similar findings around awareness, accessibility and organizational issues (lack of financial resources and collaboration between sectors) being important toward implementation.[49] This was also similarly identified in another review on smoking cessation among homeless populations in high-income countries.[50] The importance of continued engagement in services was highlighted in a review on substance use support for young people (ages 12 to 24) experiencing homelessness, which was also reflected in our findings.[51] Existing literature on interventions targeting health conditions such as HIV and Hepatitis C in this population have shown that improved health outcomes are linked to increased awareness, establishment of positive relationships with service providers and integrated treatment involving other health behaviours.[52-54]

For peer review only

Some findings from our review on aspects related to intervention settings, and intervention delivery aligned with CFIR constructs of Inner and Outer settings domains.[14] Subthemes in our findings on ways to enhance engagement, aligns with both Individuals and Implementation process domains.[14] The use of CFIR framework helps us understand the impact of intervention settings, delivery methods and engagement on the implementation process. It also provides a comprehensive approach for guiding the development of interventions targeting SMD groups and improving their efficacy in practical settings.

Strengths and Limitations

This systematic review is novel in that it assesses the implementation and sustainability of interventions on oral health together with co-occurring and related health behaviors in people experiencing SMD. It addresses an evidence gap on interventions targeting these health challenges and identifies ways to overcome implementation issues faced by these specific interventions. Another strength of this review lies in its comprehensive search strategy and use of a published tool (i.e. CFIR) to make sense of the results. It also highlights gaps in the evidence base on interventions related to diet, as well as studies that include repeat offenders. However, the confidence in the evidence from this review is limited as most of the papers were of moderate quality. Studies lacked detailed data collection methods and standardized evaluations which influenced their quality. Another limitation of this work is that intersectionality was considered explicitly during the analysis of the data. Furthermore, the findings may not be generalizable to all contexts since the included papers were from high-income countries.

Implications

These findings offer valuable insights for enhancing existing interventions by paying attention to settings, delivery, and engagement opportunities. Evidence from this review points to the need for additional research on interventions targeting smoking and diet. These areas hold significant value due to their direct links with general and oral health. It is also important for interventions to address not only individual behaviours but also overlapping behaviours of substance use, smoking and poor diet. This could help reduce the strain on resources and improve engagement. Furthermore, higher quality research that focuses more on sustainability and intersectionality is warranted to further investigate and refine interventions focused on SMD groups.

For peer review only

Author Contributions

Conceptualization: SER, EK, EAA, CB, RGW, ECJ, LJM, FB, DC, DL, MP, FFS; methodology: SER, DAJ, LJM, EAA, ECJ, CR, EK, DC, FB; draft preparation: SER, DAJ, EAA; writing - review and editing all; funding acquisition: SER, EK, FB, CB, RGW, DC, DL, MP, FFS.

Ethical approval

None sought.

Data Availability Statement

No data are available.

Patient and public involvement

Patients and/or the public were not involved in the design, or conduct, or reporting, or dissemination plans of this research.

Funding Statement

This project is funded by the National Institute for Health and Care Research (NIHR) Policy Research Programme (NIHR200415). EAA was supported by the National Institute for Health and Care Research (NIHR) School for Public Health Research (SPHR) Pre-doctoral Fellowship, Grant Reference Number PD-SPH-2015. EK is supported by an NIHR Senior Investigator award and directs the NIHR funded Applied Research Collaboration North East and North Cumbria. The views expressed are those of the author(s) and not necessarily those of NIHR or the Department of Health & Social Care.

Conflicts of Interest

The authors declare no conflict of interest. The funders had no role in the design of the study; in the collection, analyses, or interpretation of data; in the writing of the manuscript, or in the decision to publish the results.

Word Count

3329 words

References:

1. Bramley G, Fitzpatrick S, Edwards J, Ford D, Johnsen S, Sosenko F, et al. Hard edges: mapping severe and multiple disadvantage in England. 2015.

For peer review only

2. Sandu RD. Defining severe and multiple disadvantage from the inside: Perspectives of young people and of their support workers. Journal of Community Psychology. 2021; 49:1470-86.
3. Rae BE, Rees S. The perceptions of homeless people regarding their healthcare needs and experiences of receiving health care. Journal of Advanced Nursing. 2015; 71:2096-107.
4. Conte M, Broder HL, Jenkins G, Reed R, Janal MN. Oral Health, Related Behaviors and Oral Health Impacts Among Homeless Adults. J Public Health Dent. 2006; 66:276-8.
5. Hertzberg D, Boobis S. Unhealthy State of Homelessness 2022: Findings from the Homeless Health Needs Audit. 2022.
6. Groundswell. Healthy Mouths: A peer-led health audit on the oral health of people experiencing homelessness. London Groundswell2017.
7. Evans NS, Dowler EA. Food, health and eating among single homeless and marginalized people in London. Journal of Human Nutrition and Dietetics. 1999; 12:179-99.
8. Sheiham A. Oral health, general health and quality of life. SciELO Public Health; 2005. p. 644-.
9. Wiecha JL, Dwyer JT, Dunn-Strohecker M. Nutrition and health services needs among the homeless. Public Health Rep. 1991; 106:364-74.
10. Beaton L, Humphris G, Rodriguez A, Freeman R. Community-based oral health interventions for people experiencing homelessness: a scoping review. Community Dent Health. 2020; 37:150-60.
11. Hwang SW, Tolomiczenko G, Kouyoumdjian FG, Garner RE. Interventions to improve the health of the homeless: a systematic review. American journal of preventive medicine. 2005; 29:311-. e75.
12. Speirs V, Johnson M, Jirojwong S. A systematic review of interventions for homeless women. Journal of Clinical Nursing. 2013; 22:1080-93.
13. Damschroder LJ, Aron DC, Keith RE, Kirsh SR, Alexander JA, Lowery JC. Fostering implementation of health services research findings into practice: a consolidated framework for advancing implementation science. Implementation Science. 2009; 4:50.
14. Damschroder LJ, Reardon CM, Widerquist MAO, Lowery J. The updated Consolidated Framework for Implementation Research based on user feedback. Implementation Science. 2022; 17:75.
15. Shelton RC, Cooper BR, Stirman SW. The Sustainability of Evidence-Based Interventions and Practices in Public Health and Health Care. Annual Review of Public Health. 2018; 39:55-76.
16. McGowan L, Joyes E, Adams E, Richmond C, Shabaninejad H, Beyer F, et al. Systematic review of oral health and related health behaviour interventions in adults with severe and multiple disadvantages. PROSPERO. 2020.
17. McGowan LJ, Joyes EC, Adams EA, Coyte A, Gavin R, Richmond C, et al. Investigating the Effectiveness and Acceptability of Oral Health and Related Health Behaviour Interventions in Adults with Severe and Multiple Disadvantage: Protocol for a Mixed-Methods Systematic Review. Int J Environ Res Public Health. 2021; 18.
18. Page MJ, Moher D, Bossuyt PM, Boutron I, Hoffmann TC, Mulrow CD, et al. PRISMA 2020 explanation and elaboration: updated guidance and exemplars for reporting systematic reviews. BMJ. 2021; 372:n160.
19. Covidence. Covidence Systematic Review Software.
20. Programme. CAS. CASP Qualitative Checklist. 2022 [13/03/2023]; Available from: https://casp-uk.net/casp-tools-checklists.

21. Higgins JP, Thomas J, Chandler J, Cumpston M, Li T, Page MJ, et al. Cochrane handbook for systematic reviews of interventions: John Wiley & Sons; 2019.
22. National Heart LaBI. Study quality assessment tool for observational and cross sectional studies. 2021; Available from: https://www.nhlbi.nih.gov/health-topics/study-quality-assessment-tools.
23. Butler A, Hall H, Copnell B. A Guide to Writing a Qualitative Systematic Review Protocol to Enhance Evidence-Based Practice in Nursing and Health Care. Worldviews Evid Based Nurs. 2016; 13:241-9.
24. Saini M, Shlonsky A. Systematic synthesis of qualitative research: OUP USA; 2012.
25. Beaton L, Freeman R. Oral health promotion and homelessness: A theory-based approach to understanding processes of implementation and adoption. Health Education Journal. 2016; 75:184-97.
26. Beaton L, Anderson I, Humphris G, Rodriguez A, Freeman R. Implementing an Oral Health Intervention for People Experiencing Homelessness in Scotland: A Participant Observation Study. Dent J (Basel). 2018; 6.
27. Beaton L, Humphris G, Rodriguez A, Freeman R. Implementing the Smile4life intervention for people experiencing homelessness: a path analytical evaluation. BMC Oral Health. 2021; 21:383.
28. Rodriguez A, Beaton L, Freeman R. Strengthening Social Interactions and Constructing New Oral Health and Health Knowledge: The Co-design, Implementation and Evaluation of A Pedagogical Workshop Program with and for Homeless Young People. Dent J (Basel). 2019; 7.
29. Coles E, Watt C, Freeman R. 'Something to Smile About': An evaluation of a capacity-building oral health intervention for staff working with homeless people. Health Education Journal. 2012; 72:146-55.
30. Stormon N, Pradhan A, McAuliffe A, Ford P. Does a facilitated pathway improve access to dental services for homeless and disadvantaged adults? Evaluation and program planning. 2018; 71:46-50.
31. Doughty J, Johnson A, Tatari A, Kothari C, Patel R, Newman A, et al. A pilot denture service collaboration between Den-Tech denture charity and Crisis at Christmas Dental Service (CCDS): A Plan-Do-Study-Act analysis. British Dental Journal. 2020; 228:183-90.
32. Paisi M, Baines R, Worle C, Withers L, Witton R. Evaluation of a community dental clinic providing care to people experiencing homelessness: A mixed methods approach. Health Expectations. 2020; 23:1289-99.
33. Neale J, Stevenson C. Positive and negative features of a computer assisted drug treatment program delivered by mentors to homeless drug users living in hostels. Journal of substance abuse treatment. 2014; 47:258-64.
34. Burnam MA, Morton SC, McGlynn EA, Petersen LP, Stecher BM, Hayes C, et al. An experimental evaluation of residential and nonresidential treatment for dually diagnosed homeless adults. J Addict Dis. 1995; 14:111-34.
35. Collins SE, Clifasefi SL, Nelson LA, Stanton J, Goldstein SC, Taylor EM, et al. Randomized controlled trial of harm reduction treatment for alcohol (HaRT-A) for people experiencing homelessness and alcohol use disorder. Int J Drug Policy. 2019; 67:24-33.
36. Forchuk C, Richardson J, Atyeo H, Serrato J. Qualitative findings from a Housing First evaluation project for homeless Veterans in Canada. Journal of Military, Veteran and Family Health. 2022; 8:27-38.

For peer review only

37. Henderson PM. Program evaluation of a long-term substance abuse treatment program for homeless men: Spalding University; 2003.
38. Pauly B, King V, Smith A, Tranquilli-Doherty S, Wishart M, Vallance K, et al. Breaking the cycle of survival drinking: insights from a non-residential, peer-initiated and peer-run managed alcohol program. Drugs: Education, Prevention and Policy. 2021; 28:172-80.
39. Rash CJ, Alessi SM, Petry NM. Substance abuse treatment patients in housing programs respond to contingency management interventions. Journal of Substance Abuse Treatment. 2017; 72:97-102.
40. Pratt R, Pernat C, Kerandi L, Kmiecik A, Strobel-Ayres C, Joseph A, et al. "It'sa hard thing to manage when you're homeless": the impact of the social environment on smoking cessation for smokers experiencing homelessness. BMC Public Health. 2019; 19:1-8.
41. Pratt R, Xiong S, Kmiecik A, Strobel-Ayres C, Joseph A, Rose SAE, et al. The implementation of a smoking cessation and alcohol abstinence intervention for people experiencing homelessness. BMC public health. 2022; 22:1260.
42. Baggett TP, Singer DE, Rao SR, O'Connell JJ, Bharel M, Rigotti NA. Food Insufficiency and Health Services Utilization in a National Sample of Homeless Adults. Journal of General Internal Medicine. 2011; 26:627-34.
43. Fallaize R, Seale JV, Mortin C, Armstrong L, Lovegrove JA. Dietary intake, nutritional status and mental wellbeing of homeless adults in Reading, UK. British Journal of Nutrition. 2017; 118:707-14.
44. Okuyemi KS, Caldwell AR, Thomas JL, Born W, Richter KP, Nollen N, et al. Homelessness and Smoking Cessation: Insights from Focus Groups. Nicotine Tob Res. 2006; 8:287-96.
45. Langnäse K, Müller MJ. Nutrition and health in an adult urban homeless population in Germany. Public Health Nutrition. 2001; 4:805-11.
46. Knudsen HK. Implementation of smoking cessation treatment in substance use disorder treatment settings: a review. The American Journal of Drug and Alcohol Abuse. 2017; 43:215-25.
47. Kim JE, Flentje A, Tsoh JY, Riley ED. Cigarette Smoking among Women Who Are Homeless or Unstably Housed: Examining the Role of Food Insecurity. J Urban Health. 2017; 94:514-24.
48. Beaton L, Anderson I, Humphris G, Rodriguez A, Freeman R. Implementing an Oral Health Intervention for People Experiencing Homelessness in Scotland: A Participant Observation Study. Dentistry Journal. 2018; 6:68.
49. Paisi M, Kay E, Plessas A, Burns L, Quinn C, Brennan N, et al. Barriers and enablers to accessing dental services for people experiencing homelessness: A systematic review. Community dentistry and oral epidemiology. 2019; 47:103-11.
50. Soar K, Dawkins L, Robson D, Cox S. Smoking amongst adults experiencing homelessness: a systematic review of prevalence rates, interventions and the barriers and facilitators to quitting and staying quit. Journal of Smoking Cessation. 2020; 15:94-108.
51. Xiang X. A review of interventions for substance use among homeless youth. Research on Social Work Practice. 2013; 23:34-45.
52. Nyamathi A, Kennedy B, Branson C, Salem B, Khalilifard F, Marfisee M, et al. Impact of Nursing Intervention on Improving HIV, Hepatitis Knowledge and Mental Health Among Homeless Young Adults. Community Mental Health Journal. 2013; 49:178-84.
53. Young SD, Rice E. Online social networking technologies, HIV knowledge, and sexual risk and testing behaviors among homeless youth. AIDS and Behavior. 2011; 15:253-60.

For peer review only

54. Slesnick N, Kang MJ. The impact of an integrated treatment on HIV risk behavior among homeless youth: a randomized controlled trial. Journal of behavioral medicine. 2008; 31:45-59.

For peer review only

Figure 1*: PRISMA Flowchart for the search results.*

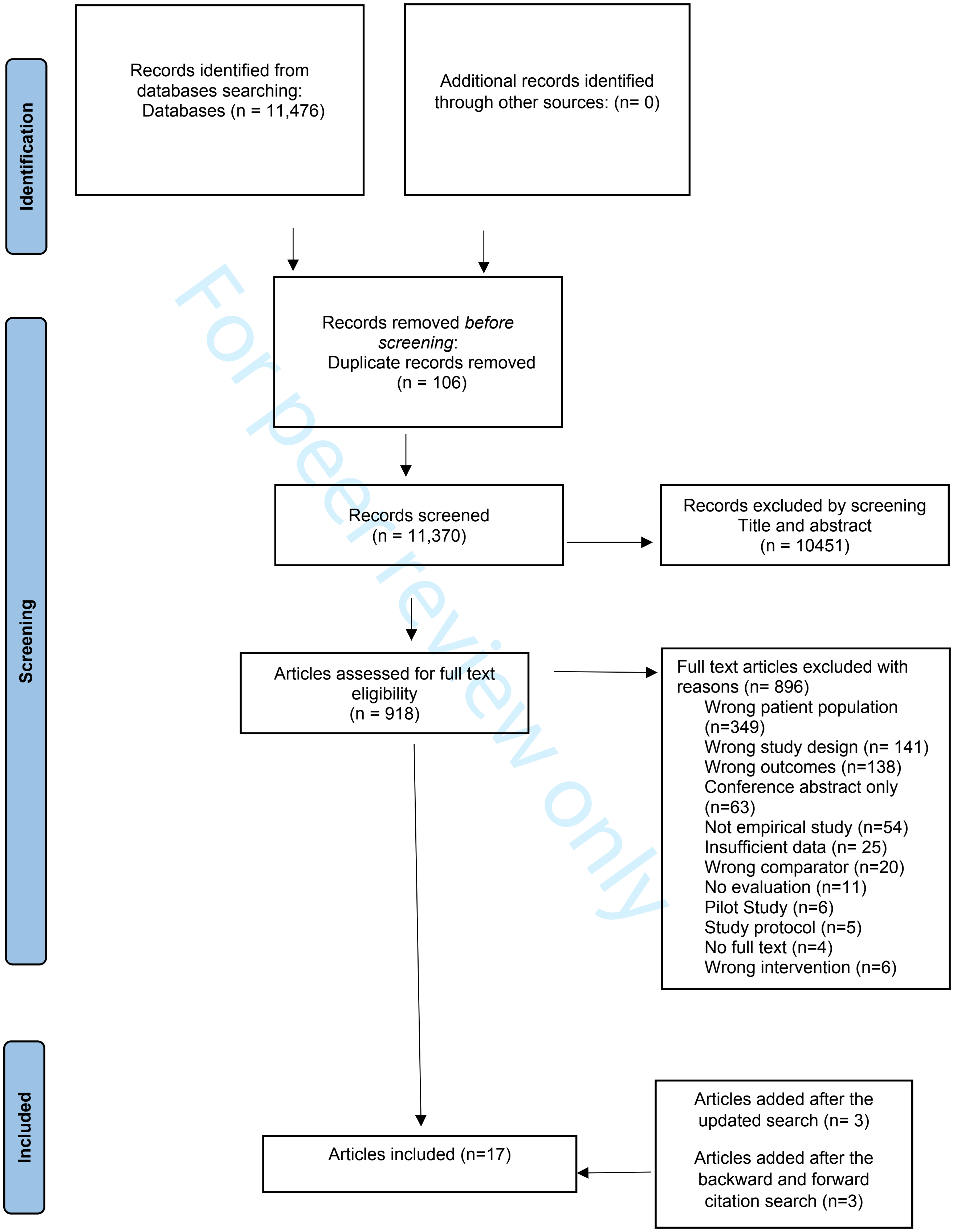

For peer review only

Appendix A: Search Strategy

#	Searches
1	Homeless Persons/
2	homeless*.ti,ab,kw,kf.
3	((hous* or home* or accommodat* or shelter) adj3 (insecur* or instability or unstable or stability)).ti,ab,kw,kf.
4	(sleep* adj2 rough).ti,ab,kw,kf.
5	squatter*.ti,ab,kw,kf.
6	shelter.ti,ab,kw,kf.
7	"sofa surf*".ti,ab,kw,kf.
8	or/1-7
9	((severe or multiple) adj disadvantage*).ti,ab,kw,kf.
10	"social exclusion".ti,ab,kw,kf.
11	"complex needs".ti,ab,kw,kf.
12	"marginali?ed populations".ti,ab,kw,kf.
13	or/9-12
14	exp Substance-Related Disorders/
15	Behavior, Addictive/
16	addict*.ti,ab,kw,kf.
17	((alcohol or drug or substance) adj1 (misuse or abuse or use* or addict* or dependenc* or issue* or problem)).ti,ab,kw,kf.
18	Alcoholics/
19	alcoholic*.ti,ab,kw,kf.
20	(drug adj1 (habit or tak* or hard or illicit or inject*)).ti,ab,kw,kf.
21	exp Illicit Drugs/
22	Alcohol Drinking/
23	"street drink*".ti,ab,kw,kf.
24	or/14-23
25	Prisoners/
26	prisoner*.ti,ab,kw,kf.
27	Criminals/
28	criminal*.ti,ab,kw,kf.
29	((repeat or ex or re or revolving door) adj1 offen*).ti,ab,kw,kf.
30	(convict or convicts or "convicted person*").ti,ab,kw,kf.
31	or/25-30
32	8 or 13 or 24 or 31
33	Oral Health/
34	("oral health" or "dental health").ti,ab,kw,kf.
35	Oral Hygiene/
36	"oral hygiene".ti,ab,kw,kf.
37	Mouth Rehabilitation/
38	"mouth rehabilitation*".ti,ab,kw,kf.
39	Dental Health Services/
40	"dental health service*".ti,ab,kw,kf.
41	Dental Care/
42	"dental care".ti,ab,kw,kf.
43	exp Dental Caries/

For peer review only

44	"dental caries".ti,ab,kw,kf.
45	Dental Enamel Solubility/
46	"dental enamel solubility".ti,ab,kw,kf.
47	Dental Deposits/
48	"dental deposits".ti,ab,kw,kf.
49	Dentin Sensitivity/
50	"dentin sensitivity".ti,ab,kw,kf.
51	Dental Plaque/
52	"dental plaque".ti,ab,kw,kf.
53	exp Dental Pulp Diseases/
54	"dental pulp disease*".ti,ab,kw,kf.
55	Tooth Loss/
56	"tooth loss".ti,ab,kw,kf.
57	"loss of teeth".ti,ab,kw,kf.
58	Tooth Diseases/
59	("tooth disease" or "diseased teeth").ti,ab,kw,kf.
60	Toothache/
61	(toothache or "tooth ache").ti,ab,kw,kf.
62	Tooth Demineralization/
63	Tooth Mobility/
64	Tooth Discoloration/
65	(tooth adj1 (demineralization or mobility or decay or discolo?ration)).ti,ab,kw,kf.
66	Mouth Diseases/
67	Periodontal Diseases/
68	Gingival Diseases/
69	((mouth or peridontal or gingival or gum) adj1 disease*).ti,ab,kw,kf.
70	exp Periodontitis/
71	periodontitis.ti,ab,kw,kf.
72	Preventive Dentistry/
73	"Preventive Dentistry".ti,ab,kw,kf.
74	exp Gingivitis/
75	((oral or dental or tooth or teeth) adj1 abscess).ti,ab,kw,kf.
76	(dental adj1 (pain or sequelae)).ti,ab,kw,kf.
77	"bleeding gum*".ti,ab,kw,kf.
78	(hole* adj2 (tooth or teeth)).ti,ab,kw,kf.
79	or/33-78
80	exp Mouth Neoplasms/
81	((oral or mouth or tongue or salivary or parotid) adj1 (cancer* or tumour* or tumor* or neoplasm*)).ti,ab,kw,kf.
82	80 or 81
83	79 or 82
84	exp Smoking/
85	(smoking or smoker or smoke or smoked or smokes).ti,ab,kw,kf.
86	Tobacco/
87	"Tobacco Use Cessation"/ or exp "Tobacco Use"/ or exp "Tobacco Use Cessation Devices"/
88	Smoking Cessation/
89	"smoking cessation*".ti,ab,kw,kf.

For peer review only

90	Tobacco Products/
91	Nicotine/
92	(tobacco or cigar* or e-cig* or nicotine or hookah or pipe or vaping or vape).ti,ab,kw,kf.
93	or/84-92
94	((sugar or sucrose or fructose or glucose) adj2 (intake or consum*)).ti,ab,kw,kf.
95	"high sugar diet".ti,ab,kw,kf.
96	"sugary foods".ti,ab,kw,kf.
97	exp Dietary Sugars/
98	((processed or acidic) adj1 food*).ti,ab,kw,kf.
99	((sugary or fizzy or carbonated or soft) adj1 drink*).ti,ab,kw,kf.
100	carbonated beverages/ or sugar-sweetened beverages/
101	soda.ti,ab,kw,kf.
102	or/94-101
103	((abuse or sniff*) adj3 (solvent* or glue or gas or aerosol* or inhalant)).ti,ab,kw,kf.
104	addict*.ti,ab,kw,kf.
105	((alcohol or drug or substance) adj1 (misuse or abuse or use* or addict* or dependen* or issue* or problem or prevention or treatment or recovery or habit)).ti,ab,kw,kf.
106	alcoholic*.ti,ab,kw,kf.
107	Alcoholics/
108	(drug adj1 (tak* or hard or illicit or inject*)).ti,ab,kw,kf.
109	exp Illicit Drugs/
110	"street drink*".ti,ab,kw,kf.
111	or/103-110
112	83 or 93 or 102 or 111
113	32 and 112
114	intervention*.ti,ab,kw.
115	program*.ti,ab,kw.
116	(care adj3 package*).ti,ab,kw.
117	feasab*.ti,ab,kw.
118	acceptab*.ti,ab,kw.
119	efficacy.ti,ab,kw.
120	effective*.ti,ab,kw.
121	training.ti,ab,kw.
122	educat*.ti,ab,kw.
123	evaluat*.ti,ab,kw.
124	strateg*.ti,ab,kw.
125	pilot.ti,ab,kw.
126	perception*.ti,ab,kw,kf.
127	belief*.ti,ab,kw,kf.
128	uptake.ti,ab,kw,kf.
129	impact.ti,ab,kw,kf.
130	consequence*.ti,ab,kw,kf.
131	attitude*.ti,ab,kw,kf.
132	barrier*.ti,ab,kw,kf.
133	facilitat*.ti,ab,kw,kf.

For peer review only

134	motivat*.ti,ab,kw,kf.
135	or/114-134
136	113 and 135
137	limit 136 to english language

For peer review only

PRISMA 2020 Checklist

Section and Topic	Item #	Checklist item	Location where item is reported
**TITLE**			
Title	1	Identify the report as a systematic review.	Lines 2-3
**ABSTRACT**			
Abstract	2	See the PRISMA 2020 for Abstracts checklist.	Page 1
**INTRODUCTION**			
Rationale	3	Describe the rationale for the review in the context of existing knowledge.	Lines 38 - 44
Objectives	4	Provide an explicit statement of the objective(s) or question(s) the review addresses.	Lines 54-56
**METHODS**			
Eligibility criteria	5	Specify the inclusion and exclusion criteria for the review and how studies were grouped for the syntheses.	Table 1
Information sources	6	Specify all databases, registers, websites, organisations, reference lists and other sources searched or consulted to identify studies. Specify the date when each source was last searched or consulted.	Lines 63-68
Search strategy	7	Present the full search strategies for all databases, registers and websites, including any filters and limits used.	Appendix A.
Selection process	8	Specify the methods used to decide whether a study met the inclusion criteria of the review, including how many reviewers screened each record and each report retrieved, whether they worked independently, and if applicable, details of automation tools used in the process.	Lines 70 - 73
Data collection process	9	Specify the methods used to collect data from reports, including how many reviewers collected data from each report, whether they worked independently, any processes for obtaining or confirming data from study investigators, and if applicable, details of automation tools used in the process.	Lines 85 - 91
Data items	10a	List and define all outcomes for which data were sought. Specify whether all results that were compatible with each outcome domain in each study were sought (e.g. for all measures, time points, analyses), and if not, the methods used to decide which results to collect.	Table 1
	10b	List and define all other variables for which data were sought (e.g. participant and intervention characteristics, funding sources). Describe any assumptions made about any missing or unclear information.	Table 1 and Table 2
Study risk of bias assessment	11	Specify the methods used to assess risk of bias in the included studies, including details of the tool(s) used, how many reviewers assessed each study and whether they worked independently, and if applicable, details of automation tools used in the process.	Lines 75 - 83
Effect measures	12	Specify for each outcome the effect measure(s) (e.g. risk ratio, mean difference) used in the synthesis or presentation of results.	Table 2
Synthesis methods	13a	Describe the processes used to decide which studies were eligible for each synthesis (e.g. tabulating the study intervention characteristics and comparing against the planned groups for each synthesis (item #5)).	Table 1
	13b	Describe any methods required to prepare the data for presentation or synthesis, such as handling of missing summary statistics, or data conversions.	Lines 85 - 91
	13c	Describe any methods used to tabulate or visually display results of individual studies and syntheses.	Lines 85 - 91
	13d	Describe any methods used to synthesize results and provide a rationale for the choice(s). If meta-analysis was performed, describe the model(s), method(s) to identify the presence and extent of statistical heterogeneity, and software package(s) used.	Liness 85 - 91
	13e	Describe any methods used to explore possible causes of heterogeneity among study results (e.g. subgroup analysis, meta-regression).	N/A
	13f	Describe any sensitivity analyses conducted to assess robustness of the synthesized results.	N/A
Reporting bias	14	Describe any methods used to assess risk of bias due to missing results in a synthesis (arising from reporting biases).	Lines 75 -

For peer review only - http://bmjopen.bmj.com/site/about/guidelines.xhtml

PRISMA 2020 Checklist

Section and Topic	Item #	Checklist item	Location where item is reported
assessment			83
Certainty assessment	15	Describe any methods used to assess certainty (or confidence) in the body of evidence for an outcome.	Lines 75 - 83
**RESULTS**			
Study selection	16a	Describe the results of the search and selection process, from the number of records identified in the search to the number of studies included in the review, ideally using a flow diagram.	Figure 1
	16b	Cite studies that might appear to meet the inclusion criteria, but which were excluded, and explain why they were excluded.	Figure 1
Study characteristics	17	Cite each included study and present its characteristics.	Table 2
Risk of bias in studies	18	Present assessments of risk of bias for each included study.	Table 2
Results of individual studies	19	For all outcomes, present, for each study: (a) summary statistics for each group (where appropriate) and (b) an effect estimate and its precision (e.g. confidence/credible interval), ideally using structured tables or plots.	Table 1
Results of syntheses	20a	For each synthesis, briefly summarise the characteristics and risk of bias among contributing studies.	Lines 102 - 108
	20b	Present results of all statistical syntheses conducted. If meta-analysis was done, present for each the summary estimate and its precision (e.g. confidence/credible interval) and measures of statistical heterogeneity. If comparing groups, describe the direction of the effect.	N/A
	20c	Present results of all investigations of possible causes of heterogeneity among study results.	N/A
	20d	Present results of all sensitivity analyses conducted to assess the robustness of the synthesized results.	N/A
Reporting biases	21	Present assessments of risk of bias due to missing results (arising from reporting biases) for each synthesis assessed.	Lines 103 - 108
Certainty of evidence	22	Present assessments of certainty (or confidence) in the body of evidence for each outcome assessed.	N/A
**DISCUSSION**			
Discussion	23a	Provide a general interpretation of the results in the context of other evidence.	Lines 223 - 251
	23b	Discuss any limitations of the evidence included in the review.	Lines 274 - 277
	23c	Discuss any limitations of the review processes used.	Lines 277 - 278
	23d	Discuss implications of the results for practice, policy, and future research.	Lines 281 - 288
**OTHER INFORMATION**			
Registration and protocol	24a	Provide registration information for the review, including register name and registration number, or state that the review was not registered.	Lines 58 - 59
	24b	Indicate where the review protocol can be accessed, or state that a protocol was not prepared.	Lines 58 - 59

For peer review only

PRISMA 2020 Checklist

Section and Topic	Item #	Checklist item	Location where item is reported
	24c	Describe and explain any amendments to information provided at registration or in the protocol.	
Support	25	Describe sources of financial or non-financial support for the review, and the role of the funders or sponsors in the review.	Lines 295 - 300
Competing interests	26	Declare any competing interests of review authors.	Lines 302-304
Availability of data, code and other materials	27	Report which of the following are publicly available and where they can be found: template data collection forms; data extracted from included studies; data used for all analyses; analytic code; any other materials used in the review.	N/A

From: Page MJ, McKenzie JE, Bossuyt PM, Boutron I, Hoffmann TC, Mulrow CD, et al. The PRISMA 2020 statement: an updated guideline for reporting systematic reviews. BMJ 2021;372:n71. doi: 10.1136/bmj.n71

For more information, visit: http://www.prisma-statement.org/

For peer review only

BMJ Open

Factors influencing implementation and sustainability of interventions to improve oral health and related health behaviours in adults experiencing severe and multiple disadvantage: A mixed-methods systematic review

Journal:	*BMJ Open*
Manuscript ID	bmjopen-2023-080160.R1
Article Type:	Original research
Date Submitted by the Author:	04-Dec-2023
Complete List of Authors:	John , Deepti; Newcastle University Population Health Sciences Institute<br>Adams, Emma; Newcastle University Population Health Sciences Institute<br>McGowan, Laura; Newcastle University Population Health Sciences Institute<br>Joyes, Emma; Newcastle University Population Health Sciences Institute<br>Richmond, Catherine; Newcastle University Population Health Sciences Institute<br>Beyer, Fiona ; Newcastle University Population Health Sciences Institute, Evidence Synthesis Group and Innovation Observatory<br>Landes, David; NHS England and NHS Improvement North East and Yorkshire<br>Watt, Richard; University College London, Epidemiology and Public Health; King's College London, King's College Dental Institute<br>Sniehotta, Falko; Newcastle University Population Health Sciences Institute, NIHR Policy Research Unit Behavioural Science; Heidelberg University Medical Faculty Mannheim, Department of Public Health, Social and Preventive Medicine, Centre for Preventive Medicine and Digital Health (CPD)<br>Paisi, Martha; Plymouth University Peninsula Dental School<br>Bambra, Clare; Newcastle University Population Health Sciences Institute<br>Craig, Dawn; Newcastle University Population Health Sciences Institute<br>Kaner, Eileen; Newcastle University Population Health Sciences Institute<br>Ramsay, Sheena; Newcastle University Population Health Sciences Institute
**Primary Subject Heading**:	Public health
Secondary Subject Heading:	Health policy, Smoking and tobacco, Dentistry and oral medicine
Keywords:	Health Equity, PUBLIC HEALTH, Behavior

SCHOLARONE™
Manuscripts

I, the Submitting Author has the right to grant and does grant on behalf of all authors of the Work (as defined in the below author licence), an exclusive licence and/or a non-exclusive licence for contributions from authors who are: i) UK Crown employees; ii) where BMJ has agreed a CC-BY licence shall apply, and/or iii) in accordance with the terms applicable for US Federal Government officers or employees acting as part of their official duties; on a worldwide, perpetual, irrevocable, royalty-free basis to BMJ Publishing Group Ltd ("BMJ") its licensees and where the relevant Journal is co-owned by BMJ to the co-owners of the Journal, to publish the Work in this journal and any other BMJ products and to exploit all rights, as set out in our licence.

The Submitting Author accepts and understands that any supply made under these terms is made by BMJ to the Submitting Author unless you are acting as an employee on behalf of your employer or a postgraduate student of an affiliated institution which is paying any applicable article publishing charge ("APC") for Open Access articles. Where the Submitting Author wishes to make the Work available on an Open Access basis (and intends to pay the relevant APC), the terms of reuse of such Open Access shall be governed by a Creative Commons licence – details of these licences and which Creative Commons licence will apply to this Work are set out in our licence referred to above.

Other than as permitted in any relevant BMJ Author's Self Archiving Policies, I confirm this Work has not been accepted for publication elsewhere, is not being considered for publication elsewhere and does not duplicate material already published. I confirm all authors consent to publication of this Work and authorise the granting of this licence.

For peer review only

Factors influencing implementation and sustainability of interventions to improve oral health and related health behaviours in adults experiencing severe and multiple disadvantage: A mixed-methods systematic review

Authors
Mrs. Deepti A. John, MDPH - Population Health Sciences Institute, Newcastle University, Newcastle Upon Tyne NE2 4AX, UK
Ms. Emma A. Adams, MSc. - Population Health Sciences Institute, Newcastle University, Newcastle Upon Tyne NE2 4AX, UK
Dr. Laura J. McGowan, PhD - Population Health Sciences Institute, Newcastle University, Newcastle Upon Tyne NE2 4AX, UK
Dr. Emma C. Joyes, PhD - Population Health Sciences Institute, Newcastle University, Newcastle Upon Tyne NE2 4AX, UK
Ms. Catherine Richmond, MA - Population Health Sciences Institute, Newcastle University, Newcastle Upon Tyne NE2 4AX, UK
Dr. Fiona R. Beyer, PhD - Evidence Synthesis Group and Innovation Observatory, Population Health Sciences Institute, Newcastle University, Newcastle Upon Tyne NE2 4AX, UK
Mr. David Landes, BDS - NHS England & Improvement, Newcastle Upon Tyne NE15 8NY, UK
Prof. Richard Watt, PhD - Department of Epidemiology and Public Health, University College London, London WC1E 7HB, UK
Prof. Falko F. Sniehotta, PhD - NIHR Policy Research Unit Behavioural Science, Newcastle University, NE2 4AX And Department of Public Health, Social and Preventive Medicine, Centre for Preventive Medicine and Digital Health (CPD), Medical Faculty Mannheim, Heidelberg University, 68167 Mannheim
Dr. Martha Paisi, PhD - Faculty of Medicine and Dentistry, Peninsula Dental School, University of Plymouth, Plymouth PL4 8AA, UK
Prof. Clare Bambra, PhD - Population Health Sciences Institute, Newcastle University, Newcastle Upon Tyne NE2 4AX, UK
Prof. Dawn Craig, MSc. - Population Health Sciences Institute, Newcastle University, Newcastle Upon Tyne NE2 4AX, UK
Prof. Eileen Kaner, PhD - Population Health Sciences Institute, Newcastle University, Newcastle Upon Tyne NE2 4AX, UK
Prof. Sheena E. Ramsay, PhD - Population Health Sciences Institute, Newcastle University, Newcastle Upon Tyne NE2 4AX, UK

Corresponding Author:
Deepti John
Deepti.john@newcastle.ac.uk
Phone no: +44 7469646623
Population Health Sciences Institute, Newcastle University
The Baddiley-Clark Building, Richardson Road
Newcastle upon Tyne, NE2 4AX
United Kingdom

For peer review only

Abstract

Objectives

Among people experiencing severe and multiple disadvantage (SMD) poor oral health is common and linked to smoking, substance use and high sugar intake. Studies have explored interventions addressing oral health and related behaviours; however, factors related to the implementation of these interventions remains unclear. This mixed-methods systematic review aimed to synthesize evidence on the implementation and sustainability of interventions to improve oral health and related health behaviours among adults experiencing SMD.

Methods

Bibliographic databases (MEDLINE, EMBASE, PsycINFO, CINAHL, EBSCO, Scopus) and grey literature were searched from inception to February 2023. Studies meeting the inclusion criteria were screened and extracted independently by two researchers. Quality appraisal was undertaken, and results were synthesised using narrative and thematic analyses.

Results

Seventeen papers were included (published between 1995-2022). Studies were mostly of moderate quality and included views from SMD groups and service providers. From the qualitative synthesis, most findings were related to aspects such as trust, resources, and motivation levels of SMD groups and service providers. None of the studies reported on diet and none included repeated offending (one of the aspects of SMD). From the quantitative synthesis, no difference was observed in program attendance between the interventions and usual care, although there was some indication of sustained improvements in participation in the intervention group.

Conclusion

This review provides some evidence that trust, adequate resources, and motivation levels are potentially important in implementing interventions to improve oral health and substance use among SMD groups. Further research is needed from high quality studies and focusing on diet in this population.

Strengths and limitations of this study

- Comprehensive search strategy was used to gather evidence in this mixed methods systematic review.

For peer review only

- Consolidated Framework for Implementation Research (CFIR) was used for the data extraction.
- Confidence in the papers were limited due to moderate quality of the papers.
- The included studies were not excluded based on their quality, as they contributed relevant information for this systematic review.

Keywords: multiple disadvantage, homeless, oral health, implementation

INTRODUCTION

Severe and multiple disadvantaged (SMD) populations are individuals who have experienced homelessness, substance use, offending or a combination of all three.(1) They experience disproportionately high levels of poor physical and mental health along with high levels of occupational deprivation,(1) which results in isolation and difficulty in accessing healthcare services.(2) There is also an added burden of stigma that affects their access and engagement.(3)

Among people experiencing SMD, oral health problems have been highlighted as one of the major unmet needs.(4) Aggravated by high levels of smoking, substance and alcohol use and poor diet (high intake of sugar).(4, 5) Elevated tobacco use make them more susceptible to periodontal disease, tooth loss, oral lesions and oral cancer.(5, 6) Research also shows that they do not meet the daily nutritional requirements and have high levels of sugar consumption.(6, 7) Oral health has an overall impact on physical and mental wellbeing.(8) It is, therefore, important to not only address oral health concerns in people experiencing SMD, but also related health behaviours such as smoking, alcohol and substance use, and poor nutrition.(1, 9)

Previous papers focus on intervention design and outcomes, none focus on the implementation approach of these intervention especially in people experiencing SMD.(10, 11, 12) Hence, there is a need for evidence on interventions addressing these health challenges, with a specific focus on ways to improve implementation and long-term sustainability of interventions. Frameworks are used to apply a theoretical underpinning to our understanding of why implementation of interventions succeed or fail. The Consolidated Framework for implementation Research (CFIR) composed of five domains was utilised as a theoretical framework to identify the facilitators and barriers that influence implementation. (13, 14) This framework, therefore, assists with bridging the gap between research and practice as well as reducing the challenges of implementing these interventions.(15)

To investigate how we can improve implementation and sustainability, we conducted this systematic review to synthesize various factors such as acceptability, settings, and potential adverse effects of interventions that improve oral health and related health behaviours of adults with SMD.

For peer review only - http://bmjopen.bmj.com/site/about/guidelines.xhtml

METHODS

The research protocol was pre-registered and published registered with the Prospective Register of Systematic Reviews (PROSPERO) (reg. no: CRD42020202416).(16, 17) The review was reported according to Preferred Reporting Items for Systematic Reviews and Meta-Analyses (PRISMA) guidelines.(18)

Search strategy

The search strategy (see Supplementary file.) was formulated and conducted with an information specialist within the research team. The following electronic databases: MEDLINE (Ovid), EMBASE (Ovid), CINAHL (Ebsco), APA PsycINFO (Ovid) and Scopus were searched for relevant qualitative, quantitative, and mixed method studies from inception to February 2023. Grey literature searches were conducted using Google Incognito and selected charity organization websites such as Fulfilling Lives, Crisis, Groundswell, which were informed by the expertise of the research team. Forward and backward citation search of the included studies were also conducted.

Study selection

The search results were downloaded and deduplicated using EndNote 20.4.1 and the uploaded into Covidence, an online tool for managing the whole systematic review process.(19) Title, abstracts and full texts were independently screened by two reviewers. In the case of a discrepancy, consensus was reached after consultation with a third reviewer. Table 1 presents the inclusion criteria used during screening.

Table 1: *Eligibility Criteria used to select the studies*

	Eligibility Criteria
Population	Adults aged 18 or above, who experience SMD comprising of either homelessness (rough sleeping or other types of insecure accommodation), repeated offending or frequent substance use that co-occurs with homelessness or repeated offending.(17) Perspectives of staff who work with SMD groups and stakeholders such as policy makers and commissioners.
Intervention	Structural, community and individual level interventions.(17)
Outcomes	Views from SMD groups and other stakeholders (policy makers, service providers, voluntary sector etc) about implementation and sustainability of interventions which include acceptability, content, settings, potential harms, uptake, and retention.(17)
Study Design	Qualitative, quantitative, and mixed method studies

Data extraction and quality appraisal

The data extraction and quality assessment for all the included studies were conducted by one reviewer and cross-checked by a second reviewer. Included studies were critically appraised to guide

For peer review only - http://bmjopen.bmj.com/site/about/guidelines.xhtml

how much confidences could be placed on the findings. Qualitative studies were appraised using the Critical Appraisal Skills Programme (CASP) qualitative checklist.(20) Quantitative studies were appraised using Cochrane's Risk of bias for randomized control trials (RCTs).(21) For cross-sectional studies the National Institutes of Health (NIH) Study Quality Appraisal Tool was used.(22) Qualitative studies were rated as good, moderate or low quality, which was informed by a scoring system: scores 9–10 was high quality, 7.5–9 was moderate quality and <7.5 was low quality.(23) The scoring were informed by the quality checklists. Studies were not excluded based on their quality; poor reporting is not always reflective of poor methodology. (24) Studies were included on whether they contributed data relevant or novel data to this review.(24) Moreover, including all studies allowed gathering the global evidence related to the review questions.

Data synthesis

Abstracts and data from the results of included studies were uploaded on to NVivo software (QSR International Pty Ltd., Melbourne, Australia Version 12, Release 1.6.1). Narrative synthesis was undertaken. Deductive codes based on the CFIR framework were used to initially code the findings followed by a three-step inductive synthesis process which involved coding the text, identifying the themes, and creating the subthemes. To maximise thematic yield, data reported in different papers but from the same study were individually coded. The developing themes and subthemes were discussed with the other reviewers and consensus was reached regarding these.

Patient and public involvement

Patients and/or the public were not involved in the design, or conduct, or reporting, or dissemination plans of this research.

RESULTS

Seventeen articles (twelve individual studies) met the inclusion criteria and were included in this systematic review. Figure 1 presents the PRISMA flowchart for included studies. Table 2 presents the descriptive summaries of the included studies. The papers were published between 1995 and 2022, and were related to interventions targeting oral health,(25, 26, 27, 28, 29, 30, 31, 32) substance use,(33, 34, 35, 36, 37, 38, 39) smoking and none on diet.

For peer review only - http://bmjopen.bmj.com/site/about/guidelines.xhtml

Table 2: *Descriptive summaries of the seventeen included studies, including quality appraisal (High quality/moderate quality/low quality)*

No.	First Author (Year) and Country	Sample Size and Age of the participants	Participant Group	Intervention Description	Type of Research, Data Collection and Analysis	Findings of the paper relevant to the review	Quality Appraisal/ Risk of Bias
1.	Beaton et al (2016) United Kingdom(25)	N=20, age not mentioned	Health and social care workers	Motivational interviewing to promote oral health among homeless populations ("Smile4life programme")	Qualitative - Telephone interviews, framework approach	Familiarity and good relationships between service providers and third sector organizations facilitated implementation whereas lack of resources and interest hindered it	High quality
2.	Beaton et al (2018) United Kingdom (26)	N= 9 observation sessions, age not mentioned	Oral healthcare workers such as oral health educators and dental support workers	Motivational interviewing and tailored advice to promote oral health among the homeless population at different settings such as mobile dental units and homeless shelters ("Smile4Life programme")	Qualitative- Participant observation, content analysis	Good working relationships between healthcare providers, patients and third sector organizations are important	Moderate quality
3.	Beaton et al (2021) United Kingdom (27)	N= 100, 16- 85 years	Oral health practitioners, third sector organization staff and local authority staff	Motivational interviewing and behavioural change techniques to promote oral health among the homeless ("Smile4Life programme")	Quantitative- Questionnaire, K-R20, Exploratory factor analysis, multivariate path analysis.	Work practices such as positive attitudes and beliefs of the oral healthcare workers influence implementation	Moderate/ Fair quality
4.	Burnam et al (1995) United States of America(34)	N= 276, mean age = 37 years	Homeless individuals with co-occurring substance and mental health issues	Social model of residential and non-residential programs providing integrated substance use and mental health services	Quantitative- Structured interviews, regression analyses	Retention levels were higher in the residential program compared to the non-residential one	Low quality (high Risk of Bias)
5.	Coles et al (2013) United Kingdom(29)	N= 14, age not mentioned	Healthcare workers from statutory and non-statutory organizations	A framework that offers tailored oral health advice and signposts to relevant dental services. ("Something To Smile About")	Qualitative- Focus groups, content analysis	Oral health knowledge among the healthcare workers improved but complex needs such as housing, employment etc must be addressed prior to oral health for successful implementation	Moderate quality
6.	Collins et al (2019) United States of America(35)	N= 168, mean age= 47 years	Homeless individuals with alcohol use disorder	Non-abstinence treatment program that involves tracking of alcohol use, discussion of safe drinking practices and goal-oriented tasks. ("Harm Reduction Treatment for Alcohol HaRT-A")	Quantitative-Questionnaires, content analysis	It was positively viewed by the participants with high levels of retention and satisfaction	Good quality (low ROB)
7.	Doughty et al (2020) United Kingdom(31)	Service users – N= 353, age not mentioned Service providers – not stated	Homeless individuals and oral healthcare workers such as dentists, dental nurses, dental technicians etc	Denture service provided by Crisis at Christmas Dental Service and Den-tech to the homeless and vulnerably housed	Qualitative	Communication, timing, resources, and training were considered as areas that needed to be improved	Low quality
8.	Forchuk et al (2022) Canada(36)	Service users – N= 58, mean age = 52.5 years Service providers – not stated	Homeless veterans with substance use problems and staff from housing services	Housing provided along with the peer support and harm reduction services to homeless veterans ("Housing First")	Qualitative - Interviews and focus groups, Thematic analysis	Stable housing with harm reduction services was well received. Collaboration between mental health and addiction services should be considered for future services	Low quality
9.	Henderson et al (2004) United States of America(37)	Service users – N = 15 Service providers – not mentioned	Homeless veterans with substance/alcohol use and program staff such as	Residential substance use treatment program that focuses on relapse prevention along with education and housing stability for homeless men	Qualitative - Surveys, direct observation and Interviews, not stated	Majority of the participants provided positive feedback. Staffing issues such as training and	Moderate quality

For peer review only - http://bmjopen.bmj.com/site/about/guidelines.xhtml

No.	First Author (Year) and Country	Sample Size and Age of the participants	Participant Group	Intervention Description	Type of Research, Data Collection and Analysis	Findings of the paper relevant to the review	Quality Appraisal/ Risk of Bias
			healthcare workers and administrative staff			competing workload were noted as drawbacks to the program	
10.	Neale et al (2014) United Kingdom(33)	Service users - N= 30, 23- 62 years<br>Service providers – N= 15, age not mentioned	Homeless individuals with substance use and mentors such as substance use workers, substance use managers and hostel staff	Computer assisted therapies using 20 different psychosocial intervention strategies to identify and reduce substance use based in hostels and homeless shelters (“Breaking Free Online”)	Qualitative- Interviews, Inductive coding and Framework approach	‘Program features’, ‘mentor support’, ‘participant characteristics’ and ‘delivery context’ were noted as factors that lead to successful delivery.	Moderate quality
11.	Paisi et al (2020) United Kingdom (32)	Service users – N= 11, 20 -65 years<br>Service providers – N= 11, age not mentioned	Homeless individuals and the dental clinic staff members, support workers and volunteers.	Community dental clinic that provides both regular and emergency treatments.	Qualitative – semi structured interviews, reflective thematic analysis	Flexibility and the relationship between the patient and dental provider were highlighted as important features.	High quality
12.	Pauly et al (2020) Canada(38)	N= 14, 29-61 years	Homeless with illicit alcohol use	Non-residential community managed alcohol program which provides harm reduction strategies and peer support (“Canadian Managed Alcohol Program Study”)	Qualitative- Semi- structured interviews, inductive coding and constant comparative analysis	Peer led program was successful as it facilitates capacity building, engagement, and empowerment	Moderate quality
13.	Pratt et al (2019) United States of America(40)	N= 40, 29-69 years	Homeless with smoking and alcohol use	Nicotine replacement therapy and motivational interviewing/cognitive behavioural therapy to reduce smoking and alcohol use among the homeless (“Power To Quit 2”)	Qualitative- Interviews, social constructivist approach to grounded theory	Social (peer groups) and environmental (housing etc) factors impact cessation in homeless smokers	High quality
14.	Pratt et al (2022) United States of America (41)	N= 40, 29-70 years	Homeless with smoking and alcohol use	Nicotine replacement therapy and motivational interviewing/cognitive behavioural therapy to reduce smoking and alcohol use among the homeless (“Power To Quit 2”)	Qualitative – Semi structured interviews, social constructivist approach to grounded theory	Social pressure and shelter environment impact the intervention but the integrated treatment along with emotional support from the staff make it beneficial.	High quality
15.	Rash et al (2017) United States of America(39)	N= 355, mean age = 37 years	Homeless with substance use	Behavioural intervention contingency management with the use of incentives such as vouchers and prizes delivered at local community clinics	Quantitative-Adaptation of the Service Utilization Form, Multivariate analysis of variance.	Retention was higher in groups that accessed the intervention compared to the standard arm of care	Moderate/fair quality
16.	Rodriguez et al (2019) United Kingdom(28)	Service users - N= 13, 18- 22 years<br>Service providers – N= 5, age not mentioned	Young homeless people and NGO practitioners	Pedagogical workshops about oral health, mental health, substance misuse, diet etc to increase engagement and awareness	Qualitative- Unstructured interviews and workshops, content analysis	Involvement of young people in co-designing an intervention facilitates engagement, trust building and increases health literacy	High quality
17.	Stormon et al (2018) Australia(30)	N= 76, 41-60 years<br>Feedback – N=24	Disadvantaged adults (clients of community organizations that utilize housing, employment and food services)	Facilitated access pathway between homeless organizations and public dental services. Improving oral health by assessing dental needs, offering dental advice and dental appointments	Quantitative-Questionnaire, Descriptive analysis, and Framework approach	Positive feedback by participants facilitated by the environment, clinical staff and flexibility. Attendance rates varied across the site but was generally high.	Fair/ moderate quality

For peer review only - http://bmjopen.bmj.com/site/about/guidelines.xhtml

SMD groups in the studies found in this review included young adults, single mothers, veterans, and adults with co-occurring conditions of severe mental illness. Based on the information reported in the studies, most of the interventions were focused on adults who were experiencing homelessness and substance use issues,(33, 34, 35, 36, 37, 38, 39, 40) but did not explicitly report on whether they included those who had repeated involvement with the criminal justice system.

Quality appraisal

Of the twelve articles reporting qualitative findings, two were low quality due to lack of detailed findings and methodology not being reported adequately,(31, 36) five moderate quality,(26, 29, 33, 37, 38) due to reporting bias and five high quality.(25, 28, 32, 40, 41) The risk of bias was assessed for the five articles reporting quantitative findings; among the two RCTs: one had a high risk of bias because of attrition and reporting bias,(34) and the other article had a low risk of bias,(35) the remaining three cross-sectional studies were of moderate quality.(27, 30, 39) The findings reported in this review are mostly from high or moderate quality articles, with the inclusion of insights from low quality articles employed strategically for completeness of reporting evidence available and to supplement findings from the adequately reported articles.

Synthesis of qualitative findings

Table 3 presents the themes, subthemes, codes, and quotes from individuals experiencing SMD and frontline staff and stakeholders.

For peer review only

For peer review only - http://bmjopen.bmj.com/site/about/guidelines.xhtml

Table 3: *Themes and subthemes from qualitative synthesis of findings along with the relevant codes and quotes*

Themes	Subthemes	Codes	Quote(s) – people with SMD; or frontline staff
Intervention settings	Physical settings	Housing stability, privacy, confidentiality	*"This is kind of a stressful situation. People are homeless, being at the bottom of their luck, and—boom—and everything. So this is stress. What do you do? You drink, and you smoke, and that's all that you can do, walking around here all day. Do you understand?"(person with SMD) (40)*<br><br>*"But at the same time the addictions piece, especially in terms of stability, I've noticed a lot of the guys that because they are stable in our home, they may make the choice more often to say 'I don't feel like drinking tonight,' so they don't. They don't have to get intoxicated to go to sleep in a shelter on a mat, they can choose not to drink and sometimes they do make that choice not to drink and just watch TV for the evening."(frontline staff) (36)*<br><br>*"If you went in and tried to do anything, people were behind you, over your shoulder, 'what are you doing there' And, you know, I didn't what to discuss with people what I was doing, because they'd take the mick"(person with SMD) (33)*
	Psychological aspects of settings	Communication, trust building, familiarity, mentorship, community, peer pressure, guidance, support, safe space	*"It's not just about dental treatment, I think for a lot of people there is the fear of the dentist because when they do go, it's because they need work done and they're in pain, therefore they associate pain with the dentist."(frontline staff) (29)*<br><br>*"She's not somebody that normally expresses much in a group, she's quite a private person, so I thought it took quite a lot for her to open up, to trust, but I also appreciate the fact that she felt she was in a really good space that she could share that experience with the others and I felt that was really valuable for the rest of the group to hear that. I think this activity [the workshops] encourages people to talk about their own experiences"(frontline staff)(28)*<br><br>*"Yes. A lot better off because… I'm not like, like when I'm here and I'm here with people that are drinking on programs like this and stuff like that I've noticed we're all on the same level. We don't care about the issues or problems, we just, you know, pitch together and do what we gotta do to get ourselves fixed and then from there if we can help other people, and people help other people…"(person with SMD) (38)*<br><br>*"If there is nobody there and you're just left to get on with it, it's quite easy to skip things…I will just put that answer down, you know. But then when you know somebody is there and they are there for that specific reason, then it's a lot easier to go through with things."(person with SMD) (33)*
	Accessibility	Point of contact, space, geography	*"We were put in the medical room along the corridor from the office, but there was no opportunity for practitioner 4 to approach any of the residents. We only saw service users if they specifically wanted to talk about their oral health or if they had walker past the room and wanted to see who we were." (frontline staff) (26)*<br><br>*"I'm from a very rural area, and we don't really have any homelessness centres." (person with SMD) (25)*
Intervention delivery	Improved Awareness	Understandable, ideas, learning from one and another	*"Take things that people say and take it on board, and everything's a learning curve, you learn things all the time... And I'd recommend that to anybody else who is homeless, just listen to other people, take on board what they've got to say, and accept the help that's around you like the group activity [the workshops]".(person with SMD) (28)*<br><br>*"Especially when it was to do with what alcohol can do and what substances can do, I don't think they realized how that affects their oral health, their ears pricked up when you said that" (frontline staff) (29)*

For peer review only - http://bmjopen.bmj.com/site/about/guidelines.xhtml

Themes	Subthemes	Codes	Quote(s) – people with SMD; or frontline staff
	Resources	Workloads, stress, competing needs, volunteers, equipment, funds	*"I think he [client] felt that maybe I would have to sit with him again and, I don't know, maybe I should have sat him down and had a talk with him and I just haven't been able to"(frontline staff) (33)*<br><br>*"You feel like you're spinning so many plates, that you just can't possibly keep them all up in the air" (frontline staff) (25)*<br><br>*"we need to attract funding … it's very difficult to encourage NHS England to commission outside of their routine, the existing contract doesn't favour patients with high treatment needs so we would need them to step outside of their comfort zone and commission something slightly different to what they're used to"(frontline staff) (32)*
	Perceived risks while working with a vulnerable population	Safety, unpredictable, inappropriate behavior, challenges, relevant experience, confident, challenging behaviors	*"Practitioner 1 is confident and appears quite fearless, putting up with language/behaviour that would not be tolerated in a normal clinic." (researcher observation notes of frontline staff) (26)*<br><br>*"Initially we were thinking 'oh we need to make sure that we're not alone in the surgery at any point', and we had a panic alarm and things, we still have all that in place, but it's actually been fine."(frontline staff) (32)*
Ways to enhance engagement and participation	Interest and motivation	Complexity, fears, initiative, specific and complex needs, mixed opinions	*"[Mentor] came in and said 'I'm going home, have you done much?' And I said, 'I couldn't get back on, you know'. And she just took it [the laptop]. I don't know if she was fed up with me or whatever, but she never spoke about it again and I never mentioned it again."(person with SMD) (33)*<br><br>*"The oral health team do not seem bothered to recruit any patients, even if that means sitting waiting with nothing to do—the feeling seems to be that if a patient wants to be seen then they will come to the MDU."(researcher observation notes of frontline staff) (26)*<br><br>*"My goal is to quit within a month or two months. I talked to a couple of people. 'It ain't going to happen.' I said, 'well if you set your mind to certain things, you can do this."(person with SMD) (40)*<br><br>*"I think it's good. It made me feel like I had something to do or like I had a purpose. You know what I mean, not a purpose but it wasn't like the homeless"(41)*
	Adapt to specific circumstances	Context, tailored to the needs of the individual, personalized care	*"People getting through the door, they might not have a roof, might not have any money, might have major drug and alcohol issues, might be threatened with violence, the last thing they want to talk about is their teeth."(frontline staff) (29)*<br><br>*"'we call these people chaotic and that's a bit judgmental, they are actually setting priorities, they've got so much going on in their lives that it [oral health] just falls of their list of priorities, they're saying 'it's my priority to find somewhere to sleep tonight' … The time that you catch people' was therefore identified as 'really important'."(frontline staff)* (32)
	Constant support	Long term care, advice, support	*"About three or four in the morning and I feel like upset then…I can come down and use the program, which is quite good because that way I can put stuff that is all jumbled up in my head down in a way that makes sense and it kind of makes you see that things aren't quite so bad as they seem."(person with SMD) (33)*<br><br>*"At the stage of having goals, an action plan and were working through that . . . but for some homeless people who are nowhere ready, you can make an average of seven appointments before they will turn up once, it's just where your client is at."(frontline staff) (29)*

For peer review only - http://bmjopen.bmj.com/site/about/guidelines.xhtml

Synthesis of twelve papers with qualitative findings,(25, 26, 28, 29, 31, 32, 33, 36, 37, 38, 40, 41) identified three overarching themes in relation to the aims of this review. The three themes are: 1) Intervention settings, 2) Intervention delivery and 3) ways to enhance engagement and participation.

Theme 1: Intervention settings

Eleven papers identified issues related to the settings of interventions which can play a role in the delivery of interventions targeting oral health, substance use and smoking.(25, 26, 27, 28, 32, 33, 36, 37, 38, 40, 41)

Physical settings

Physical settings involved the environment in which the intervention took place. The wider physical environment has been found to have an impact on the intervention experience,(41) with privacy being the key factor for improving physical settings.(32, 33, 41) Communal homeless shelters and busy teaching hospitals lack the space and privacy to deliver interventions involving discussions about difficult and sensitive topics.(32, 33, 41) Contrastingly, stable housing with the necessary privacy allowed people experiencing SMD to focus on their recovery journey, whilst also creating a space in which residents could spend time away from peers who were sometimes perceived as having a negative peer group influence.(36, 40)

Psychological aspects of settings

Psychological aspects related to the less visible parts of the interventions were identified across ten papers.(25, 26, 28, 29, 32, 33, 37, 38, 40, 41) Firstly, it was reported that relationships between people experiencing SMD and service providers played a vital part in the delivery of interventions. Through good communication,(26, 28, 32, 41) trust building,(28, 29, 32, 41) familiarity of working with a vulnerable population,(25, 32) and mentorship,(33, 37) interventions were able to form a 'safe and respectable environment'.(28, 32, 37, 41) Secondly, papers discussed the importance of peer support as a way of increasing the effectiveness of interventions.(32, 37, 38) There were also reports of the impact negative peer influence could have on the recovery process. For example, smoking and drinking were linked to socializing with others, which could increase the urge to smoke or drink.(40, 41)

Accessibility

Accessibility of interventions was one of the factors found to be important related to implementation of interventions among people experiencing SMD.(25, 26, 32, 41) Firstly, accessible

For peer review only - http://bmjopen.bmj.com/site/about/guidelines.xhtml

and spacious meeting points within the services were reported to help with their participation in the intervention, especially in the case of oral health interventions that were delivered either in a community setup (open space) or a mobile dental van .(26) Secondly, geographical proximity could act as a barrier as rural and remote areas lack the facilities and resources, which could influence the access of people experiencing SMD.(25, 32) Lastly, it was reported that access could become an issue when service users move to more stable housing as weather conditions, distance, work and other appointments tend to make it challenging to attend the intervention sessions.(41)

Theme 2: Intervention delivery

Nine papers discussed aspects such as information availability, resources and perceived risks of working with a vulnerable population that could be important for roll out and delivery of interventions addressing oral health, smoking and substance use.(25, 26, 28, 29, 31, 32, 33, 37, 41)

Improved Awareness

Awareness and information availability were discussed in papers focusing on improving oral health, smoking and alcohol use.(28, 29, 32, 41) Sharing information between service providers and SMD groups was identified as an important issue across the papers as it created opportunities to promote involvement and behavior change.(28, 29, 41) It was reported that easily understandable information encouraged people experiencing SMD to view healthier behaviors as important (e.g. tooth brushing) and helped to signpost them to necessary services.(28, 41) Clear and simple explanations of treatment options available was seen to help them in decision-making.(32) Service providers also felt that they learned more about healthy behaviors and were able to pass their newly gained knowledge to their clients.(29)

Resources

Five papers discussed the importance of having necessary resources to enable interventions to run efficiently and effectively.(25, 31, 32, 33, 37) The majority of these highlighted the importance of distribution of workloads among staff because of difficulties in implementing interventions with competing duties and work within the organizations.(25, 33, 37) Funding and resources such as volunteers and materials were identified in oral health interventions as an important issue that impacts implementation and long-term sustainability.(31, 32)

Perceived risks working with a vulnerable population

Papers reported on the perceived risks of delivering interventions to vulnerable populations as challenging at times by service providers.(25, 26, 32) There were concerns about safety of service

For peer review only

For peer review only - http://bmjopen.bmj.com/site/about/guidelines.xhtml

providers while interacting with clients who were seen to be “unpredictable”. The need for training and being better equipped to work in this environment and setting boundaries between service providers and clients was repeatedly mentioned by service providers.(25, 26, 32, 37) The papers also highlighted the importance of training opportunities that provide service providers with the necessary skills to handle volatile and difficult situations.(25, 37)

Theme 3: Ways to enhance participation and engagement

Ten papers identified factors such as interest and motivation levels, adaptability, and long-term support that could help to improve outcomes and create sustainable interventions by enhancing engagement and participation.(25, 26, 28, 29, 31, 32, 33, 37, 40, 41)

Interest and motivation

Nine papers highlighted that the interest and motivation levels of both staff supporting SMD groups and people experiencing SMD play an important role in the implementation of interventions. Disinterest was sometimes observed amongst service providers, due to concerns about the complexity of delivering the intervention,(25, 29, 31) lack of engagement with third sector organizations,(26) poor uptake of the intervention by the target populations,(25, 29, 31) and preconceived notions of improper behaviour by SMD groups.(41) Interestingly, interventions were met with similar feelings of indifference by people experiencing SMD if the intervention did not address their specific and complex needs such as housing and financial problems.(25, 26, 32) Two papers on oral health interventions found that younger adults and families with children were more eager to engage compared to single men.(28, 29) Papers discussing the same smoking intervention illustrated that an awareness of health benefits and risks played a part in motivating people in engaging with the intervention.(40, 41)

Adapting to specific circumstances

Adaptability of interventions was noted as an essential feature among four papers.(29, 32, 33, 40) Tailoring the interventions to address their specific needs at the time such as housing and employment was noted to increase participation and better outcomes.(29, 40) Service users of a community dental service also suggested flexible and longer dental appointments would be helpful and in the long-term these adaptions would help reduce missed appointments.(32) Another paper reported that people experiencing SMD were keen to have more face to face interactions rather than digital, which highlights the drive to more personalized care.(33)

Long term support

For peer review only

Four papers identified sustained and long-term support as a factor that could contribute towards better intervention outcomes.(29, 32, 33, 37) Service providers expressed a need for interventions which allowed people experiencing SMD to continue with services/programmes despite missing appointments or not completing treatment within the required delivery timeframe especially because of the transitionary nature of SMD groups.(32, 33) Similarly, for a substance reduction intervention, a preference for a long-term intervention, that allowed and supported them to gradually integrate into their new stage of their lives.(33, 37) Two papers on oral health interventions suggested that drop-in services offered flexibility in seeking advice or seeing a practitioner and helped to reduce anxiety surrounding accessing treatment for dental health.(29, 32)

Synthesis of quantitative findings related to retention and implementation

Four papers reported quantitative findings on retention and program attendance,(30, 34, 35, 39) as indicators of uptake and sustained implementation of interventions.

Three papers on substance use interventions reported high levels of retention in their intervention groups.(34, 35, 39) Two studies among them delivered the interventions along with housing services but the findings were mixed and limited on whether retention was significantly associated with the housing services or not. (34, 35, 39)

There was no difference in the attendance levels in the studies related to substance use interventions.(34, 35) The attendance level for an oral health promotion intervention delivered in community settings was high (85%), however it varied across community centers and was dependent on timing of appointment and dental treatments offered. More non-attendance seen for afternoon appointments and complex dental treatments (e.g. surgical and prosthodontic treatments).(30)

Additionally, workplace beliefs and practices amongst service providers such as knowledge, intention and goals, were reported to influence implementation behaviors.(27)

DISCUSSION

This review synthesized different factors that could influence the implementation and sustainability of interventions related to improving oral health and related health behaviors of people experiencing SMD. Evidence suggested that psychological aspects of intervention settings such as building trust and communication form an integral part in the creating a safe environment and that these are just as essential as the structural components of settings such as physical environment. Review findings further suggest that adequate staff capacity, funding and equipment would ease the delivery of interventions by reducing the immense pressure faced by service providers supporting

For peer review only - http://bmjopen.bmj.com/site/about/guidelines.xhtml

the interventions. It was also suggested that implementation is dependent on the interest and motivation of not only people experiencing SMD but also on that of service providers in delivering difficult and complex interventions.

Most of the included studies were related to oral health and substance use (drug and alcohol). There was a lack of evidence on diet and smoking interventions among this population. Previous evidence has shown that tobacco use and poor diet, often due to limited choice available while experiencing homelessness and related disadvantages, result in a range of adverse short term (nutritional deficiencies) and long term health outcomes (cancer, diabetes, heart disease).(42, 43, 44) Food insecurity is often linked to elevated tobacco use, mental health issues and an increased risk of substance misuse.(45, 46, 47)

While most of the papers mainly focused on the perspectives of people experiencing SMD, the limited data from service providers brought to light some of the challenges faced during implementation. This supports the notion that intervention implementation needs the co-ordination and collective effort of everyone involved. All the interventions included were designed focussing on service provision,(25, 26, 27, 28, 30, 31, 32, 33, 34, 35, 37, 38, 39, 40, 41, 48) except for one study which focussed on a training intervention for service providers (29).Limited evidence was available on the long-term sustainability of interventions, which highlights another evidence gap that needs to be addressed.

Our review findings suggest that the retention in interventions may depend on the type of treatment offered, which at times can be influenced by the availability of housing provision. Timing and type of treatment may also influence attendance rates; for instance, morning appointments might be more beneficial, especially for individuals struggling with alcohol addiction, as they may be less intoxicated compared to later in the day. Our review findings also complement our systematic review about the effectiveness of interventions that improve oral health and related health behaviors in SMD groups – the effectiveness review found that interventions that integrated health with the individual's wider needs (for e.g. housing, employment, mental health) were more effective than usual care.(49) The findings we have are very limited regarding retention and attendance, more effort needs to be taken to understand how to improve reach and retention among SMD groups so that they can access and use the interventions efficiently.

A systematic review on access to dental care among individuals experiencing homelessness in the UK identified similar findings around awareness, accessibility and organizational issues (lack of financial resources and collaboration between sectors) having an influence on implementation.(50) This was also similarly identified in another review on smoking cessation among homeless populations in

For peer review only - http://bmjopen.bmj.com/site/about/guidelines.xhtml

For peer review only

high-income countries.(51) The importance of continued engagement in services was highlighted in a review on substance use support for young people (ages 12 to 24) experiencing homelessness, which was also reflected in our findings.(52) Existing literature on interventions targeting health conditions such as HIV and Hepatitis C in this population have shown that improved health outcomes are linked to increased awareness, establishment of positive relationships with service providers and integrated treatment involving other health behaviours.(53, 54, 55)

Some findings from our review on aspects related to intervention settings, and intervention delivery aligned with CFIR constructs of Inner and Outer settings domains.(14) Subthemes in our findings on ways to enhance engagement, aligns with both Individuals and Implementation process domains.(14) The use of CFIR framework helps us understand the impact of intervention settings, delivery methods and engagement on the implementation process. It also provides a comprehensive approach for guiding the development of interventions targeting SMD groups and improving their efficacy in practical settings.

Strengths and Limitations

This systematic review is novel in that it assesses the implementation and sustainability of interventions on oral health together with co-occurring and related health behaviors in people experiencing SMD. It addresses an evidence gap on interventions targeting these health challenges and identifies ways to overcome implementation issues faced by these specific interventions. Another strength of this review lies in its comprehensive search strategy and use of a published tool (i.e. CFIR) to make sense of the results. It also highlights gaps in the evidence base on interventions related to diet, as well as studies that include repeat offenders. However, the confidence in the evidence from this review is limited as most of the papers were of moderate quality. Studies lacked detailed data collection methods and standardized evaluations which influenced their quality. Another limitation of this work is that intersectionality was not considered explicitly during the analysis of the data. Furthermore, the findings may not be generalizable to all contexts since the included papers were from high-income countries.

Implications

These findings offer valuable insights for enhancing existing interventions by paying attention to settings, delivery, and engagement opportunities. Evidence from this review points to the need for additional research on interventions targeting smoking and diet. These areas hold significant value due to their direct links with general and oral health. It is also important for interventions to address not only individual behaviours but also overlapping behaviours of substance use, smoking and poor

For peer review only - http://bmjopen.bmj.com/site/about/guidelines.xhtml

diet. This could help reduce the strain on resources and improve engagement. Furthermore, higher quality research that focuses more on sustainability and intersectionality is warranted to further investigate and refine interventions focused on SMD groups.

Author Contributions

Conceptualization: SER, EK, EAA, CB, RGW, ECJ, LJM, FB, DC, DL, MP, FFS; methodology: SER, DAJ, LJM, EAA, ECJ, CR, EK, DC, FB; draft preparation: SER, DAJ, EAA; writing - review and editing all; funding acquisition: SER, EK, FB, CB, RGW, DC, DL, MP, FFS.

Ethical approval

None sought.

Data Availability Statement

No data are available.

Funding Statement

This project is funded by the National Institute for Health and Care Research (NIHR) Policy Research Programme (NIHR200415). EAA was supported by the National Institute for Health and Care Research (NIHR) School for Public Health Research (SPHR) Pre-doctoral Fellowship, Grant Reference Number PD-SPH-2015. EK is supported by an NIHR Senior Investigator award and directs the NIHR funded Applied Research Collaboration North East and North Cumbria.

Conflicts of Interest

The authors declare no conflict of interest. The funders had no role in the design of the study; in the collection, analyses, or interpretation of data; in the writing of the manuscript, or in the decision to publish the results.

Word Count

3329 words

Figure 1*: PRISMA Flowchart for the search results.*

References:

For peer review only - http://bmjopen.bmj.com/site/about/guidelines.xhtml

1. Bramley G, Fitzpatrick S, Edwards J, et al. Hard edges: mapping severe and multiple disadvantage in England. 2015.
2. Sandu RD. Defining severe and multiple disadvantage from the inside: Perspectives of young people and of their support workers. Journal of Community Psychology. 2021;49(5):1470-86.
3. Rae BE, Rees S. The perceptions of homeless people regarding their healthcare needs and experiences of receiving health care. Journal of Advanced Nursing. 2015;71(9):2096-107.
4. Conte M, Broder HL, Jenkins G, et al. Oral Health, Related Behaviors and Oral Health Impacts Among Homeless Adults. J Public Health Dent. 2006;66(4):276-8.
5. Hertzberg D, Boobis S. Unhealthy State of Homelessness 2022: Findings from the Homeless Health Needs Audit. 2022.
6. Groundswell. Healthy Mouths: A peer-led health audit on the oral health of people experiencing homelessness. London Groundswell; 2017.
7. Evans NS, Dowler EA. Food, health and eating among single homeless and marginalized people in London. Journal of Human Nutrition and Dietetics. 1999;12(3):179-99.
8. Sheiham A. Oral health, general health and quality of life. SciELO Public Health; 2005. p. 644-.
9. Wiecha JL, Dwyer JT, Dunn-Strohecker M. Nutrition and health services needs among the homeless. Public Health Rep. 1991;106(4):364-74.
10. Beaton L, Humphris G, Rodriguez A,et al. Community-based oral health interventions for people experiencing homelessness: a scoping review. Community Dent Health. 2020;37(2):150-60.
11. Hwang SW, Tolomiczenko G, Kouyoumdjian FG, et al. Interventions to improve the health of the homeless: a systematic review. American journal of preventive medicine. 2005;29(4):311-. e75.
12. Speirs V, Johnson M, Jirojwong S. A systematic review of interventions for homeless women. Journal of Clinical Nursing. 2013;22(7-8):1080-93.
13. Damschroder LJ, Aron DC, Keith RE, et al. Fostering implementation of health services research findings into practice: a consolidated framework for advancing implementation science. Implementation Science. 2009;4(1):50.
14. Damschroder LJ, Reardon CM, Widerquist MAO,et al . The updated Consolidated Framework for Implementation Research based on user feedback. Implementation Science. 2022;17(1):75.
15. Shelton RC, Cooper BR, Stirman SW. The Sustainability of Evidence-Based Interventions and Practices in Public Health and Health Care. Annual Review of Public Health. 2018;39(1):55-76.
16. McGowan L, Joyes E, Adams E, et al. Systematic review of oral health and related health behaviour interventions in adults with severe and multiple disadvantages. PROSPERO. 2020.
17. McGowan LJ, Joyes EC, Adams EA, et al. Investigating the Effectiveness and Acceptability of Oral Health and Related Health Behaviour Interventions in Adults with Severe and Multiple Disadvantage: Protocol for a Mixed-Methods Systematic Review. Int J Environ Res Public Health. 2021;18(21).
18. Page MJ, Moher D, Bossuyt PM, et al. PRISMA 2020 explanation and elaboration: updated guidance and exemplars for reporting systematic reviews. BMJ. 2021;372:n160.

For peer review only - http://bmjopen.bmj.com/site/about/guidelines.xhtml

19. Covidence. Covidence Systematic Review Software.
20. Programme. CAS. CASP Qualitative Checklist 2022 [Available from: https://casp-uk.net/casp-tools-checklists.
21. Higgins JP, Thomas J, Chandler J, et al. Cochrane handbook for systematic reviews of interventions: John Wiley & Sons; 2019.
22. National Heart LaBI. Study quality assessment tool for observational and cross sectional studies 2021 [Available from: https://www.nhlbi.nih.gov/health-topics/study-quality-assessment-tools.
23. Butler A, Hall H, Copnell B. A Guide to Writing a Qualitative Systematic Review Protocol to Enhance Evidence-Based Practice in Nursing and Health Care. Worldviews Evid Based Nurs. 2016;13(3):241-9.
24. Saini M, Shlonsky A. Systematic synthesis of qualitative research: OUP USA; 2012.
25. Beaton L, Freeman R. Oral health promotion and homelessness: A theory-based approach to understanding processes of implementation and adoption. Health Education Journal. 2016;75(2):184-97.
26. Beaton L, Anderson I, Humphris G, et al. Implementing an Oral Health Intervention for People Experiencing Homelessness in Scotland: A Participant Observation Study. Dent J (Basel). 2018;6(4).
27. Beaton L, Humphris G, Rodriguez A, et al. Implementing the Smile4life intervention for people experiencing homelessness: a path analytical evaluation. BMC Oral Health. 2021;21(1):383.
28. Rodriguez A, Beaton L, Freeman R. Strengthening Social Interactions and Constructing New Oral Health and Health Knowledge: The Co-design, Implementation and Evaluation of A Pedagogical Workshop Program with and for Homeless Young People. Dent J (Basel). 2019;7(1).
29. Coles E, Watt C, Freeman R. 'Something to Smile About': An evaluation of a capacity-building oral health intervention for staff working with homeless people. Health Education Journal. 2012;72(2):146-55.
30. Stormon N, Pradhan A, McAuliffe A, et al. Does a facilitated pathway improve access to dental services for homeless and disadvantaged adults? Evaluation and program planning. 2018;71:46-50.
31. Doughty J, Johnson A, Tatari A, et al. A pilot denture service collaboration between Den-Tech denture charity and Crisis at Christmas Dental Service (CCDS): A Plan-Do-Study-Act analysis. British Dental Journal. 2020;228(3):183-90.
32. Paisi M, Baines R, Worle C, et al . Evaluation of a community dental clinic providing care to people experiencing homelessness: A mixed methods approach. Health Expectations. 2020;23(5):1289-99.
33. Neale J, Stevenson C. Positive and negative features of a computer assisted drug treatment program delivered by mentors to homeless drug users living in hostels. Journal of substance abuse treatment. 2014;47(4):258-64.
34. Burnam MA, Morton SC, McGlynn EA, et al. An experimental evaluation of residential and nonresidential treatment for dually diagnosed homeless adults. J Addict Dis. 1995;14(4):111-34.
35. Collins SE, Clifasefi SL, Nelson LA, et al. Randomized controlled trial of harm reduction treatment for alcohol (HaRT-A) for people experiencing homelessness and alcohol use disorder. Int J Drug Policy. 2019;67:24-33.

For peer review only - http://bmjopen.bmj.com/site/about/guidelines.xhtml

36. Forchuk C, Richardson J, Atyeo H, et al. Qualitative findings from a Housing First evaluation project for homeless Veterans in Canada. Journal of Military, Veteran and Family Health. 2022;8(2):27-38.
37. Henderson PM. Program evaluation of a long-term substance abuse treatment program for homeless men: Spalding University; 2003.
38. Pauly B, King V, Smith A, et al. Breaking the cycle of survival drinking: insights from a non-residential, peer-initiated and peer-run managed alcohol program. Drugs: Education, Prevention and Policy. 2021;28(2):172-80.
39. Rash CJ, Alessi SM, Petry NM. Substance abuse treatment patients in housing programs respond to contingency management interventions. J Subst Abuse Treat. 2017;72:97-102.
40. Pratt R, Pernat C, Kerandi L, et al. "It'sa hard thing to manage when you're homeless": the impact of the social environment on smoking cessation for smokers experiencing homelessness. BMC Public Health. 2019;19(1):1-8.
41. Pratt R, Xiong S, Kmiecik A, et al. The implementation of a smoking cessation and alcohol abstinence intervention for people experiencing homelessness. BMC public health. 2022;22(1):1260.
42. Baggett TP, Singer DE, Rao SR, et al . Food Insufficiency and Health Services Utilization in a National Sample of Homeless Adults. Journal of General Internal Medicine. 2011;26(6):627-34.
43. Fallaize R, Seale JV, Mortin C, et al. Dietary intake, nutritional status and mental wellbeing of homeless adults in Reading, UK. British Journal of Nutrition. 2017;118(9):707-14.
44. Okuyemi KS, Caldwell AR, Thomas JL, et al. Homelessness and Smoking Cessation: Insights from Focus Groups. Nicotine Tob Res. 2006;8(2):287-96.
45. Langnäse K, Müller MJ. Nutrition and health in an adult urban homeless population in Germany. Public Health Nutrition. 2001;4(3):805-11.
46. Knudsen HK. Implementation of smoking cessation treatment in substance use disorder treatment settings: a review. The American Journal of Drug and Alcohol Abuse. 2017;43(2):215-25.
47. Kim JE, Flentje A, Tsoh JY, et al . Cigarette Smoking among Women Who Are Homeless or Unstably Housed: Examining the Role of Food Insecurity. J Urban Health. 2017;94(4):514-24.
48. Beaton L, Anderson I, Humphris G, et al. Implementing an Oral Health Intervention for People Experiencing Homelessness in Scotland: A Participant Observation Study. Dentistry Journal. 2018;6(4):68.
49. McGowan LJ, John DA, Kenny RPW, et al . Interventions to improve oral health and related health behaviours of substance use, smoking and diet in people with severe and multiple disadvantage: a systematic review of effectiveness and cost-effectiveness. 2023.
50. Paisi M, Kay E, Plessas A, et al. Barriers and enablers to accessing dental services for people experiencing homelessness: A systematic review. Community dentistry and oral epidemiology. 2019;47(2):103-11.
51. Soar K, Dawkins L, Robson D, et al. Smoking amongst adults experiencing homelessness: a systematic review of prevalence rates, interventions and the barriers and facilitators to quitting and staying quit. Journal of Smoking Cessation. 2020;15(2):94-108.
52. Xiang X. A review of interventions for substance use among homeless youth. Research on Social Work Practice. 2013;23(1):34-45.

For peer review only - http://bmjopen.bmj.com/site/about/guidelines.xhtml

53. Nyamathi A, Kennedy B, Branson C, et al. Impact of Nursing Intervention on Improving HIV, Hepatitis Knowledge and Mental Health Among Homeless Young Adults. Community Mental Health Journal. 2013;49(2):178-84.
54. Young SD, Rice E. Online social networking technologies, HIV knowledge, and sexual risk and testing behaviors among homeless youth. AIDS and Behavior. 2011;15:253-60.
55. Slesnick N, Kang MJ. The impact of an integrated treatment on HIV risk behavior among homeless youth: a randomized controlled trial. Journal of behavioral medicine. 2008;31:45-59.

For peer review only

For peer review only - http://bmjopen.bmj.com/site/about/guidelines.xhtml

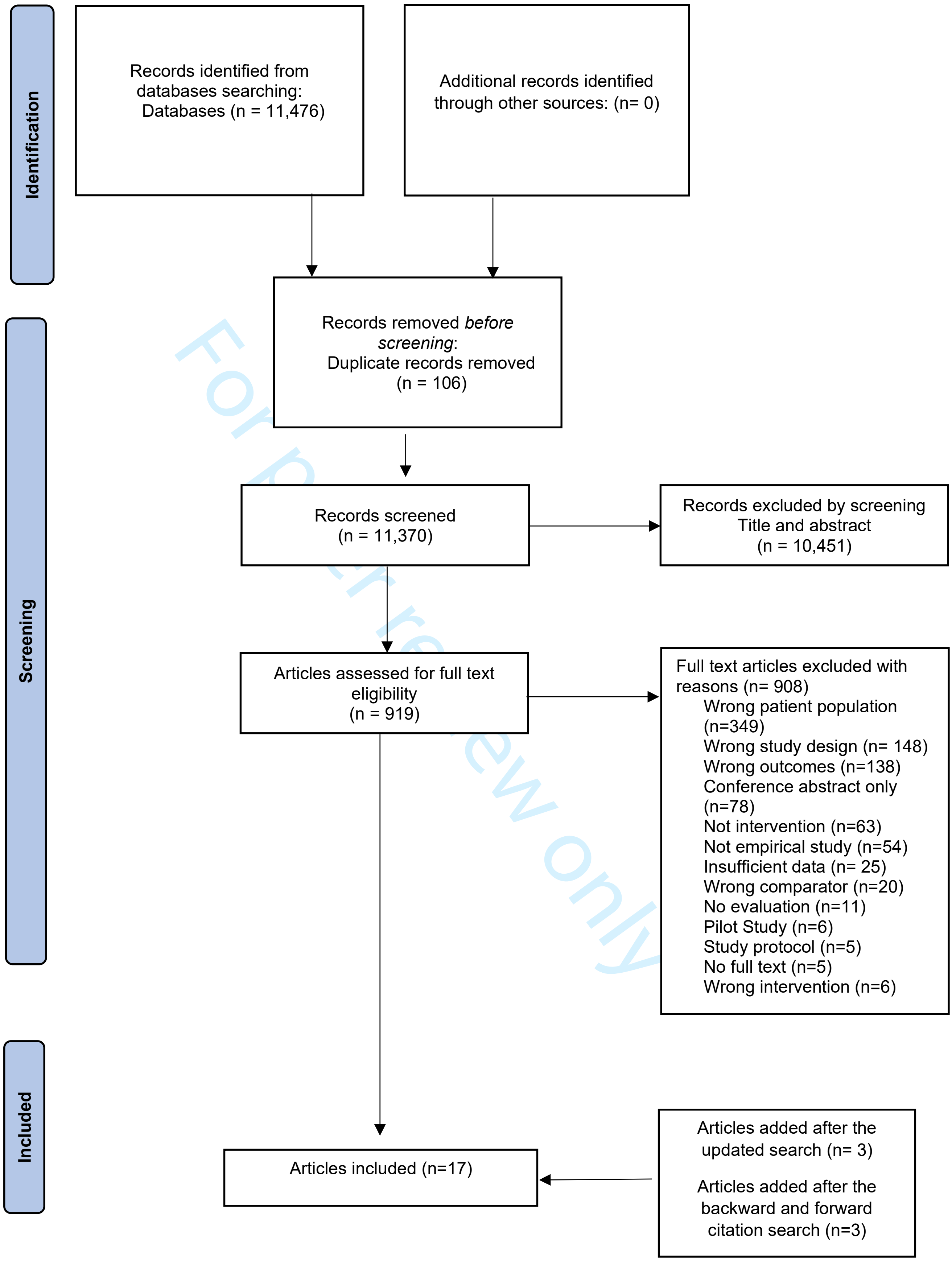

For peer review only

Search Strategy

Database(s): **Ovid MEDLINE(R) and Epub Ahead of Print, In-Process & Other Non-Indexed Citations, Daily and Versions(R)** 1946 to July 14, 2020

Search Strategy:

#	Searches	Results
1	Oral Hygiene/	12867
2	Mouth Rehabilitation/	1441
3	Oral Health/	16483
4	exp Dental Health Services/	38072
5	((dental or oral or tooth or teeth or mouth) adj3 (health or care or hygiene or rehabilitation)).ti,ab,kw.	60448
6	or/1-5	92888
7	Smoking/	139868
8	(smoking or cigarette* or tobacco).ti,ab,kw.	301064
9	exp "Tobacco Use"/	4579
10	Alcohol Drinking/	66885
11	Alcoholism/	74948
12	Alcoholics/	845
13	(alcoholic* adj3 (person or people or adult or parent* or family)).ti,ab,kw.	889
14	"street drink*".ti,ab,kw.	8
15	exp Substance-Related Disorders/	277945
16	exp Drug users/	3139
17	Behavior, Addictive/	9829
18	((alcohol or drug* or substance*) adj2 (misuse* or abuse* or use* or addict* or dependenc* or issue* or problem)).ti,ab,kw.	265053
19	(drug adj1 (habit or tak* or hard or illicit or inject*)).ti,ab,kw.	22853
20	or/7-19	768779
21	((sugar or sucrose or fructose or glucose) adj2 (intake or consum*)).ti,ab,kw.	13634
22	diet*.ti,ab,kw.	569753
23	"sugary foods".ti,ab,kw.	175
24	exp Dietary Sugars/ or Diet/	163140
25	((processed or acidic) adj1 food*).ti,ab,kw.	3659
26	((sugary or fizzy or carbonated or soft) adj1 drink*).ti,ab,kw.	4464
27	carbonated beverages/ or sugar-sweetened beverages/	2986
28	soda.ti,ab,kw.	4168
29	or/21-28	636784
30	or/1-20	856955
31	(severe and multiple disadvantage*).ti,ab,kw.	4
32	Homeless Persons/	7717
33	homeless*.ti,ab,kw.	10721
34	((hous* or home* or accommodat* or shelter) adj3 (insecur* or instability or unstable or stability)).ti,ab,kw.	2832
35	or/32-34	14839
36	(probationer* or parolee* or ((repeat* or ex or re or revolving door or habitual or multiple or former* or previously*) adj1 (offen* or convict* or prisoner* or imprison* or incarcerat* or criminal*)) or (former adj3 inmate*) or ((community or probation* or parole* or reintegrat*) adj4 (prison* or offender* or criminal* or convict* or inmate*)) or ((individuals or men or women) adj2 (probation or parole)) or ((reintegrate* or reent* or return*) adj3 community)).ti,ab,kw.	3929
37	"criminal justice".ti,ab,kw.	4314
38	or/36-37	7771
39	((program* or policy or policies or strateg* or scheme* or project* or initiative* or "care package" or training or educat* or pilot or guidance or guideline* or study or pathway or treatment* or promot* or management or "support group" or process* or trial* or intervention*) adj5 (evaluat* or effect* or measur* or assess* or experiment* or impact* or feasab* or acceptab* or efficacy or perception* or belief* or uptake or consequence* or attitud* or barrier* or facilit* or motivat* or experience* or implement* or adher* or retention or retain* or reduc* or increas* or improv* or outcome* or cost* or benefit* or interview* or qualitative or ethnograph* or "focus group*")).ti,ab,kw.	4377977
40	"housing first".ti,ab,kw.	299
41	(outcome* adj5 evaluat*).ti,ab,kw.	82363

For peer review only

42	or/39-41	4410253
43	(35 or 38) and (6 or 20 or 29)	8276
44	42 and 43	3940

For peer review only

PRISMA 2020 Checklist

Section and Topic	Item #	Checklist item	Location where item is reported
**TITLE**			
Title	1	Identify the report as a systematic review.	Lines 2-3
**ABSTRACT**			
Abstract	2	See the PRISMA 2020 for Abstracts checklist.	Page 1
**INTRODUCTION**			
Rationale	3	Describe the rationale for the review in the context of existing knowledge.	Lines 38 - 44
Objectives	4	Provide an explicit statement of the objective(s) or question(s) the review addresses.	Lines 54- 56
**METHODS**			
Eligibility criteria	5	Specify the inclusion and exclusion criteria for the review and how studies were grouped for the syntheses.	Table 1
Information sources	6	Specify all databases, registers, websites, organisations, reference lists and other sources searched or consulted to identify studies. Specify the date when each source was last searched or consulted.	Lines 63- 68
Search strategy	7	Present the full search strategies for all databases, registers and websites, including any filters and limits used.	Supplementary file
Selection process	8	Specify the methods used to decide whether a study met the inclusion criteria of the review, including how many reviewers screened each record and each report retrieved, whether they worked independently, and if applicable, details of automation tools used in the process.	Lines 70 -73
Data collection process	9	Specify the methods used to collect data from reports, including how many reviewers collected data from each report, whether they worked independently, any processes for obtaining or confirming data from study investigators, and if applicable, details of automation tools used in the process.	Lines 85 - 91
Data items	10a	List and define all outcomes for which data were sought. Specify whether all results that were compatible with each outcome domain in each study were sought (e.g. for all measures, time points, analyses), and if not, the methods used to decide which results to collect.	Table 1
	10b	List and define all other variables for which data were sought (e.g. participant and intervention characteristics, funding sources). Describe any assumptions made about any missing or unclear information.	Table 1 and Table 2
Study risk of bias assessment	11	Specify the methods used to assess risk of bias in the included studies, including details of the tool(s) used, how many reviewers assessed each study and whether they worked independently, and if applicable, details of automation tools used in the process.	Lines 75 - 83
Effect measures	12	Specify for each outcome the effect measure(s) (e.g. risk ratio, mean difference) used in the synthesis or presentation of results.	Table 2
Synthesis methods	13a	Describe the processes used to decide which studies were eligible for each synthesis (e.g. tabulating the study intervention characteristics and comparing against the planned groups for each synthesis (item #5)).	Table 1
	13b	Describe any methods required to prepare the data for presentation or synthesis, such as handling of missing summary statistics, or data conversions.	Lines 85 -91
	13c	Describe any methods used to tabulate or visually display results of individual studies and syntheses.	Lines 85 -91
	13d	Describe any methods used to synthesize results and provide a rationale for the choice(s). If meta-analysis was performed, describe the model(s), method(s) to identify the presence and extent of statistical heterogeneity, and software package(s) used.	Liness 85 -91
	13e	Describe any methods used to explore possible causes of heterogeneity among study results (e.g. subgroup analysis, meta-regression).	N/A
	13f	Describe any sensitivity analyses conducted to assess robustness of the synthesized results.	N/A
Reporting bias assessment	14	Describe any methods used to assess risk of bias due to missing results in a synthesis (arising from reporting biases).	Lines 75 - 83
Certainty	15	Describe any methods used to assess certainty (or confidence) in the body of evidence for an outcome.	Lines 75 - 83

For peer review only

PRISMA 2020 Checklist

Section and Topic	Item #	Checklist item	Location where item is reported
assessment			
**RESULTS**			
Study selection	16a	Describe the results of the search and selection process, from the number of records identified in the search to the number of studies included in the review, ideally using a flow diagram.	Figure 1
	16b	Cite studies that might appear to meet the inclusion criteria, but which were excluded, and explain why they were excluded.	Figure 1
Study characteristics	17	Cite each included study and present its characteristics.	Table 2
Risk of bias in studies	18	Present assessments of risk of bias for each included study.	Table 2
Results of individual studies	19	For all outcomes, present, for each study: (a) summary statistics for each group (where appropriate) and (b) an effect estimate and its precision (e.g. confidence/credible interval), ideally using structured tables or plots.	Table 1
Results of syntheses	20a	For each synthesis, briefly summarise the characteristics and risk of bias among contributing studies.	Lines 102 -108
	20b	Present results of all statistical syntheses conducted. If meta-analysis was done, present for each the summary estimate and its precision (e.g. confidence/credible interval) and measures of statistical heterogeneity. If comparing groups, describe the direction of the effect.	N/A
	20c	Present results of all investigations of possible causes of heterogeneity among study results.	N/A
	20d	Present results of all sensitivity analyses conducted to assess the robustness of the synthesized results.	N/A
Reporting biases	21	Present assessments of risk of bias due to missing results (arising from reporting biases) for each synthesis assessed.	Lines 103 - 108
Certainty of evidence	22	Present assessments of certainty (or confidence) in the body of evidence for each outcome assessed.	N/A
**DISCUSSION**			
Discussion	23a	Provide a general interpretation of the results in the context of other evidence.	Lines 223 -251
	23b	Discuss any limitations of the evidence included in the review.	Lines 274 - 277
	23c	Discuss any limitations of the review processes used.	Lines 277 -278
	23d	Discuss implications of the results for practice, policy, and future research.	Lines 281 -288
**OTHER INFORMATION**			
Registration and protocol	24a	Provide registration information for the review, including register name and registration number, or state that the review was not registered.	Lines 58 -59
	24b	Indicate where the review protocol can be accessed, or state that a protocol was not prepared.	Lines 58 -59
	24c	Describe and explain any amendments to information provided at registration or in the protocol.	
Support	25	Describe sources of financial or non-financial support for the review, and the role of the funders or sponsors in the review.	Lines 295 - 300
Competing interests	26	Declare any competing interests of review authors.	Lines 302-304
Availability of data, code and	27	Report which of the following are publicly available and where they can be found: template data collection forms; data extracted from included studies; data used for all analyses; analytic code; any other materials used in the review.	N/A

For peer review only - http://bmjopen.bmj.com/site/about/guidelines.xhtml

PRISMA 2020 Checklist

Section and Topic	Item #	Checklist item	Location where item is reported
other materials			

From: Page MJ, McKenzie JE, Bossuyt PM, Boutron I, Hoffmann TC, Mulrow CD, et al. The PRISMA 2020 statement: an updated guideline for reporting systematic reviews. BMJ 2021;372:n71. doi: 10.1136/bmj.n71

For more information, visit: http://www.prisma-statement.org/

For peer review only

BMJ Open

Factors influencing implementation and sustainability of interventions to improve oral health and related health behaviours in adults experiencing severe and multiple disadvantage: A mixed-methods systematic review

Journal:	*BMJ Open*
Manuscript ID	bmjopen-2023-080160.R2
Article Type:	Original research
Date Submitted by the Author:	11-Dec-2023
Complete List of Authors:	John , Deepti; Newcastle University Population Health Sciences Institute<br>Adams, Emma; Newcastle University Population Health Sciences Institute<br>McGowan, Laura; Newcastle University Population Health Sciences Institute<br>Joyes, Emma; Newcastle University Population Health Sciences Institute<br>Richmond, Catherine; Newcastle University Population Health Sciences Institute<br>Beyer, Fiona ; Newcastle University Population Health Sciences Institute, Evidence Synthesis Group and Innovation Observatory<br>Landes, David; NHS England and NHS Improvement North East and Yorkshire<br>Watt, Richard; University College London, Epidemiology and Public Health; King's College London, King's College Dental Institute<br>Sniehotta, Falko; Newcastle University Population Health Sciences Institute, NIHR Policy Research Unit Behavioural Science; Heidelberg University Medical Faculty Mannheim, Department of Public Health, Social and Preventive Medicine, Centre for Preventive Medicine and Digital Health (CPD)<br>Paisi, Martha; Plymouth University Peninsula Dental School<br>Bambra, Clare; Newcastle University Population Health Sciences Institute<br>Craig, Dawn; Newcastle University Population Health Sciences Institute<br>Kaner, Eileen; Newcastle University Population Health Sciences Institute<br>Ramsay, Sheena; Newcastle University Population Health Sciences Institute
**Primary Subject Heading**:	Public health
Secondary Subject Heading:	Health policy, Smoking and tobacco, Dentistry and oral medicine
Keywords:	Health Equity, PUBLIC HEALTH, Behavior

SCHOLARONE™
Manuscripts

I, the Submitting Author has the right to grant and does grant on behalf of all authors of the Work (as defined in the below author licence), an exclusive licence and/or a non-exclusive licence for contributions from authors who are: i) UK Crown employees; ii) where BMJ has agreed a CC-BY licence shall apply, and/or iii) in accordance with the terms applicable for US Federal Government officers or employees acting as part of their official duties; on a worldwide, perpetual, irrevocable, royalty-free basis to BMJ Publishing Group Ltd ("BMJ") its licensees and where the relevant Journal is co-owned by BMJ to the co-owners of the Journal, to publish the Work in this journal and any other BMJ products and to exploit all rights, as set out in our licence.

The Submitting Author accepts and understands that any supply made under these terms is made by BMJ to the Submitting Author unless you are acting as an employee on behalf of your employer or a postgraduate student of an affiliated institution which is paying any applicable article publishing charge ("APC") for Open Access articles. Where the Submitting Author wishes to make the Work available on an Open Access basis (and intends to pay the relevant APC), the terms of reuse of such Open Access shall be governed by a Creative Commons licence – details of these licences and which Creative Commons licence will apply to this Work are set out in our licence referred to above.

Other than as permitted in any relevant BMJ Author's Self Archiving Policies, I confirm this Work has not been accepted for publication elsewhere, is not being considered for publication elsewhere and does not duplicate material already published. I confirm all authors consent to publication of this Work and authorise the granting of this licence.

For peer review only

Factors influencing implementation and sustainability of interventions to improve oral health and related health behaviours in adults experiencing severe and multiple disadvantage: A mixed-methods systematic review

Authors
Mrs. Deepti A. John, MDPH - Population Health Sciences Institute, Newcastle University, Newcastle Upon Tyne NE2 4AX, UK
Ms. Emma A. Adams, MSc. - Population Health Sciences Institute, Newcastle University, Newcastle Upon Tyne NE2 4AX, UK
Dr. Laura J. McGowan, PhD - Population Health Sciences Institute, Newcastle University, Newcastle Upon Tyne NE2 4AX, UK
Dr. Emma C. Joyes, PhD - Population Health Sciences Institute, Newcastle University, Newcastle Upon Tyne NE2 4AX, UK
Ms. Catherine Richmond, MA - Population Health Sciences Institute, Newcastle University, Newcastle Upon Tyne NE2 4AX, UK
Dr. Fiona R. Beyer, PhD - Evidence Synthesis Group and Innovation Observatory, Population Health Sciences Institute, Newcastle University, Newcastle Upon Tyne NE2 4AX, UK
Mr. David Landes, BDS - NHS England & Improvement, Newcastle Upon Tyne NE15 8NY, UK
Prof. Richard Watt, PhD - Department of Epidemiology and Public Health, University College London, London WC1E 7HB, UK
Prof. Falko F. Sniehotta, PhD - NIHR Policy Research Unit Behavioural Science, Newcastle University, NE2 4AX And Department of Public Health, Social and Preventive Medicine, Centre for Preventive Medicine and Digital Health (CPD), Medical Faculty Mannheim, Heidelberg University, 68167 Mannheim
Dr. Martha Paisi, PhD - Faculty of Medicine and Dentistry, Peninsula Dental School, University of Plymouth, Plymouth PL4 8AA, UK
Prof. Clare Bambra, PhD - Population Health Sciences Institute, Newcastle University, Newcastle Upon Tyne NE2 4AX, UK
Prof. Dawn Craig, MSc. - Population Health Sciences Institute, Newcastle University, Newcastle Upon Tyne NE2 4AX, UK
Prof. Eileen Kaner, PhD - Population Health Sciences Institute, Newcastle University, Newcastle Upon Tyne NE2 4AX, UK
Prof. Sheena E. Ramsay, PhD - Population Health Sciences Institute, Newcastle University, Newcastle Upon Tyne NE2 4AX, UK

Corresponding Author:
Deepti John
Deepti.john@newcastle.ac.uk
Phone no: +44 7469646623
Population Health Sciences Institute, Newcastle University
The Baddiley-Clark Building, Richardson Road
Newcastle upon Tyne, NE2 4AX
United Kingdom

For peer review only

Abstract

Objectives

Among people experiencing severe and multiple disadvantage (SMD) poor oral health is common and linked to smoking, substance use and high sugar intake. Studies have explored interventions addressing oral health and related behaviours; however, factors related to the implementation of these interventions remains unclear. This mixed-methods systematic review aimed to synthesize evidence on the implementation and sustainability of interventions to improve oral health and related health behaviours among adults experiencing SMD.

Methods

Bibliographic databases (MEDLINE, EMBASE, PsycINFO, CINAHL, EBSCO, Scopus) and grey literature were searched from inception to February 2023. Studies meeting the inclusion criteria were screened and extracted independently by two researchers. Quality appraisal was undertaken, and results were synthesised using narrative and thematic analyses.

Results

Seventeen papers were included (published between 1995-2022). Studies were mostly of moderate quality and included views from SMD groups and service providers. From the qualitative synthesis, most findings were related to aspects such as trust, resources, and motivation levels of SMD groups and service providers. None of the studies reported on diet and none included repeated offending (one of the aspects of SMD). From the quantitative synthesis, no difference was observed in program attendance between the interventions and usual care, although there was some indication of sustained improvements in participation in the intervention group.

Conclusion

This review provides some evidence that trust, adequate resources, and motivation levels are potentially important in implementing interventions to improve oral health and substance use among SMD groups. Further research is needed from high quality studies and focusing on diet in this population.

Strengths and limitations of this study

- Comprehensive search strategy was used to gather evidence in this mixed methods systematic review.

For peer review only

- Consolidated Framework for Implementation Research (CFIR) was used for the data extraction.
- Confidence in the papers were limited due to moderate quality of the papers.
- The included studies were not excluded based on their quality, as they contributed relevant information for this systematic review.

Keywords: multiple disadvantage, homeless, oral health, implementation

INTRODUCTION

Severe and multiple disadvantaged (SMD) populations are individuals who have experienced homelessness, substance use, offending or a combination of all three.(1) They experience disproportionately high levels of poor physical and mental health along with high levels of occupational deprivation,(1) which results in isolation and difficulty in accessing healthcare services.(2) There is also an added burden of stigma that affects their access and engagement.(3)

Among people experiencing SMD, oral health problems have been highlighted as one of the major unmet needs.(4) Aggravated by high levels of smoking, substance and alcohol use and poor diet (high intake of sugar).(4, 5) Elevated tobacco use make them more susceptible to periodontal disease, tooth loss, oral lesions and oral cancer.(5, 6) Research also shows that they do not meet the daily nutritional requirements and have high levels of sugar consumption.(6, 7) Oral health has an overall impact on physical and mental wellbeing.(8) It is, therefore, important to not only address oral health concerns in people experiencing SMD, but also related health behaviours such as smoking, alcohol and substance use, and poor nutrition.(1, 9)

Previous papers focus on intervention design and outcomes, none focus on the implementation approach of these intervention especially in people experiencing SMD.(10, 11, 12) Hence, there is a need for evidence on interventions addressing these health challenges, with a specific focus on ways to improve implementation and long-term sustainability of interventions. Frameworks are used to apply a theoretical underpinning to our understanding of why implementation of interventions succeed or fail. The Consolidated Framework for implementation Research (CFIR) composed of five domains was utilised as a theoretical framework to identify the facilitators and barriers that influence implementation. (13, 14) This framework, therefore, assists with bridging the gap between research and practice as well as reducing the challenges of implementing these interventions.(15)

To investigate how we can improve implementation and sustainability, we conducted this systematic review to synthesize various factors such as acceptability, settings, and potential adverse effects of interventions that improve oral health and related health behaviours of adults with SMD.

For peer review only - http://bmjopen.bmj.com/site/about/guidelines.xhtml

METHODS

The research protocol was pre-registered and published registered with the Prospective Register of Systematic Reviews (PROSPERO) (reg. no: CRD42020202416).(16, 17) The review was reported according to Preferred Reporting Items for Systematic Reviews and Meta-Analyses (PRISMA) guidelines.(18)

Search strategy

The search strategy (see Supplementary file.) was formulated and conducted with an information specialist within the research team. The following electronic databases: MEDLINE (Ovid), EMBASE (Ovid), CINAHL (Ebsco), APA PsycINFO (Ovid) and Scopus were searched for relevant qualitative, quantitative, and mixed method studies from inception to February 2023. Grey literature searches were conducted using Google Incognito and selected charity organization websites such as Fulfilling Lives, Crisis, Groundswell, which were informed by the expertise of the research team. Forward and backward citation search of the included studies were also conducted.

Study selection

The search results were downloaded and deduplicated using EndNote 20.4.1 and the uploaded into Covidence, an online tool for managing the whole systematic review process.(19) Title, abstracts and full texts were independently screened by two reviewers. In the case of a discrepancy, consensus was reached after consultation with a third reviewer. Table 1 presents the inclusion criteria used during screening.

Table 1: *Eligibility Criteria used to select the studies*

	Eligibility Criteria
Population	Adults aged 18 or above, who experience SMD comprising of either homelessness (rough sleeping or other types of insecure accommodation), repeated offending or frequent substance use that co-occurs with homelessness or repeated offending.(17) Perspectives of staff who work with SMD groups and stakeholders such as policy makers and commissioners.
Intervention	Structural, community and individual level interventions.(17)
Outcomes	Views from SMD groups and other stakeholders (policy makers, service providers, voluntary sector etc) about implementation and sustainability of interventions which include acceptability, content, settings, potential harms, uptake, and retention.(17)
Study Design	Qualitative, quantitative, and mixed method studies

Data extraction and quality appraisal

The data extraction and quality assessment for all the included studies were conducted by one reviewer and cross-checked by a second reviewer. Included studies were critically appraised to guide

For peer review only - http://bmjopen.bmj.com/site/about/guidelines.xhtml

how much confidences could be placed on the findings. Qualitative studies were appraised using the Critical Appraisal Skills Programme (CASP) qualitative checklist.(20) Quantitative studies were appraised using Cochrane's Risk of bias for randomized control trials (RCTs).(21) For cross-sectional studies the National Institutes of Health (NIH) Study Quality Appraisal Tool was used.(22) Qualitative studies were rated as good, moderate or low quality, which was informed by a scoring system: scores 9–10 was high quality, 7.5–9 was moderate quality and <7.5 was low quality.(23) The scoring were informed by the quality checklists. Studies were not excluded based on their quality; poor reporting is not always reflective of poor methodology. (24) Studies were included on whether they contributed data relevant or novel data to this review.(24) Moreover, including all studies allowed gathering the global evidence related to the review questions.

Data synthesis

Abstracts and data from the results of included studies were uploaded on to NVivo software (QSR International Pty Ltd., Melbourne, Australia Version 12, Release 1.6.1). Narrative synthesis was undertaken. Deductive codes based on the CFIR framework were used to initially code the findings followed by a three-step inductive synthesis process which involved coding the text, identifying the themes, and creating the subthemes. To maximise thematic yield, data reported in different papers but from the same study were individually coded. The developing themes and subthemes were discussed with the other reviewers and consensus was reached regarding these.

Patient and public involvement

Patients and/or the public were not involved in the design, or conduct, or reporting, or dissemination plans of this research.

RESULTS

Seventeen articles (twelve individual studies) met the inclusion criteria and were included in this systematic review. Figure 1 presents the PRISMA flowchart for included studies. Table 2 presents the descriptive summaries of the included studies. The papers were published between 1995 and 2022, and were related to interventions targeting oral health,(25, 26, 27, 28, 29, 30, 31, 32) substance use,(33, 34, 35, 36, 37, 38, 39) smoking and none on diet.

For peer review only - http://bmjopen.bmj.com/site/about/guidelines.xhtml

Table 2: *Descriptive summaries of the seventeen included studies, including quality appraisal (High quality/moderate quality/low quality)*

No.	First Author (Year) and Country	Sample Size and Age of the participants	Participant Group	Intervention Description	Type of Research, Data Collection and Analysis	Findings of the paper relevant to the review	Quality Appraisal/ Risk of Bias
1.	Beaton et al (2016) United Kingdom(25)	N=20, age not mentioned	Health and social care workers	Motivational interviewing to promote oral health among homeless populations ("Smile4life programme")	Qualitative - Telephone interviews, framework approach	Familiarity and good relationships between service providers and third sector organizations facilitated implementation whereas lack of resources and interest hindered it	High quality
2.	Beaton et al (2018) United Kingdom (26)	N= 9 observation sessions, age not mentioned	Oral healthcare workers such as oral health educators and dental support workers	Motivational interviewing and tailored advice to promote oral health among the homeless population at different settings such as mobile dental units and homeless shelters ("Smile4Life programme")	Qualitative- Participant observation, content analysis	Good working relationships between healthcare providers, patients and third sector organizations are important	Moderate quality
3.	Beaton et al (2021) United Kingdom (27)	N= 100, 16- 85 years	Oral health practitioners, third sector organization staff and local authority staff	Motivational interviewing and behavioural change techniques to promote oral health among the homeless ("Smile4Life programme")	Quantitative- Questionnaire, K-R20, Exploratory factor analysis, multivariate path analysis.	Work practices such as positive attitudes and beliefs of the oral healthcare workers influence implementation	Moderate/ Fair quality
4.	Burnam et al (1995) United States of America(34)	N= 276, mean age = 37 years	Homeless individuals with co-occurring substance and mental health issues	Social model of residential and non-residential programs providing integrated substance use and mental health services	Quantitative- Structured interviews, regression analyses	Retention levels were higher in the residential program compared to the non-residential one	Low quality (high Risk of Bias)
5.	Coles et al (2013) United Kingdom(29)	N= 14, age not mentioned	Healthcare workers from statutory and non-statutory organizations	A framework that offers tailored oral health advice and signposts to relevant dental services. ("Something To Smile About")	Qualitative- Focus groups, content analysis	Oral health knowledge among the healthcare workers improved but complex needs such as housing, employment etc must be addressed prior to oral health for successful implementation	Moderate quality
6.	Collins et al (2019) United States of America(35)	N= 168, mean age= 47 years	Homeless individuals with alcohol use disorder	Non-abstinence treatment program that involves tracking of alcohol use, discussion of safe drinking practices and goal-oriented tasks. ("Harm Reduction Treatment for Alcohol HaRT-A")	Quantitative-Questionnaires, content analysis	It was positively viewed by the participants with high levels of retention and satisfaction	Good quality (low ROB)
7.	Doughty et al (2020) United Kingdom(31)	Service users – N= 353, age not mentioned Service providers – not stated	Homeless individuals and oral healthcare workers such as dentists, dental nurses, dental technicians etc	Denture service provided by Crisis at Christmas Dental Service and Den-tech to the homeless and vulnerably housed	Qualitative	Communication, timing, resources, and training were considered as areas that needed to be improved	Low quality
8.	Forchuk et al (2022) Canada(36)	Service users – N= 58, mean age = 52.5 years Service providers – not stated	Homeless veterans with substance use problems and staff from housing services	Housing provided along with the peer support and harm reduction services to homeless veterans ("Housing First")	Qualitative - Interviews and focus groups, Thematic analysis	Stable housing with harm reduction services was well received. Collaboration between mental health and addiction services should be considered for future services	Low quality
9.	Henderson et al (2004) United States of America(37)	Service users – N = 15 Service providers – not mentioned	Homeless veterans with substance/alcohol use and program staff such as	Residential substance use treatment program that focuses on relapse prevention along with education and housing stability for homeless men	Qualitative - Surveys, direct observation and Interviews, not stated	Majority of the participants provided positive feedback. Staffing issues such as training and	Moderate quality

For peer review only - http://bmjopen.bmj.com/site/about/guidelines.xhtml

No.	First Author (Year) and Country	Sample Size and Age of the participants	Participant Group	Intervention Description	Type of Research, Data Collection and Analysis	Findings of the paper relevant to the review	Quality Appraisal/ Risk of Bias
			healthcare workers and administrative staff			competing workload were noted as drawbacks to the program	
10.	Neale et al (2014) United Kingdom(33)	Service users - N= 30, 23- 62 years<br>Service providers – N= 15, age not mentioned	Homeless individuals with substance use and mentors such as substance use workers, substance use managers and hostel staff	Computer assisted therapies using 20 different psychosocial intervention strategies to identify and reduce substance use based in hostels and homeless shelters ("Breaking Free Online")	Qualitative- Interviews, Inductive coding and Framework approach	'Program features', 'mentor support', 'participant characteristics' and 'delivery context' were noted as factors that lead to successful delivery.	Moderate quality
11.	Paisi et al (2020) United Kingdom (32)	Service users – N= 11, 20 -65 years<br>Service providers – N= 11, age not mentioned	Homeless individuals and the dental clinic staff members, support workers and volunteers.	Community dental clinic that provides both regular and emergency treatments.	Qualitative – semi structured interviews, reflective thematic analysis	Flexibility and the relationship between the patient and dental provider were highlighted as important features.	High quality
12.	Pauly et al (2020) Canada(38)	N= 14, 29-61 years	Homeless with illicit alcohol use	Non-residential community managed alcohol program which provides harm reduction strategies and peer support ("Canadian Managed Alcohol Program Study")	Qualitative- Semi- structured interviews, inductive coding and constant comparative analysis	Peer led program was successful as it facilitates capacity building, engagement, and empowerment	Moderate quality
13.	Pratt et al (2019) United States of America(40)	N= 40, 29-69 years	Homeless with smoking and alcohol use	Nicotine replacement therapy and motivational interviewing/cognitive behavioural therapy to reduce smoking and alcohol use among the homeless ("Power To Quit 2")	Qualitative- Interviews, social constructivist approach to grounded theory	Social (peer groups) and environmental (housing etc) factors impact cessation in homeless smokers	High quality
14.	Pratt et al (2022) United States of America (41)	N= 40, 29-70 years	Homeless with smoking and alcohol use	Nicotine replacement therapy and motivational interviewing/cognitive behavioural therapy to reduce smoking and alcohol use among the homeless ("Power To Quit 2")	Qualitative – Semi structured interviews, social constructivist approach to grounded theory	Social pressure and shelter environment impact the intervention but the integrated treatment along with emotional support from the staff make it beneficial.	High quality
15.	Rash et al (2017) United States of America(39)	N= 355, mean age = 37 years	Homeless with substance use	Behavioural intervention contingency management with the use of incentives such as vouchers and prizes delivered at local community clinics	Quantitative-Adaptation of the Service Utilization Form, Multivariate analysis of variance.	Retention was higher in groups that accessed the intervention compared to the standard arm of care	Moderate/fair quality
16.	Rodriguez et al (2019) United Kingdom(28)	Service users - N= 13, 18- 22 years<br>Service providers – N= 5, age not mentioned	Young homeless people and NGO practitioners	Pedagogical workshops about oral health, mental health, substance misuse, diet etc to increase engagement and awareness	Qualitative- Unstructured interviews and workshops, content analysis	Involvement of young people in co-designing an intervention facilitates engagement, trust building and increases health literacy	High quality
17.	Stormon et al (2018) Australia(30)	N= 76, 41-60 years<br>Feedback – N=24	Disadvantaged adults (clients of community organizations that utilize housing, employment and food services)	Facilitated access pathway between homeless organizations and public dental services. Improving oral health by assessing dental needs, offering dental advice and dental appointments	Quantitative-Questionnaire, Descriptive analysis, and Framework approach	Positive feedback by participants facilitated by the environment, clinical staff and flexibility. Attendance rates varied across the site but was generally high.	Fair/ moderate quality

For peer review only - http://bmjopen.bmj.com/site/about/guidelines.xhtml

SMD groups in the studies found in this review included young adults, single mothers, veterans, and adults with co-occurring conditions of severe mental illness. Based on the information reported in the studies, most of the interventions were focused on adults who were experiencing homelessness and substance use issues,(33, 34, 35, 36, 37, 38, 39, 40) but did not explicitly report on whether they included those who had repeated involvement with the criminal justice system.

Quality appraisal

Of the twelve articles reporting qualitative findings, two were low quality due to lack of detailed findings and methodology not being reported adequately,(31, 36) five moderate quality,(26, 29, 33, 37, 38) due to reporting bias and five high quality.(25, 28, 32, 40, 41) The risk of bias was assessed for the five articles reporting quantitative findings; among the two RCTs: one had a high risk of bias because of attrition and reporting bias,(34) and the other article had a low risk of bias,(35) the remaining three cross-sectional studies were of moderate quality.(27, 30, 39) The findings reported in this review are mostly from high or moderate quality articles, with the inclusion of insights from low quality articles employed strategically for completeness of reporting evidence available and to supplement findings from the adequately reported articles.

Synthesis of qualitative findings

Table 3 presents the themes, subthemes, codes, and quotes from individuals experiencing SMD and frontline staff and stakeholders.

For peer review only - http://bmjopen.bmj.com/site/about/guidelines.xhtml

Table 3: *Themes and subthemes from qualitative synthesis of findings along with the relevant codes and quotes*

Themes	Subthemes	Codes	Quote(s) – people with SMD; or frontline staff
Intervention settings	Physical settings	Housing stability, privacy, confidentiality	*"This is kind of a stressful situation. People are homeless, being at the bottom of their luck, and—boom—and everything. So this is stress. What do you do? You drink, and you smoke, and that's all that you can do, walking around here all day. Do you understand?"(person with SMD) (40)*<br><br>*"But at the same time the addictions piece, especially in terms of stability, I've noticed a lot of the guys that because they are stable in our home, they may make the choice more often to say 'I don't feel like drinking tonight,' so they don't. They don't have to get intoxicated to go to sleep in a shelter on a mat, they can choose not to drink and sometimes they do make that choice not to drink and just watch TV for the evening."(frontline staff) (36)*<br><br>*"If you went in and tried to do anything, people were behind you, over your shoulder, 'what are you doing there' And, you know, I didn't what to discuss with people what I was doing, because they'd take the mick"(person with SMD) (33)*
	Psychological aspects of settings	Communication, trust building, familiarity, mentorship, community, peer pressure, guidance, support, safe space	*"It's not just about dental treatment, I think for a lot of people there is the fear of the dentist because when they do go, it's because they need work done and they're in pain, therefore they associate pain with the dentist."(frontline staff) (29)*<br><br>*"She's not somebody that normally expresses much in a group, she's quite a private person, so I thought it took quite a lot for her to open up, to trust, but I also appreciate the fact that she felt she was in a really good space that she could share that experience with the others and I felt that was really valuable for the rest of the group to hear that. I think this activity [the workshops] encourages people to talk about their own experiences"(frontline staff)(28)*<br><br>*"Yes. A lot better off because… I'm not like, like when I'm here and I'm here with people that are drinking on programs like this and stuff like that I've noticed we're all on the same level. We don't care about the issues or problems, we just, you know, pitch together and do what we gotta do to get ourselves fixed and then from there if we can help other people, and people help other people…"(person with SMD) (38)*<br><br>*"If there is nobody there and you're just left to get on with it, it's quite easy to skip things…I will just put that answer down, you know. But then when you know somebody is there and they are there for that specific reason, then it's a lot easier to go through with things."(person with SMD) (33)*
	Accessibility	Point of contact, space, geography	*"We were put in the medical room along the corridor from the office, but there was no opportunity for practitioner 4 to approach any of the residents. We only saw service users if they specifically wanted to talk about their oral health or if they had walker past the room and wanted to see who we were." (frontline staff) (26)*<br><br>*"I'm from a very rural area, and we don't really have any homelessness centres." (person with SMD) (25)*
Intervention delivery	Improved Awareness	Understandable, ideas, learning from one and another	*"Take things that people say and take it on board, and everything's a learning curve, you learn things all the time… And I'd recommend that to anybody else who is homeless, just listen to other people, take on board what they've got to say, and accept the help that's around you like the group activity [the workshops]".(person with SMD) (28)*<br><br>*"Especially when it was to do with what alcohol can do and what substances can do, I don't think they realized how that affects their oral health, their ears pricked up when you said that" (frontline staff) (29)*

For peer review only - http://bmjopen.bmj.com/site/about/guidelines.xhtml

Themes	Subthemes	Codes	Quote(s) – people with SMD; or frontline staff
	Resources	Workloads, stress, competing needs, volunteers, equipment, funds	*"I think he [client] felt that maybe I would have to sit with him again and, I don't know, maybe I should have sat him down and had a talk with him and I just haven't been able to"(frontline staff) (33)*<br><br>*"You feel like you're spinning so many plates, that you just can't possibly keep them all up in the air" (frontline staff) (25)*<br><br>*"we need to attract funding … it's very difficult to encourage NHS England to commission outside of their routine, the existing contract doesn't favour patients with high treatment needs so we would need them to step outside of their comfort zone and commission something slightly different to what they're used to"(frontline staff) (32)*
	Perceived risks while working with a vulnerable population	Safety, unpredictable, inappropriate behavior, challenges, relevant experience, confident, challenging behaviors	*"Practitioner 1 is confident and appears quite fearless, putting up with language/behaviour that would not be tolerated in a normal clinic." (researcher observation notes of frontline staff) (26)*<br><br>*"Initially we were thinking 'oh we need to make sure that we're not alone in the surgery at any point', and we had a panic alarm and things, we still have all that in place, but it's actually been fine."(frontline staff) (32)*
Ways to enhance engagement and participation	Interest and motivation	Complexity, fears, initiative, specific and complex needs, mixed opinions	*"[Mentor] came in and said 'I'm going home, have you done much?' And I said, 'I couldn't get back on, you know'. And she just took it [the laptop]. I don't know if she was fed up with me or whatever, but she never spoke about it again and I never mentioned it again."(person with SMD) (33)*<br><br>*"The oral health team do not seem bothered to recruit any patients, even if that means sitting waiting with nothing to do—the feeling seems to be that if a patient wants to be seen then they will come to the MDU."(researcher observation notes of frontline staff) (26)*<br><br>*"My goal is to quit within a month or two months. I talked to a couple of people. 'It ain't going to happen.' I said, 'well if you set your mind to certain things, you can do this."(person with SMD) (40)*<br><br>*"I think it's good. It made me feel like I had something to do or like I had a purpose. You know what I mean, not a purpose but it wasn't like the homeless"(41)*
	Adapt to specific circumstances	Context, tailored to the needs of the individual, personalized care	*"People getting through the door, they might not have a roof, might not have any money, might have major drug and alcohol issues, might be threatened with violence, the last thing they want to talk about is their teeth."(frontline staff) (29)*<br><br>*"'we call these people chaotic and that's a bit judgmental, they are actually setting priorities, they've got so much going on in their lives that it [oral health] just falls of their list of priorities, they're saying 'it's my priority to find somewhere to sleep tonight' … The time that you catch people' was therefore identified as 'really important'."(frontline staff)* (32)
	Constant support	Long term care, advice, support	*"About three or four in the morning and I feel like upset then…I can come down and use the program, which is quite good because that way I can put stuff that is all jumbled up in my head down in a way that makes sense and it kind of makes you see that things aren't quite so bad as they seem."(person with SMD) (33)*<br><br>*"At the stage of having goals, an action plan and were working through that . . . but for some homeless people who are nowhere ready, you can make an average of seven appointments before they will turn up once, it's just where your client is at."(frontline staff) (29)*

For peer review only - http://bmjopen.bmj.com/site/about/guidelines.xhtml

Synthesis of twelve papers with qualitative findings,(25, 26, 28, 29, 31, 32, 33, 36, 37, 38, 40, 41) identified three overarching themes in relation to the aims of this review. The three themes are: 1) Intervention settings, 2) Intervention delivery and 3) ways to enhance engagement and participation.

Theme 1: Intervention settings

Eleven papers identified issues related to the settings of interventions which can play a role in the delivery of interventions targeting oral health, substance use and smoking.(25, 26, 27, 28, 32, 33, 36, 37, 38, 40, 41)

Physical settings

Physical settings involved the environment in which the intervention took place. The wider physical environment has been found to have an impact on the intervention experience,(41) with privacy being the key factor for improving physical settings.(32, 33, 41) Communal homeless shelters and busy teaching hospitals lack the space and privacy to deliver interventions involving discussions about difficult and sensitive topics.(32, 33, 41) Contrastingly, stable housing with the necessary privacy allowed people experiencing SMD to focus on their recovery journey, whilst also creating a space in which residents could spend time away from peers who were sometimes perceived as having a negative peer group influence.(36, 40)

Psychological aspects of settings

Psychological aspects related to the less visible parts of the interventions were identified across ten papers.(25, 26, 28, 29, 32, 33, 37, 38, 40, 41) Firstly, it was reported that relationships between people experiencing SMD and service providers played a vital part in the delivery of interventions. Through good communication,(26, 28, 32, 41) trust building,(28, 29, 32, 41) familiarity of working with a vulnerable population,(25, 32) and mentorship,(33, 37) interventions were able to form a 'safe and respectable environment'.(28, 32, 37, 41) Secondly, papers discussed the importance of peer support as a way of increasing the effectiveness of interventions.(32, 37, 38) There were also reports of the impact negative peer influence could have on the recovery process. For example, smoking and drinking were linked to socializing with others, which could increase the urge to smoke or drink.(40, 41)

Accessibility

Accessibility of interventions was one of the factors found to be important related to implementation of interventions among people experiencing SMD.(25, 26, 32, 41) Firstly, accessible

For peer review only - http://bmjopen.bmj.com/site/about/guidelines.xhtml

and spacious meeting points within the services were reported to help with their participation in the intervention, especially in the case of oral health interventions that were delivered either in a community setup (open space) or a mobile dental van .(26) Secondly, geographical proximity could act as a barrier as rural and remote areas lack the facilities and resources, which could influence the access of people experiencing SMD.(25, 32) Lastly, it was reported that access could become an issue when service users move to more stable housing as weather conditions, distance, work and other appointments tend to make it challenging to attend the intervention sessions.(41)

Theme 2: Intervention delivery

Nine papers discussed aspects such as information availability, resources and perceived risks of working with a vulnerable population that could be important for roll out and delivery of interventions addressing oral health, smoking and substance use.(25, 26, 28, 29, 31, 32, 33, 37, 41)

Improved Awareness

Awareness and information availability were discussed in papers focusing on improving oral health, smoking and alcohol use.(28, 29, 32, 41) Sharing information between service providers and SMD groups was identified as an important issue across the papers as it created opportunities to promote involvement and behavior change.(28, 29, 41) It was reported that easily understandable information encouraged people experiencing SMD to view healthier behaviors as important (e.g. tooth brushing) and helped to signpost them to necessary services.(28, 41) Clear and simple explanations of treatment options available was seen to help them in decision-making.(32) Service providers also felt that they learned more about healthy behaviors and were able to pass their newly gained knowledge to their clients.(29)

Resources

Five papers discussed the importance of having necessary resources to enable interventions to run efficiently and effectively.(25, 31, 32, 33, 37) The majority of these highlighted the importance of distribution of workloads among staff because of difficulties in implementing interventions with competing duties and work within the organizations.(25, 33, 37) Funding and resources such as volunteers and materials were identified in oral health interventions as an important issue that impacts implementation and long-term sustainability.(31, 32)

Perceived risks working with a vulnerable population

Papers reported on the perceived risks of delivering interventions to vulnerable populations as challenging at times by service providers.(25, 26, 32) There were concerns about safety of service

For peer review only - http://bmjopen.bmj.com/site/about/guidelines.xhtml

providers while interacting with clients who were seen to be “unpredictable”. The need for training and being better equipped to work in this environment and setting boundaries between service providers and clients was repeatedly mentioned by service providers.(25, 26, 32, 37) The papers also highlighted the importance of training opportunities that provide service providers with the necessary skills to handle volatile and difficult situations.(25, 37)

Theme 3: Ways to enhance participation and engagement

Ten papers identified factors such as interest and motivation levels, adaptability, and long-term support that could help to improve outcomes and create sustainable interventions by enhancing engagement and participation.(25, 26, 28, 29, 31, 32, 33, 37, 40, 41)

Interest and motivation

Nine papers highlighted that the interest and motivation levels of both staff supporting SMD groups and people experiencing SMD play an important role in the implementation of interventions. Disinterest was sometimes observed amongst service providers, due to concerns about the complexity of delivering the intervention,(25, 29, 31) lack of engagement with third sector organizations,(26) poor uptake of the intervention by the target populations,(25, 29, 31) and preconceived notions of improper behaviour by SMD groups.(41) Interestingly, interventions were met with similar feelings of indifference by people experiencing SMD if the intervention did not address their specific and complex needs such as housing and financial problems.(25, 26, 32) Two papers on oral health interventions found that younger adults and families with children were more eager to engage compared to single men.(28, 29) Papers discussing the same smoking intervention illustrated that an awareness of health benefits and risks played a part in motivating people in engaging with the intervention.(40, 41)

Adapting to specific circumstances

Adaptability of interventions was noted as an essential feature among four papers.(29, 32, 33, 40) Tailoring the interventions to address their specific needs at the time such as housing and employment was noted to increase participation and better outcomes.(29, 40) Service users of a community dental service also suggested flexible and longer dental appointments would be helpful and in the long-term these adaptions would help reduce missed appointments.(32) Another paper reported that people experiencing SMD were keen to have more face to face interactions rather than digital, which highlights the drive to more personalized care.(33)

Long term support

For peer review only - http://bmjopen.bmj.com/site/about/guidelines.xhtml

Four papers identified sustained and long-term support as a factor that could contribute towards better intervention outcomes.(29, 32, 33, 37) Service providers expressed a need for interventions which allowed people experiencing SMD to continue with services/programmes despite missing appointments or not completing treatment within the required delivery timeframe especially because of the transitionary nature of SMD groups.(32, 33) Similarly, for a substance reduction intervention, a preference for a long-term intervention, that allowed and supported them to gradually integrate into their new stage of their lives.(33, 37) Two papers on oral health interventions suggested that drop-in services offered flexibility in seeking advice or seeing a practitioner and helped to reduce anxiety surrounding accessing treatment for dental health.(29, 32)

Synthesis of quantitative findings related to retention and implementation

Four papers reported quantitative findings on retention and program attendance,(30, 34, 35, 39) as indicators of uptake and sustained implementation of interventions.

Three papers on substance use interventions reported high levels of retention in their intervention groups.(34, 35, 39) Two studies among them delivered the interventions along with housing services but the findings were mixed and limited on whether retention was significantly associated with the housing services or not. (34, 35, 39)

There was no difference in the attendance levels in the studies related to substance use interventions.(34, 35) The attendance level for an oral health promotion intervention delivered in community settings was high (85%), however it varied across community centers and was dependent on timing of appointment and dental treatments offered. More non-attendance seen for afternoon appointments and complex dental treatments (e.g. surgical and prosthodontic treatments).(30)

Additionally, workplace beliefs and practices amongst service providers such as knowledge, intention and goals, were reported to influence implementation behaviors.(27)

DISCUSSION

This review synthesized different factors that could influence the implementation and sustainability of interventions related to improving oral health and related health behaviors of people experiencing SMD. Evidence suggested that psychological aspects of intervention settings such as building trust and communication form an integral part in the creating a safe environment and that these are just as essential as the structural components of settings such as physical environment. Review findings further suggest that adequate staff capacity, funding and equipment would ease the delivery of interventions by reducing the immense pressure faced by service providers supporting

For peer review only - http://bmjopen.bmj.com/site/about/guidelines.xhtml

For peer review only

the interventions. It was also suggested that implementation is dependent on the interest and motivation of not only people experiencing SMD but also on that of service providers in delivering difficult and complex interventions.

Most of the included studies were related to oral health and substance use (drug and alcohol). There was a lack of evidence on diet and smoking interventions among this population. Previous evidence has shown that tobacco use and poor diet, often due to limited choice available while experiencing homelessness and related disadvantages, result in a range of adverse short term (nutritional deficiencies) and long term health outcomes (cancer, diabetes, heart disease).(42, 43, 44) Food insecurity is often linked to elevated tobacco use, mental health issues and an increased risk of substance misuse.(45, 46, 47)

While most of the papers mainly focused on the perspectives of people experiencing SMD, the limited data from service providers brought to light some of the challenges faced during implementation. This supports the notion that intervention implementation needs the co-ordination and collective effort of everyone involved. All the interventions included were designed focussing on service provision,(25, 26, 27, 28, 30, 31, 32, 33, 34, 35, 37, 38, 39, 40, 41, 48) except for one study which focussed on a training intervention for service providers (29).Limited evidence was available on the long-term sustainability of interventions, which highlights another evidence gap that needs to be addressed.

Our review findings suggest that the retention in interventions may depend on the type of treatment offered, which at times can be influenced by the availability of housing provision. Timing and type of treatment may also influence attendance rates; for instance, morning appointments might be more beneficial, especially for individuals struggling with alcohol addiction, as they may be less intoxicated compared to later in the day. Our review findings also complement our systematic review about the effectiveness of interventions that improve oral health and related health behaviors in SMD groups – the effectiveness review found that interventions that integrated health with the individual's wider needs (for e.g. housing, employment, mental health) were more effective than usual care.(49) The findings we have are very limited regarding retention and attendance, more effort needs to be taken to understand how to improve reach and retention among SMD groups so that they can access and use the interventions efficiently.

A systematic review on access to dental care among individuals experiencing homelessness in the UK identified similar findings around awareness, accessibility and organizational issues (lack of financial resources and collaboration between sectors) having an influence on implementation.(50) This was also similarly identified in another review on smoking cessation among homeless populations in

For peer review only - http://bmjopen.bmj.com/site/about/guidelines.xhtml

high-income countries.(51) The importance of continued engagement in services was highlighted in a review on substance use support for young people (ages 12 to 24) experiencing homelessness, which was also reflected in our findings.(52) Existing literature on interventions targeting health conditions such as HIV and Hepatitis C in this population have shown that improved health outcomes are linked to increased awareness, establishment of positive relationships with service providers and integrated treatment involving other health behaviours.(53, 54, 55)

Some findings from our review on aspects related to intervention settings, and intervention delivery aligned with CFIR constructs of Inner and Outer settings domains.(14) Subthemes in our findings on ways to enhance engagement, aligns with both Individuals and Implementation process domains.(14) The use of CFIR framework helps us understand the impact of intervention settings, delivery methods and engagement on the implementation process. It also provides a comprehensive approach for guiding the development of interventions targeting SMD groups and improving their efficacy in practical settings.

Strengths and Limitations

This systematic review is novel in that it assesses the implementation and sustainability of interventions on oral health together with co-occurring and related health behaviors in people experiencing SMD. It addresses an evidence gap on interventions targeting these health challenges and identifies ways to overcome implementation issues faced by these specific interventions. Another strength of this review lies in its comprehensive search strategy and use of a published tool (i.e. CFIR) to make sense of the results. It also highlights gaps in the evidence base on interventions related to diet, as well as studies that include repeat offenders. However, the confidence in the evidence from this review is limited as most of the papers were of moderate quality. Studies lacked detailed data collection methods and standardized evaluations which influenced their quality. Another limitation of this work is that intersectionality was not considered explicitly during the analysis of the data. Furthermore, the findings may not be generalizable to all contexts since the included papers were from high-income countries.

Implications

These findings offer valuable insights for enhancing existing interventions by paying attention to settings, delivery, and engagement opportunities. Evidence from this review points to the need for additional research on interventions targeting smoking and diet. These areas hold significant value due to their direct links with general and oral health. It is also important for interventions to address not only individual behaviours but also overlapping behaviours of substance use, smoking and poor

For peer review only - http://bmjopen.bmj.com/site/about/guidelines.xhtml

diet. This could help reduce the strain on resources and improve engagement. Furthermore, higher quality research that focuses more on sustainability and intersectionality is warranted to further investigate and refine interventions focused on SMD groups.

Author Contributions

Conceptualization: SER, EK, EAA, CB, RGW, ECJ, LJM, FB, DC, DL, MP, FFS; methodology: SER, DAJ, LJM, EAA, ECJ, CR, EK, DC, FB; draft preparation: SER, DAJ, EAA; writing - review and editing all; funding acquisition: SER, EK, FB, CB, RGW, DC, DL, MP, FFS.

Ethical approval

None sought.

Data Availability Statement

No data are available.

Funding Statement

This project is funded by the National Institute for Health and Care Research (NIHR) Policy Research Programme (NIHR200415). EAA was supported by the National Institute for Health and Care Research (NIHR) School for Public Health Research (SPHR) Pre-doctoral Fellowship, Grant Reference Number PD-SPH-2015. EK is supported by an NIHR Senior Investigator award and directs the NIHR funded Applied Research Collaboration North East and North Cumbria.

Conflicts of Interest

The authors declare no conflict of interest. The funders had no role in the design of the study; in the collection, analyses, or interpretation of data; in the writing of the manuscript, or in the decision to publish the results.

Word Count

3329 words

Figure 1: *PRISMA Flowchart for the search results.*

References:

For peer review only - http://bmjopen.bmj.com/site/about/guidelines.xhtml

1. Bramley G, Fitzpatrick S, Edwards J, et al. Hard edges: mapping severe and multiple disadvantage in England. 2015.
2. Sandu RD. Defining severe and multiple disadvantage from the inside: Perspectives of young people and of their support workers. Journal of Community Psychology. 2021;49(5):1470-86.
3. Rae BE, Rees S. The perceptions of homeless people regarding their healthcare needs and experiences of receiving health care. Journal of Advanced Nursing. 2015;71(9):2096-107.
4. Conte M, Broder HL, Jenkins G, et al. Oral Health, Related Behaviors and Oral Health Impacts Among Homeless Adults. J Public Health Dent. 2006;66(4):276-8.
5. Hertzberg D, Boobis S. Unhealthy State of Homelessness 2022: Findings from the Homeless Health Needs Audit. 2022.
6. Groundswell. Healthy Mouths: A peer-led health audit on the oral health of people experiencing homelessness. London Groundswell; 2017.
7. Evans NS, Dowler EA. Food, health and eating among single homeless and marginalized people in London. Journal of Human Nutrition and Dietetics. 1999;12(3):179-99.
8. Sheiham A. Oral health, general health and quality of life. SciELO Public Health; 2005. p. 644-.
9. Wiecha JL, Dwyer JT, Dunn-Strohecker M. Nutrition and health services needs among the homeless. Public Health Rep. 1991;106(4):364-74.
10. Beaton L, Humphris G, Rodriguez A,et al. Community-based oral health interventions for people experiencing homelessness: a scoping review. Community Dent Health. 2020;37(2):150-60.
11. Hwang SW, Tolomiczenko G, Kouyoumdjian FG, et al. Interventions to improve the health of the homeless: a systematic review. American journal of preventive medicine. 2005;29(4):311-. e75.
12. Speirs V, Johnson M, Jirojwong S. A systematic review of interventions for homeless women. Journal of Clinical Nursing. 2013;22(7-8):1080-93.
13. Damschroder LJ, Aron DC, Keith RE, et al. Fostering implementation of health services research findings into practice: a consolidated framework for advancing implementation science. Implementation Science. 2009;4(1):50.
14. Damschroder LJ, Reardon CM, Widerquist MAO,et al . The updated Consolidated Framework for Implementation Research based on user feedback. Implementation Science. 2022;17(1):75.
15. Shelton RC, Cooper BR, Stirman SW. The Sustainability of Evidence-Based Interventions and Practices in Public Health and Health Care. Annual Review of Public Health. 2018;39(1):55-76.
16. McGowan L, Joyes E, Adams E, et al. Systematic review of oral health and related health behaviour interventions in adults with severe and multiple disadvantages. PROSPERO. 2020.
17. McGowan LJ, Joyes EC, Adams EA, et al. Investigating the Effectiveness and Acceptability of Oral Health and Related Health Behaviour Interventions in Adults with Severe and Multiple Disadvantage: Protocol for a Mixed-Methods Systematic Review. Int J Environ Res Public Health. 2021;18(21).
18. Page MJ, Moher D, Bossuyt PM, et al. PRISMA 2020 explanation and elaboration: updated guidance and exemplars for reporting systematic reviews. BMJ. 2021;372:n160.

For peer review only - http://bmjopen.bmj.com/site/about/guidelines.xhtml

19. Covidence. Covidence Systematic Review Software.
20. Programme. CAS. CASP Qualitative Checklist 2022 [Available from: https://casp-uk.net/casp-tools-checklists.
21. Higgins JP, Thomas J, Chandler J, et al. Cochrane handbook for systematic reviews of interventions: John Wiley & Sons; 2019.
22. National Heart LaBI. Study quality assessment tool for observational and cross sectional studies 2021 [Available from: https://www.nhlbi.nih.gov/health-topics/study-quality-assessment-tools.
23. Butler A, Hall H, Copnell B. A Guide to Writing a Qualitative Systematic Review Protocol to Enhance Evidence-Based Practice in Nursing and Health Care. Worldviews Evid Based Nurs. 2016;13(3):241-9.
24. Saini M, Shlonsky A. Systematic synthesis of qualitative research: OUP USA; 2012.
25. Beaton L, Freeman R. Oral health promotion and homelessness: A theory-based approach to understanding processes of implementation and adoption. Health Education Journal. 2016;75(2):184-97.
26. Beaton L, Anderson I, Humphris G, et al. Implementing an Oral Health Intervention for People Experiencing Homelessness in Scotland: A Participant Observation Study. Dent J (Basel). 2018;6(4).
27. Beaton L, Humphris G, Rodriguez A, et al. Implementing the Smile4life intervention for people experiencing homelessness: a path analytical evaluation. BMC Oral Health. 2021;21(1):383.
28. Rodriguez A, Beaton L, Freeman R. Strengthening Social Interactions and Constructing New Oral Health and Health Knowledge: The Co-design, Implementation and Evaluation of A Pedagogical Workshop Program with and for Homeless Young People. Dent J (Basel). 2019;7(1).
29. Coles E, Watt C, Freeman R. 'Something to Smile About': An evaluation of a capacity-building oral health intervention for staff working with homeless people. Health Education Journal. 2012;72(2):146-55.
30. Stormon N, Pradhan A, McAuliffe A, et al. Does a facilitated pathway improve access to dental services for homeless and disadvantaged adults? Evaluation and program planning. 2018;71:46-50.
31. Doughty J, Johnson A, Tatari A, et al. A pilot denture service collaboration between Den-Tech denture charity and Crisis at Christmas Dental Service (CCDS): A Plan-Do-Study-Act analysis. British Dental Journal. 2020;228(3):183-90.
32. Paisi M, Baines R, Worle C, et al . Evaluation of a community dental clinic providing care to people experiencing homelessness: A mixed methods approach. Health Expectations. 2020;23(5):1289-99.
33. Neale J, Stevenson C. Positive and negative features of a computer assisted drug treatment program delivered by mentors to homeless drug users living in hostels. Journal of substance abuse treatment. 2014;47(4):258-64.
34. Burnam MA, Morton SC, McGlynn EA, et al. An experimental evaluation of residential and nonresidential treatment for dually diagnosed homeless adults. J Addict Dis. 1995;14(4):111-34.
35. Collins SE, Clifasefi SL, Nelson LA, et al. Randomized controlled trial of harm reduction treatment for alcohol (HaRT-A) for people experiencing homelessness and alcohol use disorder. Int J Drug Policy. 2019;67:24-33.

For peer review only - http://bmjopen.bmj.com/site/about/guidelines.xhtml

36. Forchuk C, Richardson J, Atyeo H, et al. Qualitative findings from a Housing First evaluation project for homeless Veterans in Canada. Journal of Military, Veteran and Family Health. 2022;8(2):27-38.
37. Henderson PM. Program evaluation of a long-term substance abuse treatment program for homeless men: Spalding University; 2003.
38. Pauly B, King V, Smith A, et al. Breaking the cycle of survival drinking: insights from a non-residential, peer-initiated and peer-run managed alcohol program. Drugs: Education, Prevention and Policy. 2021;28(2):172-80.
39. Rash CJ, Alessi SM, Petry NM. Substance abuse treatment patients in housing programs respond to contingency management interventions. J Subst Abuse Treat. 2017;72:97-102.
40. Pratt R, Pernat C, Kerandi L, et al. "It'sa hard thing to manage when you're homeless": the impact of the social environment on smoking cessation for smokers experiencing homelessness. BMC Public Health. 2019;19(1):1-8.
41. Pratt R, Xiong S, Kmiecik A, et al. The implementation of a smoking cessation and alcohol abstinence intervention for people experiencing homelessness. BMC public health. 2022;22(1):1260.
42. Baggett TP, Singer DE, Rao SR, et al . Food Insufficiency and Health Services Utilization in a National Sample of Homeless Adults. Journal of General Internal Medicine. 2011;26(6):627-34.
43. Fallaize R, Seale JV, Mortin C, et al. Dietary intake, nutritional status and mental wellbeing of homeless adults in Reading, UK. British Journal of Nutrition. 2017;118(9):707-14.
44. Okuyemi KS, Caldwell AR, Thomas JL, et al. Homelessness and Smoking Cessation: Insights from Focus Groups. Nicotine Tob Res. 2006;8(2):287-96.
45. Langnäse K, Müller MJ. Nutrition and health in an adult urban homeless population in Germany. Public Health Nutrition. 2001;4(3):805-11.
46. Knudsen HK. Implementation of smoking cessation treatment in substance use disorder treatment settings: a review. The American Journal of Drug and Alcohol Abuse. 2017;43(2):215-25.
47. Kim JE, Flentje A, Tsoh JY, et al . Cigarette Smoking among Women Who Are Homeless or Unstably Housed: Examining the Role of Food Insecurity. J Urban Health. 2017;94(4):514-24.
48. Beaton L, Anderson I, Humphris G, et al. Implementing an Oral Health Intervention for People Experiencing Homelessness in Scotland: A Participant Observation Study. Dentistry Journal. 2018;6(4):68.
49. McGowan LJ, John DA, Kenny RPW, et al . Interventions to improve oral health and related health behaviours of substance use, smoking and diet in people with severe and multiple disadvantage: a systematic review of effectiveness and cost-effectiveness. 2023.
50. Paisi M, Kay E, Plessas A, et al. Barriers and enablers to accessing dental services for people experiencing homelessness: A systematic review. Community dentistry and oral epidemiology. 2019;47(2):103-11.
51. Soar K, Dawkins L, Robson D, et al. Smoking amongst adults experiencing homelessness: a systematic review of prevalence rates, interventions and the barriers and facilitators to quitting and staying quit. Journal of Smoking Cessation. 2020;15(2):94-108.
52. Xiang X. A review of interventions for substance use among homeless youth. Research on Social Work Practice. 2013;23(1):34-45.

For peer review only - http://bmjopen.bmj.com/site/about/guidelines.xhtml

53. Nyamathi A, Kennedy B, Branson C, et al. Impact of Nursing Intervention on Improving HIV, Hepatitis Knowledge and Mental Health Among Homeless Young Adults. Community Mental Health Journal. 2013;49(2):178-84.
54. Young SD, Rice E. Online social networking technologies, HIV knowledge, and sexual risk and testing behaviors among homeless youth. AIDS and Behavior. 2011;15:253-60.
55. Slesnick N, Kang MJ. The impact of an integrated treatment on HIV risk behavior among homeless youth: a randomized controlled trial. Journal of behavioral medicine. 2008;31:45-59.

For peer review only

For peer review only - http://bmjopen.bmj.com/site/about/guidelines.xhtml

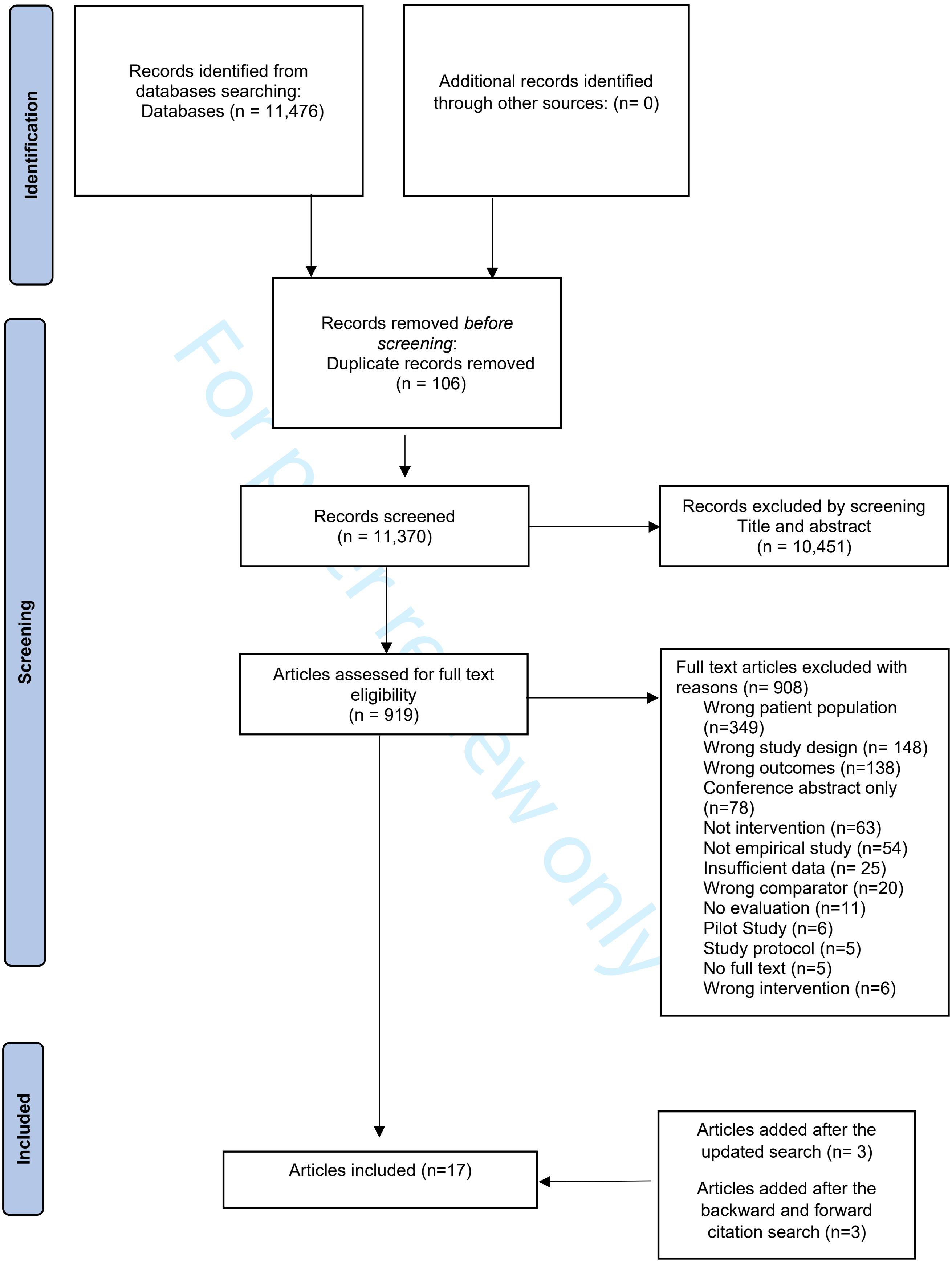

For peer review only

SMD Project – Search updates February 2023

Database(s): **Ovid MEDLINE(R) and Epub Ahead of Print, In-Process, In-Data-Review & Other Non-Indexed Citations, Daily and Versions** 1946 to January 30th 2023 **(Search run 3rd February 2023)**

Search Strategy:

#	Searches	Results
1	Oral Hygiene/	13719
2	Mouth Rehabilitation/	1470
3	Oral Health/	19795
4	exp Dental Health Services/	39658
5	((dental or oral or tooth or teeth or mouth) adj3 (health or care or hygiene or rehabilitation)).ti,ab,kw.	71845
6	or/1-5	105093
7	Smoking/	147692
8	(smoking or cigarette* or tobacco).ti,ab,kw.	346981
9	exp "Tobacco Use"/	9080
10	Alcohol Drinking/	74302
11	Alcoholism/	79526
12	Alcoholics/	871
13	(alcoholic* adj3 (person or people or adult or parent* or family)).ti,ab,kw.	947
14	"street drink*".ti,ab,kw.	8
15	exp Substance-Related Disorders/	306459
16	exp Drug users/	3922
17	Behavior, Addictive/	12185
18	((alcohol or drug* or substance*) adj2 (misuse* or abuse* or use* or addict* or dependenc* or issue* or problem*)).ti,ab,kw.	314183
19	(drug adj1 (habit or tak* or hard or illicit or inject*)).ti,ab,kw.	25327
20	or/7-19	873304
21	((sugar or sucrose or fructose or glucose) adj2 (intake or consum*)).ti,ab,kw.	16517

For peer review only

22	diet*.ti,ab,kw.	665093
23	"sugary food*".ti,ab,kw.	328
24	exp Dietary Sugars/ or Diet/	186792
25	((processed or acidic) adj1 food*).ti,ab,kw.	5331
26	((sugary or fizzy or carbonated or soft) adj1 drink*).ti,ab,kw.	5449
27	carbonated beverages/ or sugar-sweetened beverages/	3952
28	soda.ti,ab,kw.	4878
29	or/21-28	738771
30	(severe and multiple disadvantage*).ti,ab,kw.	11
31	Homeless Persons/	9442
32	homeless*.ti,ab,kw.	13348
33	((hous* or home* or accommodat* or shelter) adj3 (insecur* or instability or unstable or stability)).ti,ab,kw.	4473
34	or/30-33	18951
35	(probationer* or parolee* or ((repeat* or ex or re or revolving door or habitual or multiple or former* or previously*) adj1 (offen* or convict* or prisoner* or imprison* or incarcerat* or criminal*)) or (former adj3 inmate*) or ((community or probation* or parole* or reintegrat*) adj4 (prison* or offender* or criminal* or convict* or inmate*)) or ((individuals or men or women) adj2 (probation or parole)) or ((reintegrate* or reent* or return*) adj3 community)).ti,ab,kw.	4639
36	"criminal justice".ti,ab,kw.	5400
37	or/35-36	9464
38	((program* or policy or policies or strateg* or scheme* or project* or initiative* or "care package" or training or educat* or pilot or guidance or guideline* or study or pathway or treatment* or promot* or management or "support group" or process* or trial* or intervention*) adj5 (evaluat* or effect* or measur* or assess* or experiment* or impact* or feasab* or acceptab* or efficacy or perception* or belief* or uptake or consequence* or attitud* or barrier* or facilit* or motivat* or experience* or implement* or adher* or retention or retain* or reduc* or increas* or improv* or outcome* or cost* or benefit* or interview* or qualitative or ethnograph* or "focus group*")).ti,ab,kw.	5409160
39	"housing first".ti,ab,kw.	388
40	(outcome* adj5 evaluat*).ti,ab,kw.	106574

For peer review only

41	or/38-40	5450432
42	(34 or 37) and (6 or 20 or 29)	10263
43	41 and 42	5069

Database(s): **Embase** 1974 to 2023 January 30 **(Search run 3rd January 2023)**

Search Strategy:

#	Searches	Results
1	mouth hygiene/	30848
2	full mouth rehabilitation/	367
3	dental health/	4854
4	dental procedure/	30979
5	dental practice/ or dental prevention/ or exp dental restoration/	69380
6	((dental or oral or tooth or teeth or mouth) adj3 (health or care or hygiene or rehabilitation)).ti,ab,kw.	74077
7	or/1-6	169561
8	smoking/	360231
9	(smoking or cigarette* or tobacco).ti,ab,kw.	482602
10	"tobacco use"/	16485
11	drinking behavior/	54555
12	alcoholism/	128949
13	(alcoholic* adj3 (person or people or adult or parent* or family)).ti,ab,kw.	1320
14	"street drink*".ti,ab,kw.	15
15	exp drug dependence/	266514
16	addiction/	50440
17	((alcohol or drug* or substance*) adj2 (misuse* or abuse* or use* or addict* or dependenc* or issue* or problem*)).ti,ab,kw.	438421
18	(drug adj1 (habit or tak* or hard or illicit or inject*)).ti,ab,kw.	33544
19	or/8-18	1177022

For peer review only

20	((sugar or sucrose or fructose or glucose) adj2 (intake or consum*)).ti,ab,kw.	21137
21	diet*.ti,ab,kw.	830039
22	"sugary food*".ti,ab,kw.	432
23	sugar intake/	9669
24	diet/	244115
25	((processed or acidic) adj1 food*).ti,ab,kw.	6402
26	((sugary or fizzy or carbonated or soft) adj1 drink*).ti,ab,kw.	7130
27	carbonated beverage/	3672
28	sugar-sweetened beverage/	2782
29	or/20-28	902120
30	(severe and multiple disadvantage*).ti,ab,kw.	14
31	exp homeless person/	3638
32	homeless*.ti,ab,kw.	16533
33	((hous* or home* or accommodat* or shelter) adj3 (insecur* or instability or unstable or stability)).ti,ab,kw.	5665
34	or/30-33	21932
35	(probationer* or parolee* or ((repeat* or ex or re or revolving door or habitual or multiple or former* or previously*) adj1 (offen* or convict* or prisoner* or imprison* or incarcerat* or criminal*)) or (former adj3 inmate*) or ((community or probation* or parole* or reintegrat*) adj4 (prison* or offender* or criminal* or convict* or inmate*)) or ((individuals or men or women) adj2 (probation or parole)) or ((reintegrate* or reent* or return*) adj3 community)).ti,ab,kw.	5563
36	"criminal justice".ti,ab,kw.	6462
37	or/35-36	11379
38	((program* or policy or policies or strateg* or scheme* or project* or initiative* or "care package" or training or educat* or pilot or guidance or guideline* or study or pathway or treatment* or promot* or management or "support group" or process* or trial* or intervention*) adj5 (evaluat* or effect* or measur* or assess* or experiment* or impact* or feasab* or acceptab* or efficacy or perception* or belief* or uptake or consequence* or attitud* or barrier* or facilit* or motivat* or experience* or implement* or adher* or retention or retain* or reduc* or increas* or improv* or outcome* or cost* or benefit* or interview* or qualitative or ethnograph* or "focus group*")).ti,ab,kw.	7390414

For peer review only

39	"housing first".ti,ab,kw.	444
40	(outcome* adj5 evaluat*).ti,ab,kw.	168129
41	or/38-40	7453916
42	(34 or 37) and (7 or 19 or 29)	12372
43	41 and 42	6384

Database(s): **APA PsycInfo** 1806 to January Week 4 2023 **(Search run 3rd February 2023)**

Search Strategy:

#	Searches	Results
1	exp oral health/	1890
2	exp dental health/	594
3	((dental or oral or tooth or teeth or mouth) adj3 (health or care or hygiene or rehabilitation)).ti,ab.	3199
4	or/1-3	3613
5	tobacco smoking/	35146
6	(smoking or cigarette* or tobacco).ti,ab.	69114
7	alcohol drinking patterns/	26122
8	Alcoholism/	30862
9	alcohol abuse/	19136
10	(alcoholic* adj3 (person or people or adult or parent* or family)).ti,ab.	1753
11	"street drink*".ti,ab.	19
12	"substance use disorder"/	10378
13	drug abuse/	49214
14	drug addiction/	11973
15	addiction/	12827
16	((alcohol or drug* or substance*) adj2 (misuse* or abuse* or use* or addict* or dependenc* or issue* or problem*)).ti,ab.	183254
17	(drug adj1 (habit or tak* or hard or illicit or inject*)).ti,ab.	12930

For peer review only

18	or/5-17	279147
19	((sugar or sucrose or fructose or glucose) adj2 (intake or consum*)).ti,ab.	2160
20	diet*.ti,ab.	47688
21	"sugary food*".ti,ab.	62
22	Sugars/	2537
23	diets/	14955
24	((processed or acidic) adj1 food*).ti,ab.	396
25	((sugary or fizzy or carbonated or soft) adj1 drink*).ti,ab.	961
26	soda.ti,ab.	513
27	or/19-26	53562
28	(severe and multiple disadvantage*).ti,ab.	10
29	Homeless/	8126
30	homeless*.ti,ab.	12396
31	((hous* or home* or accommodat* or shelter) adj3 (insecur* or instability or unstable or stability)).ti,ab.	2080
32	or/28-31	14528
33	(probationer* or parolee* or ((repeat* or ex or re or revolving door or habitual or multiple or former* or previously*) adj1 (offen* or convict* or prisoner* or imprison* or incarcerat* or criminal*)) or (former adj3 inmate*) or ((community or probation* or parole* or reintegrat*) adj4 (prison* or offender* or criminal* or convict* or inmate*)) or ((individuals or men or women) adj2 (probation or parole)) or ((reintegrate* or reent* or return*) adj3 community)).ti,ab.	9014
34	"criminal justice".ti,ab.	13327
35	or/33-34	21049
36	((program* or policy or policies or strateg* or scheme* or project* or initiative* or "care package" or training or educat* or pilot or guidance or guideline* or study or pathway or treatment* or promot* or management or "support group" or process* or trial* or intervention*) adj5 (evaluat* or effect* or measur* or assess* or experiment* or impact* or feasab* or acceptab* or efficacy or perception* or belief* or uptake or consequence* or attitud* or barrier* or facilit* or motivat* or experience* or implement* or adher* or retention or retain* or reduc* or increas* or improv* or outcome* or cost* or benefit* or interview* or qualitative or ethnograph* or "focus group*")).ti,ab.	1427116

For peer review only

37	"housing first".ti,ab.	354
38	(outcome* adj5 evaluat*).ti,ab.	15300
39	or/36-38	1431044
40	(32 or 35) and (4 or 18 or 27)	8717
41	39 and 40	4427

CINAHL (via Ebsco) **Friday, February 3, 2023 12:16:53 PM**

#	Query	Results
S44	S41 AND S42 AND S43	3,512
S43	S5 or S18 or S29	551,298
S42	S34 or S37	20,783
S41	S38 OR S39 OR S40	1,823,817
S40	TI (outcome* N5 evaluat*) OR AB (outcome* N5 evaluat*)	43,464
S39	TI "housing first" OR AB "housing first"	327
S38	TI (((program* or policy or policies or strateg* or scheme* or project* or initiative* or "care package" or training or educat* or pilot or guidance or guideline* or study or pathway or treatment* or promot* or management or "support group" or process* or trial* or intervention*) N5 (evaluat* or effect* or measur* or assess* or experiment* or impact* or feasab* or acceptab* or efficacy or perception* or belief* or uptake or consequence* or attitud* or barrier* or facilit* or motivat* or experience* or implement* or adher* or retention or retain* or reduc* or increas* or improv* or outcome* or cost* or benefit* or interview* or qualitative or ethnograph* or "focus group*"))) OR AB (((program* or policy	1,812,106

For peer review only

	or policies or strateg* or scheme* or project* or initiative* or "care package" or training or educat* or pilot or guidance or guideline* or study or pathway or treatment* or promot* or management or "support group" or process* or trial* or intervention*) N5 (evaluat* or effect* or measur* or assess* or experiment* or impact* or feasab* or acceptab* or efficacy or perception* or belief* or uptake or consequence* or attitud* or barrier* or facilit* or motivat* or experience* or implement* or adher* or retention or retain* or reduc* or increas* or improv* or outcome* or cost* or benefit* or interview* or qualitative or ethnograph* or "focus group*")))	
S37	S35 OR S36	6,735
S36	TI "criminal justice" OR AB "criminal justice"	3,845
S35	TI ((probationer* or parolee* or ((repeat* or ex or re or revolving door or habitual or multiple or former* or previously*) N1 (offen* or convict* or prisoner* or imprison* or incarcerat* or criminal*)) or (former N3 inmate*) or ((community or probation* or parole* or reintegrat*) N4 (prison* or offender* or criminal* or convict* or inmate*)) or ((individuals or men or women) N2 (probation or parole)) or ((reintegrate* or reent* or return*) N3 community))) OR AB ((probationer* or parolee* or ((repeat* or ex or re or revolving door or habitual or multiple or former* or previously*) N1 (offen* or convict* or prisoner* or imprison* or incarcerat* or criminal*)) or (former N3 inmate*) or ((community or probation* or parole* or reintegrat*) N4 (prison* or offender* or criminal* or convict* or inmate*)) or ((individuals or men or women) N2	3,325

For peer review only

	(probation or parole)) or ((reintegrate* or reent* or return*) N3 community)))	
S34	S30 OR S31 OR S32 OR S33	14,314
S33	TI (((hous* or home* or accommodat* or shelter) N3 (insecur* or instability or unstable or stability))) OR AB (((hous* or home* or accommodat* or shelter) N3 (insecur* or instability or unstable or stability)))	2,684
S32	TI homeless* OR AB homeless*	10,454
S31	(MH "Homeless Persons")	6,580
S30	TI ((severe and multiple disadvantage*)) OR AB ((severe and multiple disadvantage*))	22
S29	S19 OR S20 OR S21 OR S22 OR S23 OR S24 OR S25 OR S26 OR S27 OR S28	191,033
S28	TI soda OR AB soda	1,512
S27	(MH "Sweetened Beverages")	933
S26	(MH "Carbonated Beverages")	2,794
S25	TI (((sugary or fizzy or carbonated or soft) N1 drink*)) OR AB (((sugary or fizzy or carbonated or soft) N1 drink*))	2,558
S24	TI (((processed or acidic) N1 food*)) OR AB (((processed or acidic) N1 food*))	2,072
S23	(MH "Diet")	63,635
S22	(MH "Dietary Sucrose")	5,119
S21	TI "sugary food*" OR AB "sugary food*"	193
S20	TI diet* OR AB diet*	155,131

For peer review only

S19	TI (((sugar or sucrose or fructose or glucose) N2 (intake or consum*))) OR AB (((sugar or sucrose or fructose or glucose) N2 (intake or consum*)))	5,433
S18	S6 OR S7 OR S8 OR S9 OR S10 OR S11 OR S12 OR S13 OR S14 OR S15 OR S16 OR S17	326,783
S17	TI ((drug N1 (habit or tak* or hard or illicit or inject*))) OR AB ((drug N1 (habit or tak* or hard or illicit or inject*)))	16,744
S16	TI (((alcohol or drug* or substance*) N2 (misuse* or abuse* or use* or addict* or dependenc* or issue* or problem*))) OR AB (((alcohol or drug* or substance*) N2 (misuse* or abuse* or use* or addict* or dependenc* or issue* or problem*)))	132,015
S15	(MH "Behavior, Addictive")	7,159
S14	(MH "Substance Abusers+")	9,952
S13	(MH "Substance Use Disorders+")	182,899
S12	TI "street drink*" OR AB "street drink*"	6
S11	TI ((alcoholic* N3 (person or people or adult or parent* or family))) OR AB ((alcoholic* N3 (person or people or adult or parent* or family)))	429
S10	(MH "Alcoholics")	730
S9	(MH "Alcoholism")	17,654
S8	(MH "Alcohol Drinking")	33,696
S7	TI ((smoking or cigarette* or tobacco)) OR AB ((smoking or cigarette* or tobacco))	114,101

For peer review only

S6	(MH "Smoking")	65,404
S5	S1 OR S2 OR S3 OR S4	51,836
S4	TI (((dental or oral or tooth or teeth or mouth) N3 (health or care or hygiene or rehabilitation))) OR AB (((dental or oral or tooth or teeth or mouth) N3 (health or care or hygiene or rehabilitation)))	33,755
S3	(MH "Dental Health Services+")	20,382
S2	(MH "Oral Health")	14,782
S1	(MH "Oral Hygiene")	6,534

Scopus – search run on 6th February 2023

2846 results

(TITLE-ABS-KEY ((program* OR policy OR policies OR strateg* OR scheme* OR project* OR initiative* OR "care package" OR training OR educat* OR pilot OR guidance OR guideline* OR study OR pathway OR treatment* OR promot* OR management OR "support group" OR process* OR trial* OR intervention*) W/5 (evaluat* OR effect* OR measur* OR assess* OR experiment* OR impact* OR feasab* OR acceptab* OR efficacy OR perception* OR belief* OR uptake OR consequence* OR attitud* OR barrier* OR facilit* OR motivat* OR experience* OR implement* OR adher* OR retention OR retain* OR reduc* OR increas* OR improv* OR outcome* OR cost* OR benefit* OR interview* OR qualitative OR ethnograph* OR "focus group*") OR "housing first" OR (outcome* W/5 evaluat*))) AND ((TITLE-ABS-KEY ((severe AND multiple AND disadvantage*) OR homeless* OR ((hous* OR home* OR accommodat* OR shelter) W/3 (insecur* OR instability OR unstable OR stability)))) OR ((TITLE-ABS-KEY (probationer* OR parolee* OR "criminal justice")) OR (TITLE-ABS-KEY (former W/3 inmate*)) OR (TITLE-ABS-KEY ((community OR probation* OR parole* OR reintegrat*) W/4 (prison* OR offender* OR criminal* OR convict* OR inmate*))) OR (TITLE-ABS-KEY ((individuals OR men OR women) W/2 (probation OR parole))) OR (TITLE-ABS-KEY ((reintegrate* OR reent* OR return*) W/3 community)) OR (TITLE-ABS-KEY ((repeat* OR ex OR re OR "revolving door" OR habitual OR multiple OR former* OR previously*) W/1 (offen* OR convict* OR prisoner* OR imprison* OR incarcerat* OR criminal*)))))) AND ((TITLE-ABS-KEY ((dental OR oral OR tooth OR teeth OR mouth) W/3 (health OR care OR hygiene OR rehabilitation))) OR (TITLE-ABS-KEY (smoking OR cigarette* OR tobacco)) OR (TITLE-ABS-KEY ("alcohol drinking" OR alcoholism OR (alcoholic* W/3 (person* OR people OR adult OR parent* OR family)) OR "street drink")) OR (TITLE-ABS-KEY ((alcohol OR drug* OR substance*) W/2 (misuse* OR abuse* OR use* OR addict* OR depende

For peer review only

nc* OR issue* OR problem OR disorder*) OR (drug W/1 (habit OR tak* OR hard OR illicit OR inject*)))) OR (TITLE-ABS-KEY ((sugar OR sucrose OR fructose OR glucose) W/2 (intake OR consum*) OR diet* OR "sugary food*" OR ((processed OR acidic) W/1 food*) OR ((sugary OR fizzy OR carbonated OR soft OR sweetened) W/1 (drink* OR beverage*)) OR soda)) AND NOT INDEX (medline))

For peer review only

PRISMA 2020 Checklist

Section and Topic	Item #	Checklist item	Location where item is reported
**TITLE**			
Title	1	Identify the report as a systematic review.	Lines 2-3
**ABSTRACT**			
Abstract	2	See the PRISMA 2020 for Abstracts checklist.	Page 1
**INTRODUCTION**			
Rationale	3	Describe the rationale for the review in the context of existing knowledge.	Lines 38 - 44
Objectives	4	Provide an explicit statement of the objective(s) or question(s) the review addresses.	Lines 54- 56
**METHODS**			
Eligibility criteria	5	Specify the inclusion and exclusion criteria for the review and how studies were grouped for the syntheses.	Table 1
Information sources	6	Specify all databases, registers, websites, organisations, reference lists and other sources searched or consulted to identify studies. Specify the date when each source was last searched or consulted.	Lines 63- 68
Search strategy	7	Present the full search strategies for all databases, registers and websites, including any filters and limits used.	Supplementary file
Selection process	8	Specify the methods used to decide whether a study met the inclusion criteria of the review, including how many reviewers screened each record and each report retrieved, whether they worked independently, and if applicable, details of automation tools used in the process.	Lines 70 -73
Data collection process	9	Specify the methods used to collect data from reports, including how many reviewers collected data from each report, whether they worked independently, any processes for obtaining or confirming data from study investigators, and if applicable, details of automation tools used in the process.	Lines 85 - 91
Data items	10a	List and define all outcomes for which data were sought. Specify whether all results that were compatible with each outcome domain in each study were sought (e.g. for all measures, time points, analyses), and if not, the methods used to decide which results to collect.	Table 1
	10b	List and define all other variables for which data were sought (e.g. participant and intervention characteristics, funding sources). Describe any assumptions made about any missing or unclear information.	Table 1 and Table 2
Study risk of bias assessment	11	Specify the methods used to assess risk of bias in the included studies, including details of the tool(s) used, how many reviewers assessed each study and whether they worked independently, and if applicable, details of automation tools used in the process.	Lines 75 - 83
Effect measures	12	Specify for each outcome the effect measure(s) (e.g. risk ratio, mean difference) used in the synthesis or presentation of results.	Table 2
Synthesis methods	13a	Describe the processes used to decide which studies were eligible for each synthesis (e.g. tabulating the study intervention characteristics and comparing against the planned groups for each synthesis (item #5)).	Table 1
	13b	Describe any methods required to prepare the data for presentation or synthesis, such as handling of missing summary statistics, or data conversions.	Lines 85 -91
	13c	Describe any methods used to tabulate or visually display results of individual studies and syntheses.	Lines 85 -91
	13d	Describe any methods used to synthesize results and provide a rationale for the choice(s). If meta-analysis was performed, describe the model(s), method(s) to identify the presence and extent of statistical heterogeneity, and software package(s) used.	Liness 85 -91
	13e	Describe any methods used to explore possible causes of heterogeneity among study results (e.g. subgroup analysis, meta-regression).	N/A
	13f	Describe any sensitivity analyses conducted to assess robustness of the synthesized results.	N/A
Reporting bias assessment	14	Describe any methods used to assess risk of bias due to missing results in a synthesis (arising from reporting biases).	Lines 75 - 83
Certainty	15	Describe any methods used to assess certainty (or confidence) in the body of evidence for an outcome.	Lines 75 - 83

For peer review only - http://bmjopen.bmj.com/site/about/guidelines.xhtml

PRISMA 2020 Checklist

Section and Topic	Item #	Checklist item	Location where item is reported
assessment			
**RESULTS**			
Study selection	16a	Describe the results of the search and selection process, from the number of records identified in the search to the number of studies included in the review, ideally using a flow diagram.	Figure 1
	16b	Cite studies that might appear to meet the inclusion criteria, but which were excluded, and explain why they were excluded.	Figure 1
Study characteristics	17	Cite each included study and present its characteristics.	Table 2
Risk of bias in studies	18	Present assessments of risk of bias for each included study.	Table 2
Results of individual studies	19	For all outcomes, present, for each study: (a) summary statistics for each group (where appropriate) and (b) an effect estimate and its precision (e.g. confidence/credible interval), ideally using structured tables or plots.	Table 1
Results of syntheses	20a	For each synthesis, briefly summarise the characteristics and risk of bias among contributing studies.	Lines 102 -108
	20b	Present results of all statistical syntheses conducted. If meta-analysis was done, present for each the summary estimate and its precision (e.g. confidence/credible interval) and measures of statistical heterogeneity. If comparing groups, describe the direction of the effect.	N/A
	20c	Present results of all investigations of possible causes of heterogeneity among study results.	N/A
	20d	Present results of all sensitivity analyses conducted to assess the robustness of the synthesized results.	N/A
Reporting biases	21	Present assessments of risk of bias due to missing results (arising from reporting biases) for each synthesis assessed.	Lines 103 - 108
Certainty of evidence	22	Present assessments of certainty (or confidence) in the body of evidence for each outcome assessed.	N/A
**DISCUSSION**			
Discussion	23a	Provide a general interpretation of the results in the context of other evidence.	Lines 223 -251
	23b	Discuss any limitations of the evidence included in the review.	Lines 274 - 277
	23c	Discuss any limitations of the review processes used.	Lines 277 -278
	23d	Discuss implications of the results for practice, policy, and future research.	Lines 281 -288
**OTHER INFORMATION**			
Registration and protocol	24a	Provide registration information for the review, including register name and registration number, or state that the review was not registered.	Lines 58 -59
	24b	Indicate where the review protocol can be accessed, or state that a protocol was not prepared.	Lines 58 -59
	24c	Describe and explain any amendments to information provided at registration or in the protocol.	
Support	25	Describe sources of financial or non-financial support for the review, and the role of the funders or sponsors in the review.	Lines 295 - 300
Competing interests	26	Declare any competing interests of review authors.	Lines 302-304
Availability of data, code and	27	Report which of the following are publicly available and where they can be found: template data collection forms; data extracted from included studies; data used for all analyses; analytic code; any other materials used in the review.	N/A

For peer review only

For peer review only - http://bmjopen.bmj.com/site/about/guidelines.xhtml

PRISMA 2020 Checklist

Section and Topic	Item #	Checklist item	Location where item is reported
other materials			

From: Page MJ, McKenzie JE, Bossuyt PM, Boutron I, Hoffmann TC, Mulrow CD, et al. The PRISMA 2020 statement: an updated guideline for reporting systematic reviews. BMJ 2021;372:n71. doi: 10.1136/bmj.n71

For more information, visit: http://www.prisma-statement.org/

For peer review only
